# Synthesis of functionalized imidazo[4,5-*e*]thiazolo[3,2-*b*]triazines by condensation of imidazo[4,5-*e*]triazinethiones with DMAD or DEAD and rearrangement to imidazo[4,5-*e*]thiazolo[2,3-*c*]triazines

**DOI:** 10.3762/bjoc.17.87

**Published:** 2021-05-14

**Authors:** Alexei N Izmest’ev, Dmitry B Vinogradov, Natalya G Kolotyrkina, Angelina N Kravchenko, Galina A Gazieva

**Affiliations:** 1 N. D. Zelinsky Institute of Organic Chemistry, Russian Academy of Sciences, Leninsky Prosp., 47, Moscow 119991, Russian Federation; 2 National University of Science and Technology (MISiS), 4 Leninsky prosp., Moscow 119049, Russian Federation; 3 Plekhanov Russian University of Economics, 36 Stremyanny Lane, Moscow 117997, Russian Federation

**Keywords:** amidine rearrangement, cyclocondensation, heterocycles, thiazolidine-4-one, 1,2,4-triazine

## Abstract

Two series of functionalized imidazothiazolotriazine derivatives were synthesized via the condensation of imidazo[4,5-*e*]-1,2,4-triazine-3-thiones with acetylenedicarboxylic acid dimethyl and diethyl esters (DMAD and DEAD) and subsequent base-catalyzed rearrangement of the obtained imidazo[4,5-*e*]thiazolo[3,2-*b*]-1,2,4-triazines into regioisomeric imidazo[4,5-*e*]thiazolo[2,3-*c*]-1,2,4-triazine derivatives.

## Introduction

The thiazolidin-4-one heterocyclic system is a well-known, accessible and, as a consequence, a widely used pharmacophore in the chemistry of biologically active compounds possessing antimicrobial [1], antituberculosis [2], anti-inflammatory [3–4], anticancer [5], antidiabetic [6–7], and antiviral activities [8].

**Figure 1:**
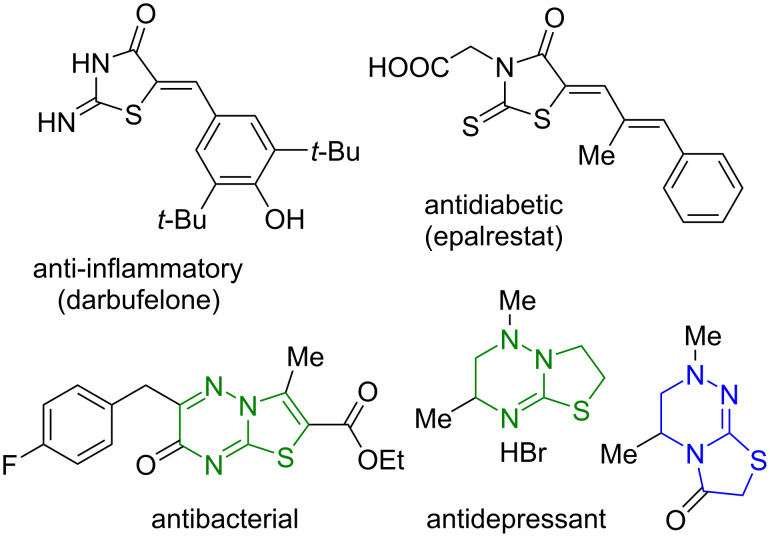
Biologically active compounds having thiazolidin-4-one and thiazolo-1,2,4-triazine units.

A significant number of biologically active thiazolidines amount to their heteroannelated derivatives, namely, condensed thiazolo[3,2-*a*]pyrimidines [9] and thiazolo[3,2-*b*]-1,2,4-triazoles [10], as well as related thiazolo[3,2-*b*]-1,2,4-triazines and thiazolo[2,3-*c*]-1,2,4-triazines possessing antimicrobial, antidepressant, anti-HIV, and anticancer activities [10–15]. Modifications of the position 5 of the thiazolidine cycle often lead to an enhancement of the pharmacological properties of the resulting products, which have received considerable attention in reviews [16–17]. The structures of some of the thiazolidine derivatives and their biological properties are specified in Figure 1.

**Scheme 1:**
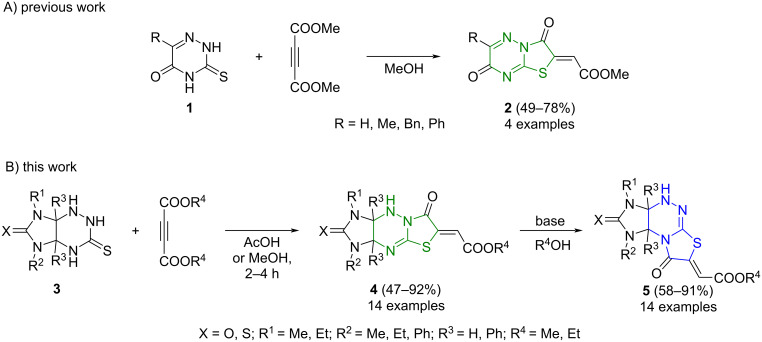
Regioselectivity of the cyclization of 3-thioxo-1,2,4-triazin-5-ones **1** with DMAD (A) and general concept of this work (B).

One of the effective approaches to the preparation of heteroannelated thiazolidin-4-one derivatives consists in the condensation of acetylenedicarboxylic acid esters with heterocyclic compounds containing a thiourea fragment, e.g., pyrimidinethiones [18–19], 1,2,4-triazolethiols [20], and 1,2,4-triazinethiones [21–22]. An important feature of the reactions of dialkyl acetylenedicarboxylates with asymmetric substrates is the high regioselectivity of the cyclizations of intermediate Michael adducts at one of several reactive nitrogen atoms. For example, reported by Giannola et al. [21], the reaction of 3-thioxo-1,2,4-triazin-5-ones **1** with dimethyl acetylenedicarboxylate (DMAD) leads to the only products, namely, thiazolo[3,2-*b*]-1,2,4-triazines **2** (Scheme 1) while the regioisomeric thiazolo[2,3-*с*]-1,2,4-triazine derivatives remain unavailable.

The present work is devoted to the development of methods for the regiodirected synthesis of two series of functionalized imidazothiazolotriazines based on the sequential condensation of imidazo[4,5-*e*]-1,2,4-triazine-3-thiones **3** with DMAD or DEAD and skeletal rearrangement of linear imidazo[4,5-*e*]thiazolo[3,2-*b*]-1,2,4-triazines **4** into isomeric imidazo[4,5-*e*]thiazolo[2,3-*с*]-1,2,4-triazines **5** having an angular structure.

## Results and Discussion

We started by examining the regioselectivity of the condensation of imidazo[4,5-*e*]triazine-3-thiones **3** with DEAD. The effects of temperature, the nature of the solvent, and the structure of the starting substrates on the total yields and ratios of isomeric products **4** and **5** were investigated (Table 1, see also Supporting Information File 1 for details).

**Table 1:** Results for the screening of the reaction conditions^a^.

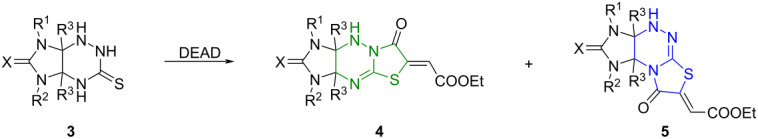						

entry	compound **3**	X, R^1^, R^2^, R^3^	conditions	ratio of **4** and **5**	total yield of **4** + **5** [%]	yield of **4** [%]^b^

1	**3a**	X = O, R^1^ = R^2^ = Me, R^3^ = H	MeOH, reflux, 2 h	38:62	80	–^c^
2			95% EtOH, reflux, 2 h	30:70	72	–^c^
3			Anh. EtOH, reflux, 2 h	32:68	71	–^c^
4			AcOH, 50 °C, 2 h	83:17	72	–^c^
5			AcOH, 40 °C, 2 h	84:16	79	45
6			AcOH, 20 °C, 2 h	91:9	80	53
7	**3b**	X = O, R^1^ = R^2^ = Et, R^3^ = H	MeOH, reflux, 2 h	54:46	68	–^c^
8			AcOH, 20 °C, 2 h	66:34	71	47
9	**3c**	X = O, R^1^ = R^2^ = Me, R^3^ = Ph	MeOH, reflux, 2 h	97:3	81	79
10			95% EtOH, reflux, 2 h	100:0	68	68
11	**3d**	X = O, R^1^ = Me, R^2^ = Ph, R^3^ = H	MeOH, reflux, 2 h	77:23	48	–^c^
12			AcOH, 20 °C, 2 h	100:0	64	64^d^
13			AcOH, 20 °C, 4 h	100:0	86	86
14	**3f**	X = S, R^1^ = Me, R^2^ = Ph, R^3^ = H	MeOH, reflux, 4 h	100:0	52	52
15			AcOH, 20 °C, 4 h	–	0	0

^a^Reaction conditions: stirring the mixture of imidazo[4,5-*e*][1,2,4]triazin-3-thione **3** (2.0 mmol) and DEAD (2.1 mmol) in solvent (4 mL). ^b^Isolated yield. ^c^Pure product **4** was not isolated. ^d^Product **4k** was isolated as a mixture with **3d**.

The reaction of 5,7-dimethylimidazo[4,5-*e*]triazine-3-thione (**3a**) with DEAD proceeded in alcohols with moderate regioselectivity and isomer **5** predominated in the filtered precipitates (Table 1, entries 1–3). The use of absolute or 95% ethanol as a solvent did not significantly affect the total yields of isomers **4** and **5** and their ratio, while carrying out the reaction in acetic acid, as expected [13,23–24], led to a change in regioselectivity and the formation of linear isomer **4** as the main product (Table 1, entries 4–6).

5,7-Diethylimidazo[4,5-*e*]triazine-3-thione (**3b**) reacted with DEAD in a similar manner (Table 1, entries 7 and 8), but with less selectivity. Substrates **3** bearing phenyl substituents at the different positions, vice versa, reacted with DEAD with high selectivity to form imidazo[4,5-*e*]thiazolo[3,2-*b*]triazines **4** as main products (Table 1, entries 9–14). The corresponding isomeric imidazo[4,5-*e*]thiazolo[2,3-*c*]triazines **5** were formed in trace amounts and were detected only in the ^1^H NMR spectra of the evaporated reaction mixtures. The reactions of bicyclic structures **3a**–**g** with dimethyl acetylenedicarboxylate proceeded with similar selectivity. However, the total yields of the corresponding methyl esters were often higher.

**Scheme 2:**
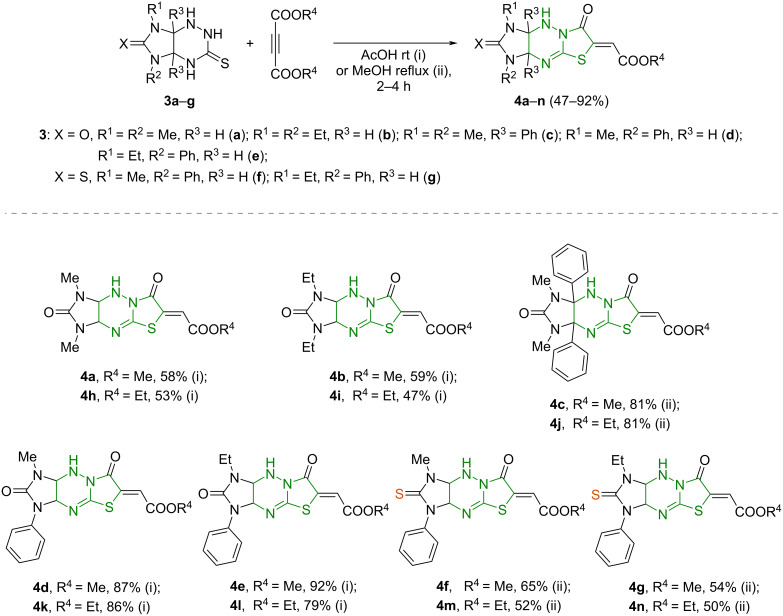
Synthesis of imidazo[4,5-*e*]thiazolo[3,2-*b*]triazine derivatives **4a**–**n** by the reaction of imidazo[4,5-e]triazines **3a**–**g** and DMAD or DEAD.

The optimized conditions found for each group of starting substrates **3a**–**g** were applied to prepare a series of imidazo[4,5-*e*]thiazolo[3,2-*b*]triazines **4a**–**n** with a linear structure (Scheme 2). Precipitates of compounds **4c**–**g**,**j**–**n** bearing phenyl substituents contained no impurities of the corresponding isomers **5**, while the structures **4a**,**b**,**h**,**i** were isolated in individual form only during fractional crystallization from the reaction mixtures.

It was previously shown [24–26] that the products of aldol condensation of imidazo[4,5-*e*]thiazolo[3,2-*b*]triazines with carbonyl compounds, namely, aromatic aldehydes and isatins, are capable of skeletal rearrangement of the thiazolotriazine system proceeding in methanol upon treatment with KOH and resulting in the corresponding isomeric imidazo[4,5-*e*]thiazolo[2,3-*c*]triazine derivatives. In this regard, the possibility of prepearing esters **5** with an angular structure on the basis of directed isomerization of linear imidazo[4,5-*e*]thiazolo[3,2-*b*]triazines **4a**–**n** in basic media has been studied.

**Scheme 3:**
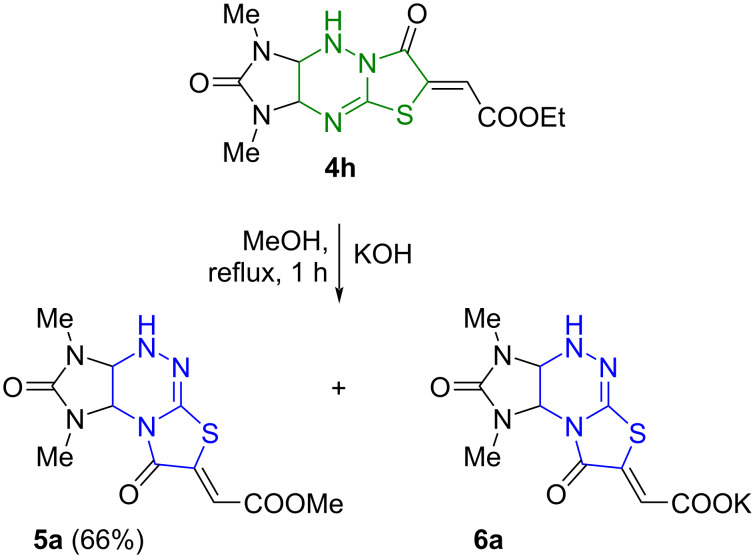
Reaction of imidazo[4,5-*e*]thiazolo[3,2-*b*]triazine **4h** with aqueous KOH.

Indeed, boiling ethyl ester **4h** in methanol in the presence of 0.5 equiv of a 40% KOH aqueous solution resulted in a skeletal rearrangement of the tricyclic system, which, however, was accompanied by reesterification with methanol and partial hydrolysis of the ester group. As a result, the methyl ester **5a** was obtained in 66% yield (Scheme 3).

**Scheme 4:**
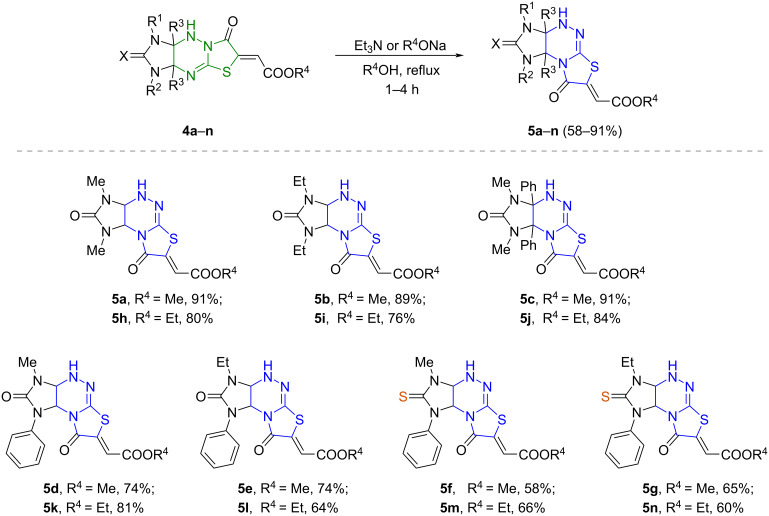
Rearrangement of imidazo[4,5-*e*]thiazolo[3,2-*b*]triazines **4a**–**n** into imidazo[4,5-*e*]thiazolo[2,3-*c*]triazines **5a**–**n**.

Rearrangement of 1,3-dimethyl- and 1,3-diethylimidazo[4,5-*e*]thiazolo[3,2-*b*]triazines **4a**,**b**,**h**,**i** upon treatment with an equivalent amount of triethylamine in corresponding alcohols proceeded without hydrolysis of ester groups and led to the formation of the corresponding regioisomeric derivatives **5a**,**b**,**h**,**i** (Scheme 4). The isomerization of imidazo[4,5-*e*]thiazolo[3,2-*b*]triazines **4c**–**g**,**j**–**n** bearing phenyl groups occurred only upon refluxing the starting compounds in corresponding alcohols in the presence of sodium alcoholates. Imidazo[4,5-*e*]thiazolo[2,3-*c*]triazines **5a**–**n** were synthesized in yields of 58–91%.

**Scheme 5:**
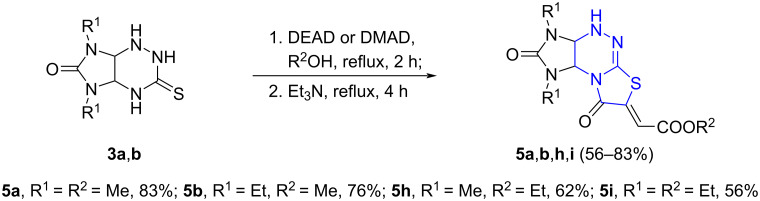
One-pot synthesis of compounds **5a**,**b**,**h**,**i** from imidazo[4,5-*e*]triazines **3a**,**b**.

Because the reaction of imidazo[4,5-*e*]triazines **3a**,**b** with DMAD or DEAD led always to the formation of compounds **4a**,**b**,**h**,**i** along with their isomeric structures **5a**,**b**,**h**,**i**, an attempt was made to sequentially obtain and convert the resulting mixtures of compounds **4** and **5** into the individual isomers **5** in a one-pot mode (Scheme 5).

**Scheme 6:**
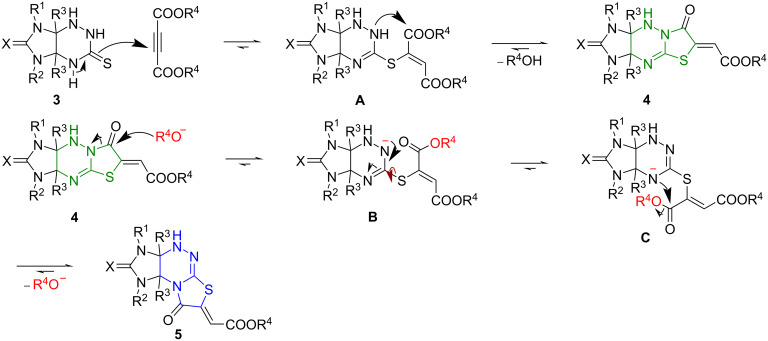
Plausible mechanism of the formation and the rearrangement of compounds **4** into isomers **5**.

The plausible mechanism of the formation of compounds **4** and **5** is detailed in Scheme 6. The reaction of imidazothiazolotriazines **3** with esters of acetylenedicarboxylic acid affords the Michael addition product **A**, which undergoes cyclization involving the nitrogen atom N(2) to give compound **4**. Rearrangement of the latter is, probably, a result of a transamidation reaction upon the treatment with a base, for example, alkoxide anion. The nucleophilic attack of the alkoxide anion leads to the cleavage of the C(7)–N(8) bond to form intermediates **B** and **C** followed by the recyclization of the thiazolidine ring involving the nitrogen atom N(4) [25] to afford the product **5**.

**Figure 2:**
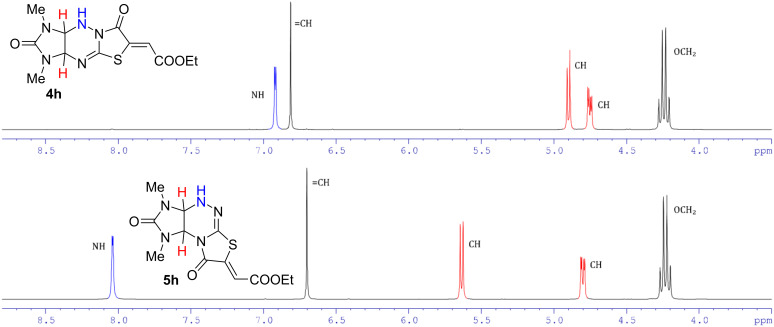
^1^H NMR spectra of compounds **4h** and **5h** in DMSO-*d*_6_ in the region of 3.5–8.8 ppm.

The structures of compounds **4a**–**n** and **5a**–**n** were elucidated by IR, ^1^H and ^13^C NMR, and HRMS spectral data. There are downfield shifts of the NH group proton signal from 6.9–7.2 to 8.0–8.4 ppm in the ^1^H NMR spectra of angular structures **5** in comparison to the spectra of the linear isomers **4**. Downfield shifts from 4.9–5.0 for **4a**,**b**,**h**,**i** to 5.6–5.7 ppm for **5a**,**b**,**h**,**i** and from 5.5–5.7 for **4d**–**g**,**k**–**n** to 6.3–6.5 ppm for **5d**–**g**,**k**–**n** are also observed for the doublet of one of the bridging protons 3a-H in compounds **4** (9a-H in compounds **5**, Figure 2).

**Figure 3:**
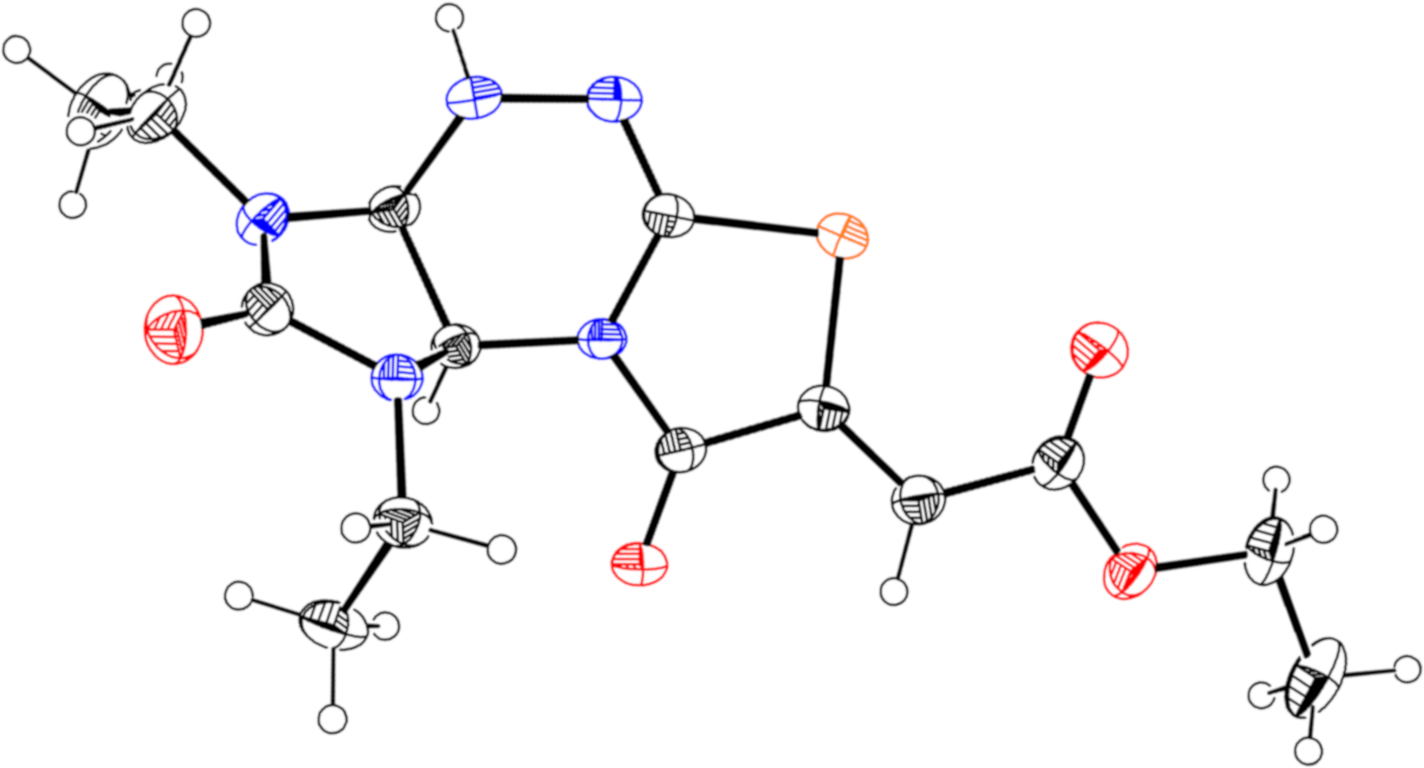
X-ray crystal structure of compound **5i**.

The structure of compound **5i** was additionally confirmed by X-ray diffraction analysis (Figure 3).

## Conclusion

Thus, the conditions for the regioselective preparation of each of the regioisomeric functionalized derivatives of imidazo[4,5-*e*]thiazolo[3,2-*b*]-1,2,4-triazines **4** and imidazo[4,5-*e*]thiazolo[2,3-*c*]-1,2,4-triazines **5** in an individual form were found. The methodology proved to be effective for the synthesis of a wide range of target compounds with various substituents in the tricyclic fragment. Investigations of the antiproliferative activity of the synthesized products **4** and **5**, as well as possible ways of their further transformations in basic media, are continuing.

## Supporting Information

File 1 Experimental and analytical data.

File 2 CIF file for compound **5i**.
