# Supplementary material for: Synthesis of functionalized imidazo[4,5-e]thiazolo[3,2-b]triazines by condensation of imidazo[4,5-e]triazinethiones with DMAD or DEAD and rearrangement to imidazo[4,5-e]thiazolo[2,3-c]triazines
Source: Beilstein J Org Chem. 2021 May 14;17:1141–8. doi: 10.3762/bjoc.17.87 (PMC8144912; doi:10.3762/bjoc.17.87)

BEILSTEIN JOURNAL OF ORGANIC CHEMISTRY

Supporting Information

for

Synthesis of functionalized imidazo[4,5-*e*]thiazolo[3,2-*b*]triazines by condensation of imidazo[4,5-*e*]triazinethiones with DMAD or DEAD and rearrangement to imidazo[4,5-*e*]thiazolo[2,3-*c*]triazines

Alexei N. Izmest'ev, Dmitry B. Vinogradov, Natalya G. Kolotyrkina,
Angelina N. Kravchenko and Galina A. Gazieva

Beilstein J. Org. Chem. **2021,** *17,* 1141–1148. doi:10.3762/bjoc.17.87

Experimental and analytical data

License and Terms: This is a supporting information file under the terms of the Creative Commons Attribution License (https://creativecommons.org/licenses/by/4.0). Please note that the reuse, redistribution and reproduction in particular requires that the author(s) and source are credited and that individual graphics may be subject to special legal provisions.
The license is subject to the *Beilstein Journal of Organic Chemistry* terms and conditions: (https://www.beilstein-journals.org/bjoc/terms)

Table of contents

1. Results for the screening of the reaction conditions S2
2. Experimental S3
3. General procedure for the synthesis of compounds **4a**–**n** S3
4. General procedure for the synthesis of compounds **5a**–**n** S7
5. ^{1}H and ^{13}C NMR spectra of **4a–n** S12
6. ^{1}H and ^{13}C NMR spectra of **5a–n** S26

Table S1 Results for the screening of the reaction conditions[a]

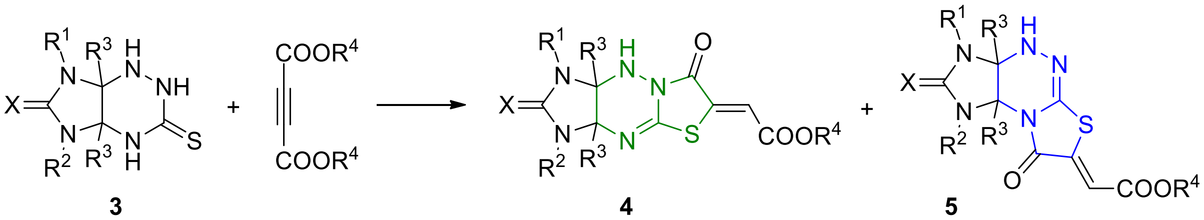

3, 4, 5

Entry	Compound **3**	X	R¹, R², R³	R⁴	Conditions	Ratio of **4** and **5**	Total yield of **4**+**5**, %
1	**3a**	O	R^1=R^2=Me, R^3=H	Et	MeOH, reflux, 2h	38/62	80
2					95% EtOH, reflux, 2 h	30/70	72
3					Anh. EtOH, reflux, 2 h	32/68	71
4					MeOH + HCl, reflux, 2 h	—	0, decomp.
5					AcOH, 50 °C, 2 h	83/17	72
6					AcOH, 40 °C, 2 h	84/16	79
7					AcOH, 20 °C, 2 h	91/9	80
8				Me	AcOH, 20 °C, 2 h	89/11	88
9	**3b**	O	R^1=R^2=Et, R^3=H	Et	MeOH, reflux, 2 h	54/46	68
10					AcOH, 45 °C, 2 h	74/26	47
11					AcOH, 20 °C, 2 h	66/34	71
12				Me	AcOH, 20 °C, 2 h	94/6	63
13	**3c**	O	R^1=R^2=Me, R^3=Ph	Et	MeOH, reflux, 2 h	97/3	81
14					95% EtOH, reflux, 2 h	100/0	68
15					Anh. EtOH, reflux, 2 h	100/0	51
16				Me	MeOH, reflux, 2 h	100/0	81
17	**3d**	O	R^1=Me, R^2=Ph, R^3=H	Et	MeOH, reflux, 2 h	77/23	48[c]
18					AcOH, 20 °C, 2 h	100/0	64[b]
19					AcOH, 20 °C, 4 h	100/0	86
20					AcOH, 20 °C, 6 h	100/0	85
21				Me	AcOH, 20 °C, 4 h	100/0	87
22	**3e**	O	R^1=Et, R^2=Ph, R^3=H	Et	AcOH, 20 °C, 4 h	100/0	79
23				Me	AcOH, 20 °C, 4 h	100/0	92
24	**3f**	S	R^1=Me, R^2=Ph, R^3=H	Et	MeOH, reflux, 4 h	100/0	52
25					AcOH, 20 °C, 4 h	—	0
26					AcOH, 20 °C, 72 h	—	0
27				Me	MeOH, reflux, 4 h	100/0	65
28	**3g**	S	R^1=Et, R^2=Ph, R^3=H	Et	MeOH, reflux, 4 h	100/0	50
29				Me	MeOH, reflux, 4 h	100/0	54

[a]Reaction conditions: stirring the mixture of imidazo[4,5-*e*][1,2,4]triazin-3-thione **3** (2.0 mmol), and DMAD or DEAD (2.1 mmol) in solvent (4 ml). [b]Product **4k** was isolated in the mixture with **3d**. [c]The pure product **4** has not been isolated.

Experimental

IR spectra were recorded on a Bruker Alpha instrument in KBr pellets. ^1H NMR spectra were recorded on Bruker AM300 (300 MHz) and Bruker AV 600 (600 MHz) spectrometers in DMSO-d_6, using TMS as internal standard. ^{13}C NMR spectra were recorded on Bruker AM300 (75 MHz), Bruker DRX-500 (126 MHz), and Bruker AV 600 (151 MHz) spectrometers in DMSO-d_6, using TMS as internal standard. High-resolution mass spectra were obtained on a Bruker micrOTOF II instrument using electrospray ionization. Melting points were determined in open glass capillaries on a Gallenkamp (Sanyo) melting point apparatus.

All the reagents were purchased from Acros Organics and used without further purification. Compounds **3** were prepared according to known procedures for **3a**, **b** [1,2], **3c** [2], and **3d–g** [3].

Introductory experimental paragraph: Give a brief description of apparatus, instrumentation (makes and models), and methods used. Include the sources of any unusual reagents. All known compounds should be indicated by a reference or a CAS registry number.

General procedure for the synthesis of compounds 4a–n

Dimethyl or diethyl acetylenedicarboxylate (DMAD or DEAD, 4.2 mmol) were added to a suspension of imidazotriazine **3a**, **b**, **d**, **e** (4 mmol) in glacial AcOH (8 mL), and the mixture was stirred at room temperature for 2 h (for **4a**, **b**, **h**, **i**) or 4 h (for **4d**, **e**, **k**, **l**). The precipitate of compounds **4a**, **b**, **h**, **i** was filtered off, washed with MeOH and recrystallized from MeOH if necessary.

Dimethyl or diethyl acetylenedicarboxylate (DMAD or DEAD) (4.2 mmol) were added to a refluxing suspension of imidazotriazine **3c**, **f**, **g** (4 mmol) in MeOH (8 mL), and the mixture was refluxed with stirring for 2 h (for **4c**, **j**) or 4 h (for **4f**, **g**, **m**, **n**). The precipitate of compounds **4c**, **f**, **g**, **j**, **m**, **n** was filtered off and washed with MeOH.

Methyl (*Z*)-2-(1,3-dimethyl-2,7-dioxo-1,2,3,3a,9,9a-hexahydroimidazo[4,5-*e*]thiazolo[3,2-*b*][1,2,4]triazin-6(7*H*)-ylidene)acetate (4a)

Yield 722 mg (58%) as a pale yellow solid. Mp: 221–222 °C. IR (KBr), ν 3466, 3434, 3190 (NH), 2951 (Alk), 1724, 1697, 1639, 1616 (C=N, C=O) cm^{-1}. ^1H NMR (300 MHz, DMSO-d_6): δ 2.58 (s, 3 H, NCH_3), 2.77 (s, 3 H, NCH_3), 3.79 (s, 3 H, OCH_3), 4.76 (dd, *J* = 5.9, 2.5 Hz, 1 H, 9a-H), 4.91 (d, *J* = 6.0 Hz, 1 H 3a-H), 6.86 (s, 1 H, =CH), 6.93 (d, *J* = 2.5 Hz, 1 H, NH). ^{13}C NMR (75 MHz, DMSO-d_6): δ 27.34, 28.28 ($2NCH_3$), 53.12 (OCH_3), 65.79, 66.42 (C-3a, C-9a), 116.51 (=CH), 139.79 (C-6), 149.28 (4a-C=N), 159.08, 159.69 (2-C=O, 7-C=O), 166.32 (COOMe). HRMS (ESI): *m/z* $[M + H]^+$ calcd for $C_{11}H_{13}N_5O_4S$: 312.0761; found: 312.0756.

Methyl (*Z*)-2-(1,3-diethyl-2,7-dioxo-1,2,3,3a,9,9a-hexahydroimidazo[4,5-*e*]thiazolo[3,2-*b*][1,2,4]triazin-6(7*H*)-ylidene)acetate (4b)

Yield 719 mg (59%) as a pale yellow solid. Mp: 202–204 °C. IR (KBr), ν 3249 (NH), 2974, 2960, 2936, 2875 (Alk), 1738, 1700, 1646, 1614 (C=N, C=O) cm^{-1}. ^1H NMR (300 MHz, DMSO-d_6): δ 0.94 (t, *J* = 7.2 Hz, 3 H, CH_3), 1.14 (t, *J* = 7.2 Hz, 3 H, CH_3), 3.01–3.19 (m, 3 H, NCH_2), 3.31–3.41 (m, 1 H, NCH_2), 3.78 (s, 3 H, OCH_3), 4.89 (dd, *J* = 6.0, 2.4 Hz, 1 H, 9a-H), 4.96 (d, *J* = 5.9 Hz, 1H 3a-H), 6.86 (s, 1 H, =CH), 6.89 (d, *J* = 2.4 Hz, 1 H, NH). ^{13}C NMR (75 MHz, DMSO-d_6): δ 13.25, 13.88 ($2CH_3$), 34.91, 35.61 ($2NCH_2$), 53.12 (OCH_3), 63.77, 64.73

(C-3a, C-9a), 116.62 (=CH), 139.70 (C-6), 149.27 (4a-C=N), 158.12, 159.68 (2-C=O, 7-C=O), 166.31 (COOMe). HRMS (ESI): *m/z* [*M* + H]$^+$ calcd for $C_{13}H_{17}N_5O_4S$: 340.1074; found: 340.1074.

Methyl (*Z*)-2-(1,3-dimethyl-2,7-dioxo-3a,9a-diphenyl-1,2,3,3a,9,9a-hexahydroimidazo[4,5-*e*]thiazolo[3,2-*b*][1,2,4]triazin-6(7*H*)-ylidene)acetate (4c)

Yield 1502 mg (81%) as a white solid. Mp: 268–270 °C. IR (KBr), ν 3233 (NH), 3054 (Ar), 2945 (Alk), 1719, 1641, 1616 (C=N, C=O) cm^{-1}. ^{1}H NMR (300 MHz, DMSO-*d$_6$*): δ 2.61 (s, 6 H, 2NCH$_3$), 3.83 (s, 3 H, OCH$_3$), 6.72 (d, *J* = 7.4 Hz, 2 H, Ph-2,6), 6.80 (d, *J* = 7.5 Hz, 2 H, Ph-2,6), 6.97 (s, 1 H, =CH), 7.03–7.21 (m, 6 H, 2Ph-3-5), 7.77 (s, 1 H, NH). ^{13}C NMR (75 MHz, DMSO-*d$_6$*): δ 25.78, 26.48 (2NCH$_3$), 53.22 (OCH$_3$), 80.21, 82.44 (C-3a, C-9a), 116.98 (=CH), 126.67, 127.87, 128.12, 128.46, 128.51, 128.77 (2Ph-2-6), 134.27, 134.98 (2Ph-1), 140.20 (C-6), 148.39 (4a-C=N), 158.92, 159.51 (2-C=O, 7-C=O), 166.41 (COOMe). HRMS (ESI): *m/z* [*M* + H]$^+$ calcd for $C_{23}H_{21}N_5O_4S$: 464.1387; found: 464.1385.

Methyl (*Z*)-2-(1-methyl-2,7-dioxo-3-phenyl-1,2,3,3a,9,9a-hexahydroimidazo[4,5-*e*]thiazolo[3,2-*b*][1,2,4]triazin-6(7*H*)-ylidene)acetate (4d)

Yield 1298 mg (87%) as a pale yellow solid. Mp: 257–259 °C. IR (KBr), ν 3216, 3187 (NH), 3061, 3009 (Ar), 2955, 2913 (Alk), 1736, 1680, 1648, 1615 (C=N, C=O) cm^{-1}. ^{1}H NMR (300 MHz, DMSO-*d$_6$*): δ 2.69 (s, 3 H, NCH$_3$), 3.78 (s, 3 H, OCH$_3$), 5.00 (dd, *J* = 5.9, 2.5 Hz, 1 H, 9a-H), 5.65 (d, *J* = 6.1 Hz, 1 H, 3a-H), 6.87 (s, 1 H, =CH), 7.02–7.20 (m, 2 H, Ph-4, NH), 7.34 (t, *J* = 8.0 Hz, 2 H, Ph-3,5), 7.71 (d, *J* = 7.8 Hz, 2 H, Ph-2,6). ^{13}C NMR (75 MHz, DMSO-*d$_6$*): δ 27.53 (NCH$_3$), 52.60 (OCH$_3$), 64.27, 65.02 (C-3a, C-9a), 116.24 (=CH), 119.21 (Ph-2,6), 122.83 (Ph-4), 128.58 (Ph-3,5), 138.45, 139.11 (Ph-1, C-6), 149.58 (4a-C=N), 155.60, 159.10 (2-C=O, 7-C=O), 165.77 (COOMe). HRMS (ESI): *m/z* [*M* + H]$^+$ calcd for $C_{16}H_{15}N_5O_4S$: 374.0918; found: 374.0912.

Methyl (*Z*)-2-(1-ethyl-2,7-dioxo-3-phenyl-1,2,3,3a,9,9a-hexahydroimidazo[4,5-*e*]thiazolo[3,2-*b*][1,2,4]triazin-6(7*H*)-ylidene)acetate (4e)

Yield 1424 mg (92%) as a white solid. Mp: 230–232 °C. IR (KBr), ν 3222 (NH), 3067, 3013 (Ar), 2980, 2960, 2876, 2839 (Alk), 1732, 1690, 1650, 1611 (C=N, C=O) cm^{-1}. ^{1}H NMR (300 MHz, DMSO-*d$_6$*): δ 1.01 (t, *J* = 7.1 Hz, 3 H, CH$_3$), 3.21 (q, *J* = 7.0 Hz, 2 H, NCH$_2$), 3.77 (s, 3 H, OCH$_3$), 5.13 (d, *J* = 6.1 Hz, 1 H, 9a-H), 5.62 (d, *J* = 6.1 Hz, 1 H, 3a-H), 6.88 (s, 1 H, =CH), 7.06 (t, *J* = 7.2 Hz, 1 H, Ph-4), 7.34 (t, *J* = 7.9 Hz, 2 H, Ph-3,5), 7.70 (d, *J* = 8.1 Hz, 2 H, Ph-2,6). ^{13}C NMR (75 MHz, DMSO-*d$_6$*): δ 13.11 (CH$_3$), 35.67 (NCH$_2$), 53.16 (OCH$_3$), 63.74, 64.61 (C-3a, C-9a), 116.95 (=CH), 119.70 (Ph-2,6), 123.37 (Ph-4), 129.12 (Ph-3,5), 138.93, 139,44 (Ph-1, C-6), 150.22 (4a-C=N), 155.54, 159.71 (2-C=O, 7-C=O), 166.31 (COOMe). HRMS (ESI): *m/z* [*M* + H]$^+$ calcd for $C_{17}H_{17}N_5O_4S$: 388.1074; found: 388.1068.

Methyl (*Z*)-2-(1-methyl-7-oxo-3-phenyl-2-thioxo-1,2,3,3a,9,9a-hexahydroimidazo[4,5-*e*]thiazolo[3,2-*b*][1,2,4]triazin-6(7*H*)-ylidene)acetate (4f)

Yield 1012 mg (65%) as a pale yellow powder. Mp: 236–237 °C. IR (KBr), ν 3203, 3167 (NH), 3064, (Ar), 2949, 2918, 2846, (Alk), 1736, 1652, 1616 (C=N, C=O,), 1327 (C=S) cm^{-1}. ^{1}H NMR (300 MHz, DMSO-*d$_6$*): δ 3.01 (s, 1 H, NCH$_3$), 3.79 (s, 1 H, OCH$_3$), 5.34 (dd, *J* = 6.7, 2.9 Hz, 1 H, 9a-H), 5.55 (d, *J* = 6.6 Hz, 1 H, 3a-H), 6.88 (s, 1 H, =CH), 7.23 (d, *J* = 2.9 Hz, 1 H, NH), 7.28 (t, *J* = 7.3 Hz, 1 H, Ph-4), 7.41 (t, *J* = 7.7 Hz, 2 H, Ph-3,5), 7.57 (d, *J* = 7.9 Hz, 2 H, Ph-2,6). ^{13}C NMR (75 MHz, DMSO-*d$_6$*): δ 31.25 (NCH$_3$), 53.18 (OCH$_3$), 68.65, 68.78 (C-3a, C-9a),

116.88 (=CH), 127.00 (Ph-4), 127.73 (Ph-2,6), 128.83 (Ph-3,5), 139.21, 139.44 (Ph-1, C-6), 150.83 (4a-C=N), 159.59 (7-C=O), 166.32 (COOMe), 181.43 (2-C=S). HRMS (ESI): *m/z* [*M* + H]$^+$ calcd for $C_{16}H_{15}N_5O_3S_2$: 390.0689; found: 390.0688.

Methyl (*Z*)-2-(1-ethyl-7-oxo-3-phenyl-2-thioxo-1,2,3,3a,9,9a-hexahydroimidazo[4,5-*e*]thiazolo[3,2-*b*][1,2,4]triazin-6(7*H*)-ylidene)acetate (4g)

Yield 871 mg (54%) as a pale yellow powder. Mp: 225–227 °C. IR (KBr), ν 3216 (NH), 3063, 3002 (Ar), 2977, 2954, 2933, 2843 (Alk), 1733, 1655, 1607, (C=N, C=O), 1325 (C=S) cm^{-1}.

^{1}H NMR (300 MHz, DMSO-*d$_6$*): δ 1.06 (t, *J* = 7.0 Hz, 3 H, CH_3), 3.46–3.71 (m, 2 H, NCH_2), 3.78 (s, 3 H, OCH_3), 5.44 (d, *J* = 6.8 Hz, 1 H, 9a-H), 5.51 (d, *J* = 6.7 Hz, 1 H, 3a-H), 6.90 (s, 1 H, =CH), 7.28 (t, *J* = 7.4 Hz, 1 H, Ph-4), 7.40 (t, *J* = 7.7 Hz, 2 H, Ph-3,5), 7.55 (d, *J* = 7.7 Hz, 2 H, Ph-2,6). ^{13}C NMR (75 MHz, DMSO-*d$_6$*): δ 12.23 (CH_3), 38.75 (NCH_2), 53.20 (OCH_3), 67.16, 68.55 (C-3a, C-9a), 117.12 (=CH), 127.05 (Ph-4), 127.88 (Ph-2,6), 128.82 (Ph-3,5), 139.10, 139.19 (Ph-1, C-6), 150.90 (4a-C=N), 159.69 (7-C=O), 166.31 (COOMe), 180.59 (2-C=S). HRMS (ESI): *m/z* [*M* + H]$^+$ calcd for $C_{17}H_{17}N_5O_3S_2$: 404.0846; found: 404.0842.

Ethyl (*Z*)-2-(1,3-dimethyl-2,7-dioxo-1,2,3,3a,9,9a-hexahydroimidazo[4,5-*e*]thiazolo[3,2-*b*][1,2,4]triazin-6(7*H*)-ylidene)acetate (4h)

Yield 689 mg (53%) as a pale yellow solid. Mp: 221–222 °C. IR (KBr), ν 3187 (NH), 2971, 2939, 2902 (Alk), 1723, 1697, 1641 (C=N, C=O) cm^{-1}. ^{1}H NMR (300 MHz, DMSO-*d$_6$*): δ 1.26 (t, *J* = 7.1 Hz, 3 H, CH_3), 2.60 (s, 3 H, NCH_3), 2.77 (s, 3 H, NCH_3), 4.24 (q, *J* = 7.1 Hz, 2 H, OCH_2), 4.75 (dd, *J* = 5.9, 2.3 Hz, 1 H, 9a-H), 4.90 (d, *J* = 5.9 Hz, 1 H, 3a-H), 6.81 (s, 1 H, =CH), 6.92 (d, *J* = 2.2 Hz, 1 H, NH). ^{13}C NMR (75 MHz, DMSO-*d$_6$*): δ 13.92 (CH_3), 26.86, 27.74 ($2NCH_3$), 61.52 (OCH_2), 65.32, 65.93 (C-3a, C-9a), 116.29 (=CH), 139.11 (C-6), 148.78 (4a-C=N), 158.55, 159.18 (2-C=O, 7-C=O), 165.24 (COOEt). HRMS (ESI): *m/z* [*M* + H]$^+$ calcd for $C_{12}H_{15}N_5O_4S$: 326.0918; found: 326.0913.

Ethyl (*Z*)-2-(1,3-diethyl-2,7-dioxo-1,2,3,3a,9,9a-hexahydroimidazo[4,5-*e*]thiazolo[3,2-*b*][1,2,4]triazin-6(7*H*)-ylidene)acetate (4i)

Yield 664 mg (47%) as a pale yellow solid. Mp: 216–217 °C. IR (KBr), ν 3254 (NH), 3048, 2976, 2936, 2900, 2878 (Alk), 1738, 1697, 1644, 1613 (C=N, C=O) cm^{-1}. ^{1}H NMR (300 MHz, DMSO-*d$_6$*): δ 0.94 (t, *J* = 7.1 Hz, 3 H, CH_3), 1.13 (t, *J* = 7.2 Hz, 3 H, CH_3), 1.25 (t, *J* = 7.1 Hz, 3 H, CH_3), 3.00–3.20 (m, 3 H, NCH_2), 3.32–3.43 (m, 1 H, NCH_2), 4.24 (q, *J* = 7.1 Hz, 2 H, OCH_2), 4.89 (dd, *J* = 6.0, 2.4 Hz, 1 H, 9a-H), 4.96 (d, *J* = 5.9 Hz, 1 H, 3a-H), 6.82 (s, 1 H, =CH), 6.89 (d, *J* = 2.4 Hz, 1 H, NH). ^{13}C NMR (150 MHz, DMSO-*d$_6$*): δ 12.78, 13.41, 13.97 ($3CH_3$), 34.45, 35.16 ($2NCH_2$), 61.59, 63.33, 64.30 (OCH_2, C-3a, C-9a), 116.42 (=CH), 139.13 (C-6), 148.85 (4a-C=N), 157.67, 159.24 (2-C=O, 7-C=O), 165.31 (COOEt). HRMS (ESI): *m/z* [*M* + H]$^+$ calcd for $C_{14}H_{19}N_5O_4S$: 354.1231; found: 354.1225.

Ethyl (*Z*)-2-(1,3-dimethyl-2,7-dioxo-3a,9a-diphenyl-1,2,3,3a,9,9a-hexahydroimidazo[4,5-*e*]thiazolo[3,2-*b*][1,2,4]triazin-6(7*H*)-ylidene)acetate (4j)

Yield 1546 mg (81%) as a pale yellow powder. Mp: 235–237 °C. IR (KBr), ν 3234 (NH), 3062 (Ar), 2981, 2938, 2904 (Alk), 1735, 1697, 1648, 1619 (C=N, C=O) cm^{-1}. ^{1}H NMR (300 MHz, DMSO-*d$_6$*): δ 1.29 (t, *J* = 7.1 Hz, 3 H, CH_3), 2.61 (s, 6 H, $2NCH_3$), 4.29 (q, *J* = 7.1 Hz, 2 H, OCH_2), 6.72 (d, *J* = 7.4 Hz, 2 H, Ph-2,6), 6.81 (d, *J* = 7.6 Hz, 2 H, Ph-2,6), 6.93 (s, 1 H, =CH), 7.01–7.25 (m, 6 H, 2Ph-3-5), 7.76 (s, 1 H, NH). ^{13}C NMR (75 MHz, DMSO-*d$_6$*): δ 13.96 (CH_3), 25.25, 25.96 ($2NCH_3$), 61.64 (OCH_2), 79.72, 81.95 (C-3a, C-9a), 116.75 (=CH), 126.17,

127.33, 127.61, 127.92, 127.97, 128.24 (2Ph-2-6), 133.78, 134.48 (2Ph-1), 139.53 (C-6), 147.89 (4a-C=N), 158.41, 159.00 (2-C=O, 7-C=O), 165.32 (COOEt). HRMS (ESI): *m/z* [*M* + H]$^+$ calcd for $C_{24}H_{23}N_5O_4S$: 478.1544; found: 478.1530.

Ethyl (*Z*)-2-(1-methyl-2,7-dioxo-3-phenyl-1,2,3,3a,9,9a-hexahydroimidazo[4,5-*e*]thiazolo[3,2-*b*][1,2,4]triazin-6(7*H*)-ylidene)acetate (4k)

Yield 1331 mg (86%) as a pale yellow solid. Mp: 221–222 °C. IR (KBr), ν 3278, 3257 (NH), 3106, 3080, 3069, 3012 (Ar), 2978, 2959, 2935, 2913 (Alk), 1737, 1690, 1643, 1624 (C=N, C=O) cm^{-1}. ^{1}H NMR (300 MHz, DMSO-d_6): δ 1.25 (t, *J* = 7.1 Hz, 3 H, CH_3), 2.69 (s, 3 H, NCH_3), 4.23 (q, *J* = 7.1 Hz, 2 H, OCH_2), 5.00 (dd, *J* = 6.1, 2.5 Hz, 1 H, 9a-H), 5.65 (d, *J* = 6.1 Hz, 1 H, 3a-H), 6.84 (s, 1 H, =CH), 7.06 (t, *J* = 7.3 Hz, 1 H, Ph-4), 7.12 (d, *J* = 2.5 Hz, 1 H, NH), 7.34 (t, *J* = 7.4 Hz, 2 H, Ph-3,5), 7.71 (d, *J* = 7.9 Hz, 2 H, Ph-2,6). ^{13}C NMR (125 MHz, DMSO-d_6): δ 14.00 (CH_3), 27.62 (NCH_3), 61.66 (OCH_2), 64.33, 65.10 (C-3a, C-9a), 116.63 (=CH), 119.25 (Ph-2,6), 122.91 (Ph-4), 128.69 (Ph-3,5), 138.55, 139.04 (C-6, Ph-1), 149.72 (4a-C=N), 155.69, 159.23 (2-C=O, 7-C=O), 165.33 (COOEt). HRMS (ESI): *m/z* [*M* + H]$^+$ calcd for $C_{17}H_{17}N_5O_4S$: 388.1074; found: 388.1084.

Ethyl (*Z*)-2-(1-ethyl-2,7-dioxo-3-phenyl-1,2,3,3a,9,9a-hexahydroimidazo[4,5-*e*]thiazolo[3,2-*b*][1,2,4]triazin-6(7*H*)-ylidene)acetate (4l)

Yield 1267 mg (79%) as a pale yellow solid. Mp: 134–136 °C. IR (KBr), ν 3251, 3232, 3205 (NH), 3065, 3029 (Ar), 2980, 2936, 2903, 2875 (Alk), 1741, 1698, 1647, 1617 (C=N, C=O) cm^{-1}. ^{1}H NMR (300 MHz, DMSO-d_6): δ 1.01 (t, *J* = 7.1 Hz, 3 H, CH_3), 1.25 (t, *J* = 7.1 Hz, 3 H, CH_3), 3.21 (q, *J* = 7.2 Hz, 2 H, NCH_2), 4.23 (q, *J* = 7.1 Hz, 2 H, OCH_2), 5.13 (d, *J* = 6.1 Hz, 1 H, 9a-H), 5.62 (d, *J* = 6.1 Hz, 1 H, 3a-H), 6.85 (s, 1 H, =CH), 7.04–7.09 (m, 2 H, Ph-4, NH), 7.34 (t, *J* = 7.9 Hz, 2 H, Ph-3,5), 7.71 (d, *J* = 7.9 Hz, 2 H, Ph-2,6). ^{13}C NMR (75 MHz, DMSO-d_6): δ 13.13, 14.42 (2CH_3), 35.67 (NCH_2), 62.11 (OCH_2), 63.75, 64.61 (C-3a, C-9a), 117.24 (=CH), 119.69 (Ph-2,6), 123.34 (Ph-4), 129.11 (Ph-3,5), 138.95, 139.28 (Ph-1, C-6), 150.24 (4a-C=N), 155.53, 159.72 (2-C=O, 7-C=O), 165.74 (COOEt). HRMS (ESI): *m/z* [*M* + H]$^+$ calcd for $C_{18}H_{19}N_5O_4S$: 402.1231; found: 402.1229.

Ethyl (*Z*)-2-(1-methyl-7-oxo-3-phenyl-2-thioxo-1,2,3,3a,9,9a-hexahydroimidazo[4,5-*e*]thiazolo[3,2-*b*][1,2,4]triazin-6(7*H*)-ylidene)acetate (4m)

Yield 838 mg (52%) as a yellow solid. Mp: 244–246 °C. IR (KBr), ν 3220 (NH), 3057, (Ar), 2979, 2935, 2914 (Alk), 1741, 1695, 1651, 1618 (C=N, C=O), 1323 (C=S) cm^{-1}. ^{1}H NMR (300 MHz, DMSO-d_6): δ 1.26 (t, *J* = 7.1 Hz, 3 H, CH_3), 3.01 (s, 3 H, NCH_3), 4.25 (q, *J* = 7.1 Hz, 2 H, OCH_2), 5.34 (dd, *J* = 6.7, 2.8 Hz, 1 H, 9a-H), 5.55 (d, *J* = 6.6 Hz, 1 H, 3a-H), 6.85 (s, 1 H, =CH), 7.24 (d, *J* = 2.8 Hz, 1 H, NH), 7.28 (t, *J* = 7.3 Hz, 1 H, Ph-4), 7.41 (t, *J* = 7.7 Hz, 2 H, Ph-3,5), 7.57 (d, *J* = 7.7 Hz, 2 H, Ph-2,6). ^{13}C NMR (75 MHz, DMSO-d_6): δ 14.45 (CH_3), 31.24 (NCH_3), 62.13 (OCH_2), 68.66, 68.78 (C-3a, C-9a), 117.18 (=CH), 126.99 (Ph-4), 127.71 (Ph-2,6), 128.83 (Ph-3,5), 139.21, 139.28, (C-6, Ph-1), 150.86 (4a-C=N), 159.61 (7-C=O), 165.78 (COOEt), 181.41 (2-C=S). HRMS (ESI): *m/z* [*M* + H]$^+$ calcd for $C_{17}H_{17}N_5O_3S_2$: 404.0846; found: 404.0846.

Ethyl (*Z*)-2-(1-ethyl-7-oxo-3-phenyl-2-thioxo-1,2,3,3a,9,9a-hexahydroimidazo[4,5-*e*]thiazolo[3,2-*b*][1,2,4]triazin-6(7*H*)-ylidene)acetate (4n)

Yield 834 mg (50%) as a yellow powder. Mp: 223–225 °C. IR (KBr), ν 3189 (NH), 3057 (Ar), 2977, 2934, 2872 (Alk), 1735, 1693, 1647, 1617 (C=N, C=O), 1317 (C=S) cm^{-1}. ^{1}H NMR (300

MHz, DMSO-d_6): δ 1.07 (t, J = 7.0 Hz, CH_3), 1.26 (t, J = 7.1 Hz, CH_3), 3.53–3.76 (m, 2 H, NCH_2), 4.25 (q, J = 7.1 Hz, OCH_2), 5.46 (dd, J = 6.7, 2.7 Hz, 1 H, 9a-H), 5.52 (d, J = 6.7 Hz, 1 H, 3a-H), 6.87 (s, 1 H, =CH), 7.19 (d, J = 2.7 Hz, 1 H, NH), 7.29 (t, J = 7.3 Hz, 1 H, Ph-4), 7.42 (t, J = 7.7 Hz, 2 H, Ph-3,5), 7.57 (t, J = 7.2 Hz, 2 H, Ph-2,6). ^{13}C NMR (75 MHz, DMSO-d_6): δ 12.34, 14.44 ($2CH_3$), 38.75 (NCH_2), 62.15 (OCH2), 67.17, 68.55 (C-3a, C-9a), 117.40 (=CH), 127.02 (Ph-4), 127.85 (Ph-2,6), 128.81 (Ph-3,5), 139.04, 139.12 (Ph-1, C-6), 150.92 (4a-C=N), 159.69 (7-C=O), 165.76 (COOEt), 180.57 (2-C=S). HRMS (ESI): *m/z* [*M* + H]$^+$ calcd for $C_{18}H_{19}N_5O_3S_2$: 418.1002; found: 418.0988.

General procedure for the synthesis of compounds 5a–n

Method A. Triethylamine (0.28 mL, 2 mmol, for **4a**, **b**, **h**, **i**), MeONa as 30% in methanol (0.093 mL, 0.5 mmol, for **4c–g**) or EtONa as 21% in ethanol (0.187 ml, 0.5 mmol, for **4j–n**) were added to a refluxing suspension of ester **4** (2 mmol) in corresponding alcohol (10 mL) (MeOH for **4a–g** or EtOH for **4h–n**), and the mixture was refluxed for 4 h (for **4a**, **b**, **h**, **i**) or 1 h (for **4d–g**, **k–n**). After cooling, the precipitate of compounds **5a–n** was filtered off and washed with MeOH or EtOH.

Method B (one-pot method for the synthesis of **5a**, **b**, **h**, **i**). Dimethyl or diethyl acetylenedicarboxylate (DMAD or DEAD, 2.1 mmol) were added to a refluxing suspension of imidazotriazine **3a**, **b** (2 mmol) in MeOH or EtOH (4 mL), and the mixture was refluxed for 2 h. Then, triethylamine (0.275 mL, 2 mmol) was added to the suspension, and the mixture was refluxed for additional 4 h. After cooling, the precipitate of compounds **5a**, **b**, **h**, **i** was filtered off and washed with MeOH or EtOH.

Methyl (*Z*)-2-(1,3-dimethyl-2,8-dioxo-1,2,3,3a,4,9a-hexahydroimidazo[4,5-*e*]thiazolo[2,3-*c*][1,2,4]triazin-7(8*H*)-ylidene)acetate (5a)

Yield Method A: 566 mg (91%); Method B: 516 mg (83%) as a bright yellow solid. Mp: 214–216 °C. IR (KBr), ν 3303 (NH), 3009, 2958, (Alk), 1707, 1649, 1602 (C=N, C=O) cm^{-1}. ^{1}H NMR (300 MHz, DMSO-d_6): δ 2.63 (s, 3 H, NCH_3), 2.86 (s, 3 H, NCH_3), 3.78 (OCH_3), 4.80 (dd, J = 6.0, 2.1 Hz, 1 H, 3a-H), 5.64 (d, J = 6.0 Hz, 1 H, 9a-H), 6.74 (s, 1 H, =CH), 8.05 (d, J = 2.1 Hz, 1 H, NH). ^{13}C NMR (75 MHz, DMSO-d_6): δ 27.94, 31.34 ($2NCH_3$), 52.51 (OCH_3), 64.02, 66.06 (C-3a, C-9a), 113.14 (=CH), 135.19 (C-7), 142.08 (5a-C=N), 158.97, 162.73 (2-C=O, 8-C=O), 166.23 (COOMe). HRMS (ESI): *m/z* [*M* + H]$^+$ calcd for $C_{11}H_{23}N_5O_4S$: 312.0761; found: 312.0761.

Methyl (*Z*)-2-(1,3-diethyl-2,8-dioxo-1,2,3,3a,4,9a-hexahydroimidazo[4,5-*e*]thiazolo[2,3-*c*][1,2,4]triazin-7(8*H*)-ylidene)acetate (5b)

Yield Method A: 603 mg (89%); Method B: 515 mg (76%) as a orange solid. Mp: 202–204 °C. IR (KBr), ν 3357, 3272 (NH), 2973, 2952, 2934, 2875 (Alk), 1719, 1696, 1637, 1603 (C=N, C=O) cm^{-1}. ^{1}H NMR (300 MHz, DMSO-d_6) δ 1.02 (t, J = 7.1 Hz, 3 H, CH_3), 1.08 (t, J = 7.0 Hz, 3 H, CH_3), 3.01–3.12 (m, 1 H, NCH_2), 3.13–3.30 (m, 2 H, NCH_2), 3.38–3.57 (m, 1 H, NCH_2), 4.88 (dd, J = 6.1, 3.0 Hz, 1 H, 3a-H), 5.71 (d, J = 5.8 Hz, 1 H, 9a-H), 6.76 (s, 1 H, =CH), 8.03 (d, J = 2.1 Hz, 1 H, NH). ^{13}C NMR (75 MHz, DMSO-d_6): 12.65, 12.89 ($2CH_3$), 34.84, 37.73 ($2CH_2$), 52.42 (OCH_3), 61.48, 63.68 (C-3a, C-9a), 113.18 (=CH), 134.83, 141.91 (5a-C=N, C-7), 157.79, 162.66 (2-C=O, 8-C=O), 166.12 (COOMe). HRMS (ESI): *m/z* [*M* + H]$^+$ calcd for $C_{13}H_{17}N_5O_4S$: 340.1074; found: 340.1076.

Methyl (*Z*)-2-(1,3-dimethyl-2,8-dioxo-3a,9a-diphenyl-1,2,3,3a,4,9a-hexahydroimidazo[4,5-*e*]thiazolo[2,3-*c*][1,2,4]triazin-7(8*H*)-ylidene)acetate (5c)

Yield 843 mg (91%) as a bright yellow powder. Mp: 252–254 °C.

IR (KBr), ν 3359, 3310 (NH), 3105, 3056 (Ar), 2954, 2908 (Alk), 1725, 1706, 1686, 1649 (C=N, C=O) cm^{-1}. ^{1}H NMR (300 MHz, DMSO-d_6) δ 2.59 (s, 3 H, NCH_3), 2.85 (s, 3 H, NCH_3), 3.78 (s, 3 H, OCH_3), 6.64 (s, 1 H, =CH), 6.67 (br.s, 3 H, Ph), 7.10 (br.s, 3 H, Ph), 7.15–7.30 (m, 4 H, Ph), 8.41 (s, 1 H, NH). ^{13}C NMR (75 MHz, DMSO-d_6): δ 25.58, 30.76 (2NCH_3), 52.49 (OCH_3), 81.31, 86.89 (C-3a, C-9a), 113.49 (=CH), 127.20, 127.73, 128.03, 128.56, 128.98 (2Ph-2-6), 131.50, 132.66 (2Ph-1), 136.98, 142.33 (5a-C=N, C-7), 158.12, 161.86 (2-C=O, 8-C=O), 166.25 (COOMe). HRMS (ESI): *m/z* $[M + H]^+$ calcd for $C_{23}H_{21}N_5O_4S$: 464.1387; found: 464.1383.

Methyl (*Z*)-2-(3-methyl-2,8-dioxo-1-phenyl-1,2,3,3a,4,9a-hexahydroimidazo[4,5-*e*]thiazolo[2,3-*c*][1,2,4]triazin-7(8*H*)-ylidene)acetate (5d)

Yield 552 mg (74%) as a bright yellow solid. Mp: 250–251 °C. IR (KBr), ν 3299 (NH), 3070, 3058 (Ar), 2992, 2978, 2948 (Alk), 1711, 1694, 1651, 1604 (C=N, C=O) cm^{-1}. ^{1}H NMR (300 MHz, DMSO-d_6) δ 2.68 (s, 3 H, NCH_3), 3.74 (s, 3 H, OCH_3), 5.15 (dd, *J* = 5.9, 1.7 Hz, 1 H, 3a-H), 6.48 (d, *J* = 6.0 Hz, 1 H, 9a-H), 6.55 (s, 1 H, =CH), 7.15 (t, *J* = 7.2 Hz, 1 H, Ph-4), 7.31 (t, *J* = 7.8 Hz, 2 H, Ph-3,5), 7.40 (d, *J* = 7.7 Hz, 2 H, Ph-2,6), 8.25 (d, *J* = 1.7 Hz, 1 H, NH). ^{13}C NMR (75 MHz, DMSO-d_6): 28.12 (NCH_3), 52.92 (OCH_3), 65.31, 65.31 (C-3a, C-9a), 113.72 (=CH), 124.62 (Ph-2,6), 125.52 (Ph-4), 128.63 (Ph-3,5), 136.13 (Ph-1), 139.11, 142.08 (5a-C=N, C-7), 157.25, 161.98 (2-C=O, 8-C=O), 166.62 (COOMe).

HRMS (ESI): *m/z* $[M + H]^+$ calcd for $C_{16}H_{15}N_5O_4S$: 374.0918; found: 374.0915.

Methyl (*Z*)-2-(3-ethyl-2,8-dioxo-1-phenyl-1,2,3,3a,4,9a-hexahydroimidazo[4,5-*e*]thiazolo[2,3-*c*][1,2,4]triazin-7(8*H*)-ylidene)acetate (5e)

Yield 573 mg (74%) as a bright yellow solid. Mp: 230–232 °C. IR (KBr), ν 3306, 3258 (NH), 3153, 3072 (Ar), 2992, 2973, 2948, 2849 (Alk), 1714, 1692, 1650, 1600 (C=N, C=O) cm^{-1}. ^{1}H NMR (300 MHz, DMSO-d_6): δ 1.04 (t, *J* = 7.1 Hz, 3 H, CH_3), 3.03–3.19 (m, 1 H, NCH_2), 3.20–3.32 (m, 1 H, NCH_2), 3.73 (s, 3 H, OCH_3), 5.26 (d, *J* = 6.0 Hz, 1 H, 3a-H), 6.44 (d, *J* = 5.9 Hz, 1 H, 9a-H), 6.54 (s, 1 H, =CH), 7.15 (t, *J* = 7.3 Hz, 1 H, Ph-4), 7.30 (t, *J* = 7.8 Hz, 2 H, Ph-3,5), 7.40 (d, *J* = 8.0 Hz, 2 H, Ph-2,6), 8.23 (d, *J* = 2.1 Hz, 1 H, NH). ^{13}C NMR (125 MHz, DMSO-d_6): δ 12.45 (CH_3), 34.88 (NCH_2), 52.50 (OCH_3), 62.65, 65.00 (C-3a, C-9a), 113.36 (=CH), 124.11 (Ph-2,6), 125.04 (Ph-4), 128.22 (Ph-3,5), 135.65 (Ph-1), 138.65, 141.62 (C-7, 5a-C=N), 156.10, 161.54 (2-C=O, 8-C=O), 166.19 (COOEt). HRMS (ESI): *m/z* $[M + H]^+$ calcd for $C_{17}H_{17}N_5O_4S$: 388.1074; found: 388.1062.

Methyl (*Z*)-2-(3-methyl-8-oxo-1-phenyl-2-thioxo-1,2,3,3a,4,9a-hexahydroimidazo[4,5-*e*]thiazolo[2,3-*c*][1,2,4]triazin-7(8*H*)-ylidene)acetate (5f)

Yield 451 mg (58%) as a bright yellow solid. Mp: 210–212 °C. IR (KBr), ν 3234 (NH), 3219, 3074, 3055 (Ar), 2955, 2949, 2845 (Alk), 1726, 1694, 1646, 1604 (C=N, C=O), 1318 (C=S) cm^{-1}. ^{1}H NMR (300 MHz, DMSO-d_6): δ 3.06 (s, 3 H, NCH_3), 3.75 (s, 3 H, OCH_3), 5.42 (dd, *J* = 6.6, 2.9 Hz, 1 H, 3a-H), 6.38 (d, *J* = 6.5 Hz, 1 H, 9a-H), 6.53 (s, 1 H, =CH), 7.23–7.42 (m, 5 H, Ph), 8.36 (d, *J* = 2.2 Hz, 1 H, NH). ^{13}C NMR (150 MHz, DMSO-d_6): δ 31.56 (NCH_3), 52.55 (OCH_3), 67.33, 67.56 (C-3a, C-9a), 113.60 (=CH), 127.42 (Ph-4), 128.43, 128.64 (Ph-2,3,5,6),

135.76 (Ph-1), 139.43, 141.43 (5a-C=N, C-7), 161.09 (8-C=O), 166.12 (COOMe), 182.87 (2-C=S). HRMS (ESI): *m/z* [*M* + H]$^+$ calcd for $C_{16}H_{15}N_5O_3S_2$: 390.0689; found: 390.0678.

Methyl (*Z*)-2-(3-ethyl-8-oxo-1-phenyl-2-thioxo-1,2,3,3a,4,9a-hexahydroimidazo[4,5-*e*]thiazolo[2,3-*c*][1,2,4]triazin-7(8*H*)-ylidene)acetate (5g)

Yield 524 mg (65%) as a yellow solid. Mp: 208–210 °C. IR (KBr), ν 3236 (NH), 3131, 3067 (Ar), 2993, 2970, 2952, 2935, 2907, 2874, 2848 (Alk), 1732, 1684, 1652, 1604 (C=N, C=O), 1333 (C=S) cm^{-1}. ^{1}H NMR (300 MHz, DMSO-d_6): δ 1.13 (t, *J* = 7.0 Hz, 3 H, CH_3), 3.41–3.58 (m, 1 H, NCH_2), 3.74 (s, 3 H, OCH_3), 3.74–3.89 (m, 1 H, NCH_2), 5.52 (d, *J* = 7.0 Hz, 1 H, 3a-H), 6.34 (d, *J* = 6.6 Hz, 1 H, 9a-H), 6.52 (s, 1 H, =CH), 7.21–7.4 (m, 5 H, Ph), 8.36 (d, *J* = 2.2 Hz, 1 H, NH). ^{13}C NMR (125 MHz, DMSO-d_6): δ 11.86 (CH_3), 38.60 (NCH_2), 52.55 (OCH_3), 65.70, 67.64 (C-3a,C-9a), 113.71 (=CH), 127.36 (Ph-4), 128.38, 128.67 (Ph-2,3,5,6), 135.90 (Ph-1), 139.39, 141.39 (C-7, 5a-C=N), 161.02 (8-C=O), 166.11 (COOMe), 181.85 (2-C=S). HRMS (ESI): *m/z* [*M* + H]$^+$ calcd for $C_{17}H_{17}N_5O_3S_2$: 404.0846; found: 404.0842.

Ethyl (*Z*)-2-(1,3-dimethyl-2,8-dioxo-1,2,3,3a,4,9a-hexahydroimidazo[4,5-*e*]thiazolo[2,3-*c*][1,2,4]triazin-7(8*H*)-ylidene)acetate (5h)

Yield Method A: 520 mg (80%); Method B: 403 mg (62%) as a bright orange solid. Mp: 201–202 °C. IR (KBr), ν 3337 (NH), 2977, 2938, (Alk), 1734, 1707, 1678, 1640 (C=N, C=O) cm^{-1}. ^{1}H NMR (300 MHz, DMSO-d_6): δ 1.25 (t, *J* = 7.1 Hz, 3 H, CH_3), 2.63 (s, 3 H, NCH_3), 2.68 (s, 3 H, NCH_3), 4.24 (q, *J* = 7.1 Hz, 2 H, OCH_2), 4.80 (dd, *J* = 6.0, 2.0 Hz, 1 H, 3a-H), 5.64 (d, *J* = 6.0 Hz, 1 H, 9a-H), 6.70 (s, 1 H, =CH), 8.04 (d, *J* = 2.0 Hz, 1 H, NH). ^{13}C NMR (75 MHz, DMSO-d_6): δ 13.98 (CH_3), 27.86, 31.26 ($2NCH_3$), 61.30 (OCH_2), 63.95, 63.98 (C-3a,C-9a), 113.39 (=CH), 135.17, 141.84 (C-7, 5a-C=N), 158.87, 162.86 (2-C=O, 8-C=O), 165.64 (COOEt). HRMS (ESI): *m/z* [*M* + H]$^+$ calcd for $C_{12}H_{15}N_5O_4S$: 326.0918; found: 326.0914.

Ethyl (*Z*)-2-(1,3-diethyl-2,8-dioxo-1,2,3,3a,4,9a-hexahydroimidazo[4,5-*e*]thiazolo[2,3-*c*][1,2,4]triazin-7(8*H*)-ylidene)acetate (5i)

Yield Method A: 537 mg (76%); Method B: 395 mg (56%) as a bright yellow solid. Mp: 150–152 °C. IR (KBr), ν 3281 (NH), 2982, 2941, 2879, (Alk), 1737, 1714, 1642, 1616 (C=N, C=O) cm^{-1}. ^{1}H NMR (300 MHz, DMSO-d_6): δ 0.90–1.16 (m, 6 H, $2CH_3$), 1.26 (t, *J* = 7.1 Hz, 3 H, CH_3), 2.90–3.10 (m, 1 H, NCH_2), 3.11–3.30 (m, 2 H, NCH_2), 3.35–3.53 (m, 1 H, NCH_2), 4.24 (q, *J* = 7.1 Hz, 2 H, OCH_2), 4.87 (dd, *J* = 5.9, 2.1 Hz, 1 H, 3a-H), 5.70 (d, *J* = 5.9 Hz, 1 H, 9a-H), 6.72 (s, 1 H, =CH), 8.02 (s, 1 H, NH). ^{13}C NMR (75 MHz, DMSO-d_6): δ 12.67, 12.92, 13.99 ($3CH_3$), 34.85, 37.76 ($2NCH_2$), 61.32, 61.60 (C-3a, C-9a), 63.69 (OCH_2), 113.50 (=CH), 134.94 (C-7), 141.78 (5a-C=N), 157.81, 162.69 (2-C=O, 8-C=O), 165.64 (COOEt). HRMS (ESI): *m/z* [*M* + H]$^+$ calcd for $C_{14}H_{19}N_5O_4S$: 354.1231; found: 354.1222.

Ethyl (*Z*)-2-(1,3-dimethyl-2,8-dioxo-3a,9a-diphenyl-1,2,3,3a,4,9a-hexahydroimidazo[4,5-*e*]thiazolo[2,3-*c*][1,2,4]triazin-7(8*H*)-ylidene)acetate (5j)

Yield 801 mg (84%) as a bright yellow solid. Mp: 236–238 °C. IR (KBr), ν 3296 (NH), 3144, 3056 (Ar), 2973, 2908, (Alk), 1726, 1703, 1651, 1602 (C=N, C=O) cm^{-1}. ^{1}H NMR (300 MHz, DMSO-d_6): δ 1.25 (t, *J* = 7.1 Hz, 3 H, CH_3), 2.59 (s, 3 H, NCH_3), 2.85 (s, 3 H, NCH_3), 4.25 (q, *J* = 7.1 Hz, 2 H, OCH_2), 6.61 (s, 1 H, =CH), 6.68 (br.s, 3 H, Ph), 7.10 (br.s, 3 H, Ph), 7.18–7.24 (m, 4 H, Ph), 8.40 (s, 1 H, NH). ^{13}C NMR (75 MHz, DMSO-d_6): δ 14.02 (CH_3), 25.52, 30.73 ($2NCH_3$), 61.28 (OCH_2), 81.31, 86.81 (C-3a, C-9a), 113.70 (=CH), 127.16, 127.68, 127.97, 128.49, 128.90 (2Ph-2-6), 131.50, 132.69, (2Ph-1), 136.91, 142.18 (C-7, 5a-C=N), 158.04,

161.82 (2-C=O, 8-C=O), 165.68 (COOEt). HRMS (ESI): *m/z* [*M* + H]+ calcd for $C_{24}H_{23}N_5O_4S$: 478.1544; found: 478.1550.

Ethyl (*Z*)-2-(3-methyl-2,8-dioxo-1-phenyl-1,2,3,3a,4,9a-hexahydroimidazo[4,5-*e*]thiazolo[2,3-*c*][1,2,4]triazin-7(8*H*)-ylidene)acetate (5k)

Yield 627 mg (81%) as a bright yellow solid. Mp: 240–242 °C. IR (KBr), ν 3295 (NH), 3077, (Ar), 2982, 2941, 2913 (Alk), 1716, 1690, 1650, 1604 (C=N, C=O) cm^{-1}. ^1H NMR (300 MHz, DMSO-d_6): δ 1.22 (t, *J* = 7.1 Hz, 3 H, CH_3), 2.68 (s, 3 H, NCH_3), 4.20 (q, *J* = 7.1 Hz, 2 H, OCH_2), 5.15 (dd, *J* = 5.9, 2.0 Hz, 1 H, 3a-H), 6.47 (d, *J* = 6.0 Hz, 1 H, 9a-H), 6.51 (s, 1 H, =CH), 7.15 (t, *J* = 7.2 Hz, 1 H, Ph-4), 7.31 (t, *J* = 7.7 Hz, 2 H, Ph-3,5), 7.39 (d, *J* = 7.9 Hz, 2 H, Ph-2,6), 8.23 (d, *J* = 1.8 Hz, 1 H, NH). ^{13}C NMR (75 MHz, DMSO-d_6): δ 14.47 (CH_3), 28.12 (NCH_3), 61.78 (OCH_2), 65.21, 65.34 (C-3a, C-9a), 114.00 (=CH), 124.67 (Ph-2,6), 125.52 (Ph-4), 128.63 (Ph-3,5), 136.20 (Ph-1), 139.10, 141.94 (5a-C=N, C-7), 157.24, 161.99 (2-C=O, 8-C=O), 166.10 (COOEt). HRMS (ESI): *m/z* [*M* + H]+ calcd for $C_{17}H_{17}N_5O_4S$: 388.1074; found: 388.1073.

Ethyl (*Z*)-2-(3-ethyl-2,8-dioxo-1-phenyl-1,2,3,3a,4,9a-hexahydroimidazo[4,5-*e*]thiazolo[2,3-*c*][1,2,4]triazin-7(8*H*)-ylidene)acetate (5l)

Yield 513 mg (64%) as a bright yellow solid. Mp: 220–222 °C. IR (KBr), ν 3256 (NH), 3141, 3077 (Ar), 2979, 2934, 2873 (Alk), 1721, 1689, 1650, 1603 (C=N, C=O) cm^{-1}. ^1H NMR (300 MHz, DMSO-d_6): δ 1.04 (t, *J* = 7.0 Hz, 3 H, CH_3), 1.22 (t, *J* = 7.1 Hz, 3 H, CH_3), 3.03–3.19 (m, 1 H, NCH_2), 3.20–3.28 (m, 1 H, NCH_2), 4.20 (q, *J* = 7.0 Hz, 2 H, OCH_2), 5.27 (d, *J* = 6.2 Hz, 1 H, 3a-H), 6.44 (d, *J* = 5.9 Hz, 1 H, 9a-H), 6.51 (s, 1 H, =CH), 7.15 (t, *J* = 7.1 Hz, 1 H, Ph-4), 7.31 (t, *J* = 7.7 Hz, 2 H, Ph-3,5), 7.40 (d, *J* = 7.6 Hz, 2 H, Ph-2,6). ^{13}C NMR (75 MHz, DMSO-d_6): δ 12.36, 13.93 ($2CH_3$), 34.76 (NCH_2), 61.23, 62.62, 64.92 (OCH_2, C-3a, C-9a), 113.50 (=CH), 124.04 (Ph-2,6), 124.91 (Ph-4), 128.07 (Ph-3,5), 135.54 (Ph-1), 138.55, 141.36 (5a-C=N, C-7), 155.97, 161.41 (2-C=O, 8-C=O), 165.52 (COOEt). HRMS (ESI): *m/z* [*M* + H]+ calcd for $C_{18}H_{19}N_5O_4S$: 402.1231; found: 402.1223.

Ethyl (*Z*)-2-(3-methyl-8-oxo-1-phenyl-2-thioxo-1,2,3,3a,4,9a-hexahydroimidazo[4,5-*e*]thiazolo[2,3-*c*][1,2,4]triazin-7(8*H*)-ylidene)acetate (5m)

Yield 532 mg (66%) as a bright yellow powder. Mp: 219–221 °C. IR (KBr), ν 3315 (NH), 3077 (Ar), 2980, 2933, 2915, 2900 (Alk), 1729, 1681, 1647, 1604 (C=N, C=O), 1315 (C=S) cm^{-1}. ^1H NMR (300 MHz, DMSO-d_6): δ 1.23 (t, *J* = 7.1 Hz, 3 H, CH_3), 3.05 (s, 3 H, NCH_3), 4.21 (q, *J* = 7.0 Hz, 2 H, OCH_2), 5.41 (dd, *J* = 6.4, 2.1 Hz, 1 H, 3a-H), 6.37 (d, *J* = 6.5 Hz, 1 H, 9a-H), 6.48 (s, 1 H, =CH), 7.23–7.40 (m, 5 H, Ph), 8.34 (s, 1 H, NH). ^{13}C NMR (75 MHz, DMSO-d_6): δ 14.47 (CH_3), 31.99 (NCH_3), 61.86 (OCH_2), 67.76, 68.00 (C-3a, C-9a), 114.32 (=CH), 127.84 (Ph-4), 128.86 (Ph-2,6), 129.09 (Ph-3,5), 136.21 (Ph-1), 139.87, 141.71 (5a-C=N, C-7), 161.53 (8-C=O), 166.04 (COOEt), 183.30 (2-C=S). HRMS (ESI): *m/z* [*M* + H]+ calcd for $C_{17}H_{17}N_5O_3S_2$: 404.0846; found: 404.0840.

Ethyl (*Z*)-2-(3-ethyl-8-oxo-1-phenyl-2-thioxo-1,2,3,3a,4,9a-hexahydroimidazo[4,5-*e*]thiazolo[2,3-*c*][1,2,4]triazin-7(8*H*)-ylidene)acetate (5n)

Yield 500 mg (60%) as a bright yellow solid. Mp: 192–194 °C. IR (KBr), ν 3346 (NH), 3084, 3062 (Ar), 2984, 2940, 2904, 2874 (Alk), 1727, 1672, 1655, 1602 (C=N, C=O), 1319 (C=S) cm^{-1}. ^1H NMR (300 MHz, DMSO-d_6): δ 1.13 (t, *J* = 7.0 Hz, 3 H, CH_3), 1.22 (t, *J* = 7.1 Hz, 3 H, CH_3), 3.41–3.53 (m, 1 H, CH_2), 3.66–3.88 (m, 1 H, CH_2), 4.20 (q, *J* = 7.0 Hz, 2 H, OCH_2), 5.52 (d, *J* = 6.6 Hz, 1 H, 3a-H), 6.34 (d, *J* = 6.6 Hz, 1 H, 9a-H), 6.48 (s, 1 H, =CH), 7.20–7.42 (m, 5 H,

Ph), 8.36 (s, 1 H, NH). ^{13}C NMR (75 MHz, DMSO-d_6): δ 12.27, 14.46 ($2CH_3$), 39.01 (NCH_2), 61.89 (OCH_2), 66.12, 68.05 (C-3a, C-9a), 114.42 (=CH), 127.79 (Ph-4), 128.81 (Ph-2,6), 129.09 (Ph-3,5), 136.48, 139.80, 141.65 (Ph-1, C-7, 5a-C=N), 161.45 (8-C=O), 166.03 (COOEt), 182.24 (2-C=S). HRMS (ESI): *m/z* [*M* + H]$^+$ calcd for $C_{18}H_{19}N_5O_3S_2$: 418.1002; found: 418.1000.

X-ray diffraction data of compound 5i were collected at 100 K on a Bruker Quest D8 diffractometer equipped with a Photon-III area-detector (graphite monochromator, shutterless φ- and ω-scan technique), using MoK_α-radiation. The intensity data were integrated by the SAINT program [4] and corrected for absorption and decay using SADABS [5]. The structure was solved by direct methods using SHELXT [6] and refined on F^2 using SHELXL-2018 [7]. All non-hydrogen atoms were refined with anisotropic displacement parameters. Hydrogen atoms were placed in ideal calculated positions as riding atoms with relative isotropic displacement parameters; bond distances to H-atoms were refined. Full crystallographic data have been deposited at the Cambridge Crystallographic Data Center (deposit CCDC 2069601).

References

1. Sigachev, A. S.; Kravchenko, A. N.; Belyakov, P. A.; Lebedev, O. V.; Makhova, N. N. *Russ. Chem. Bull.* **2006**, *55*, 865.
2. Sigachev, A. S.; Kravchenko, A. N.; Lyssenko, K. A.; Belyakov, P. A.; Lebedev, O. V.; Makhova, N. N. *Mendeleev Commun.* **2003**, *13*, 190.
3. Gazieva, G. A.; Karpova, T. B.; Nechaeva, T. V.; Nelyubina, Y. V.; Zanin, I. E.; Kravchenko, A. N. *Synlett.* **2017**, *28*, 858.
4. Bruker. APEX-III. Bruker AXS Inc., Madison, Wisconsin, USA, 2019.
5. Krause, L.; Herbst-Irmer, R.; Sheldrick, G. M.; Stalke, D. *J. Appl. Cryst.* **2015**, *48*, 3.
6. Sheldrick, G. M. *Acta Cryst*. **2015**, *A71*, 3.
7. Sheldrick, G. M. *Acta Cryst*. **2015**, *C71*, 3.

^{1}H NMR spectrum of **4a**

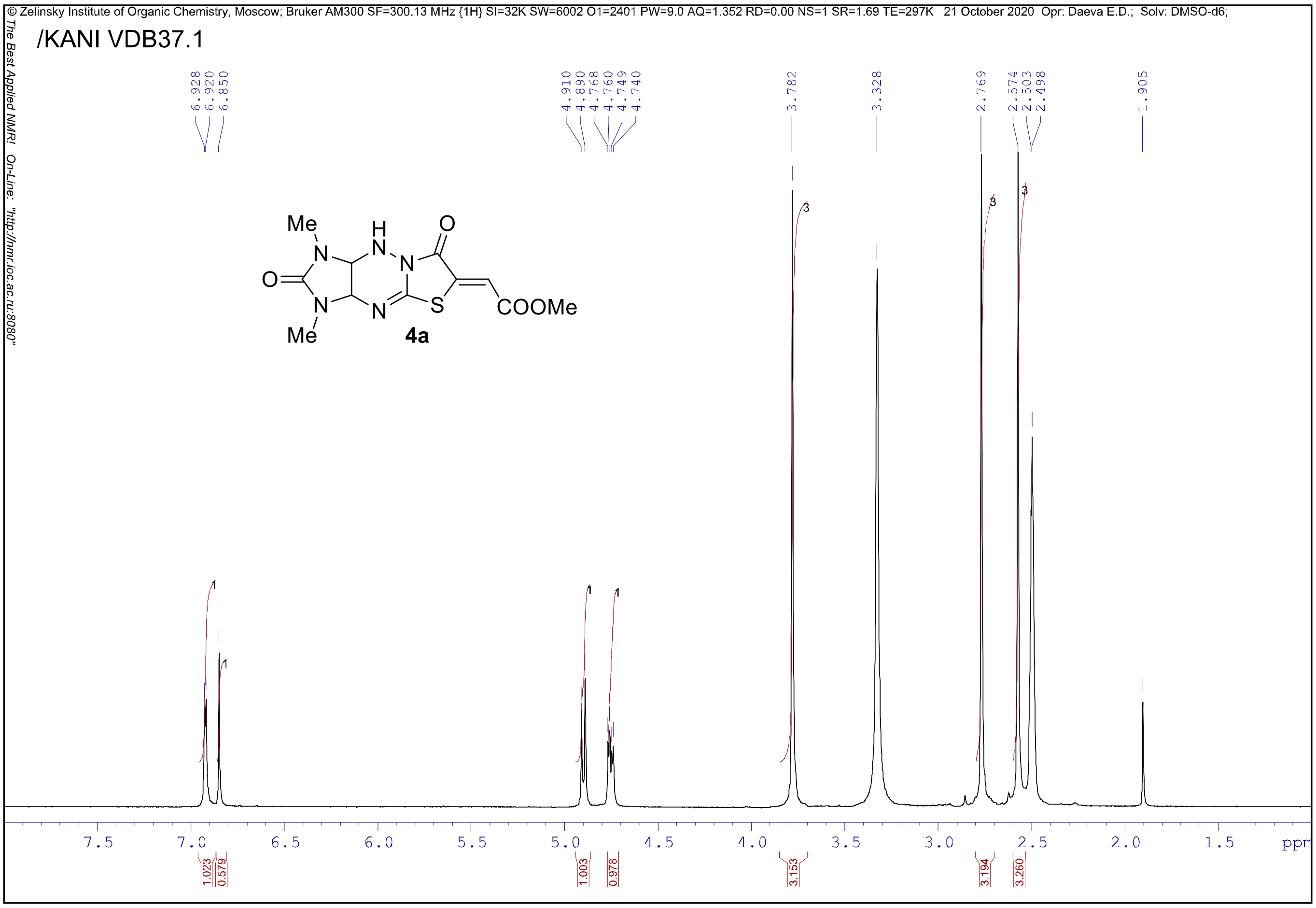

^{13}C NMR spectrum of **4a**

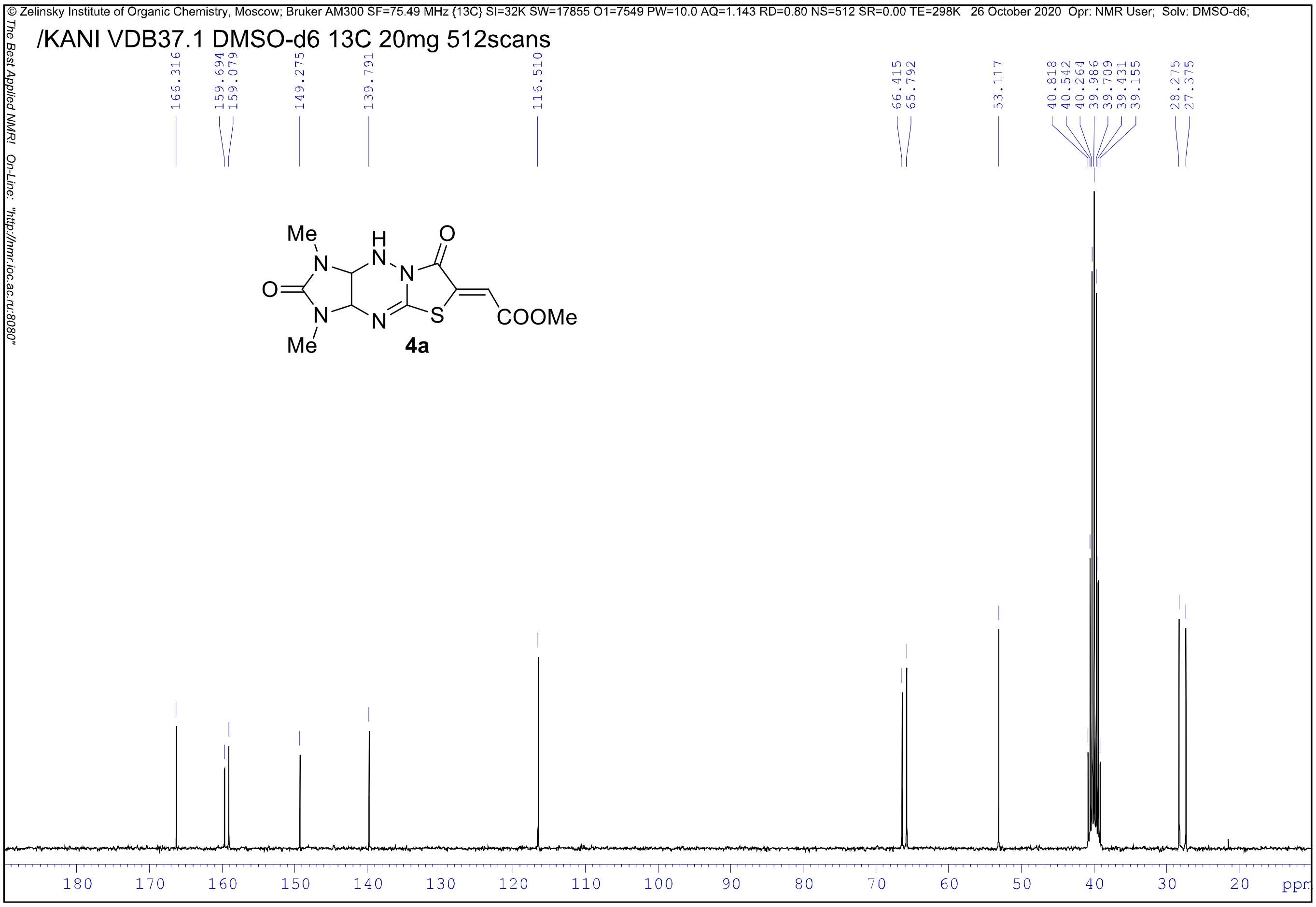

^{1}H NMR spectrum of **4b**

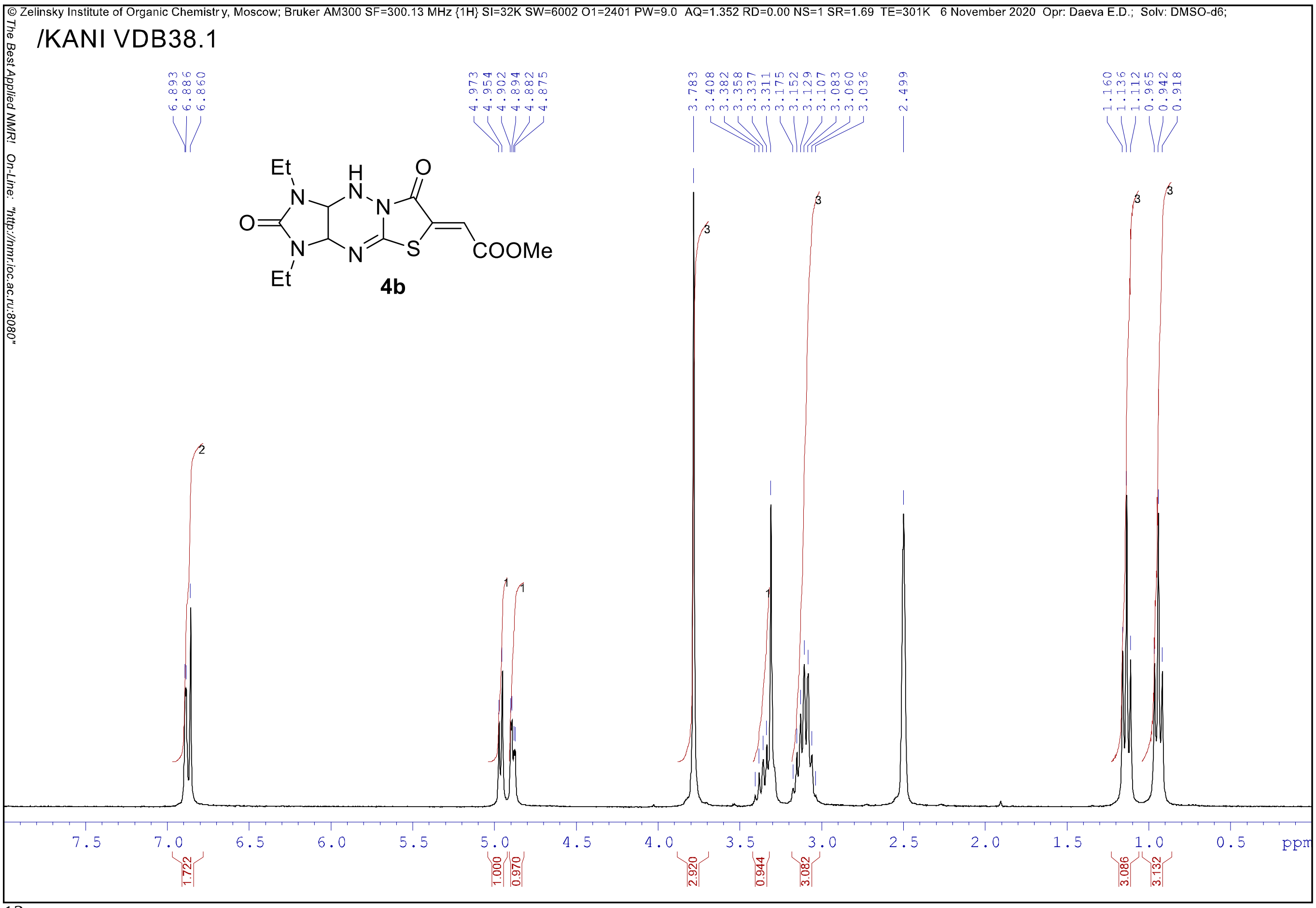

^{13}C NMR spectrum of **4b**

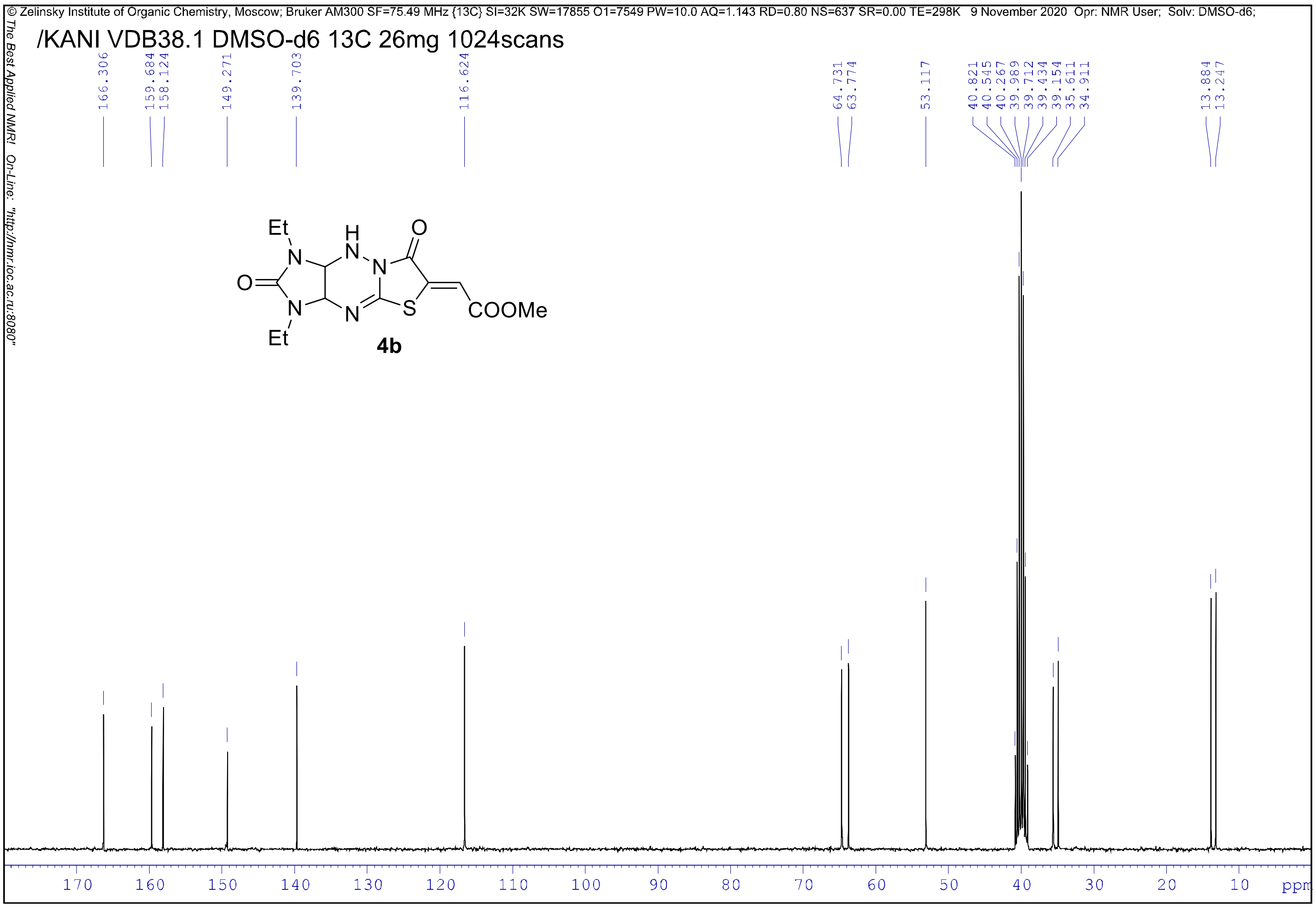

^{1}H NMR spectrum of **4c**

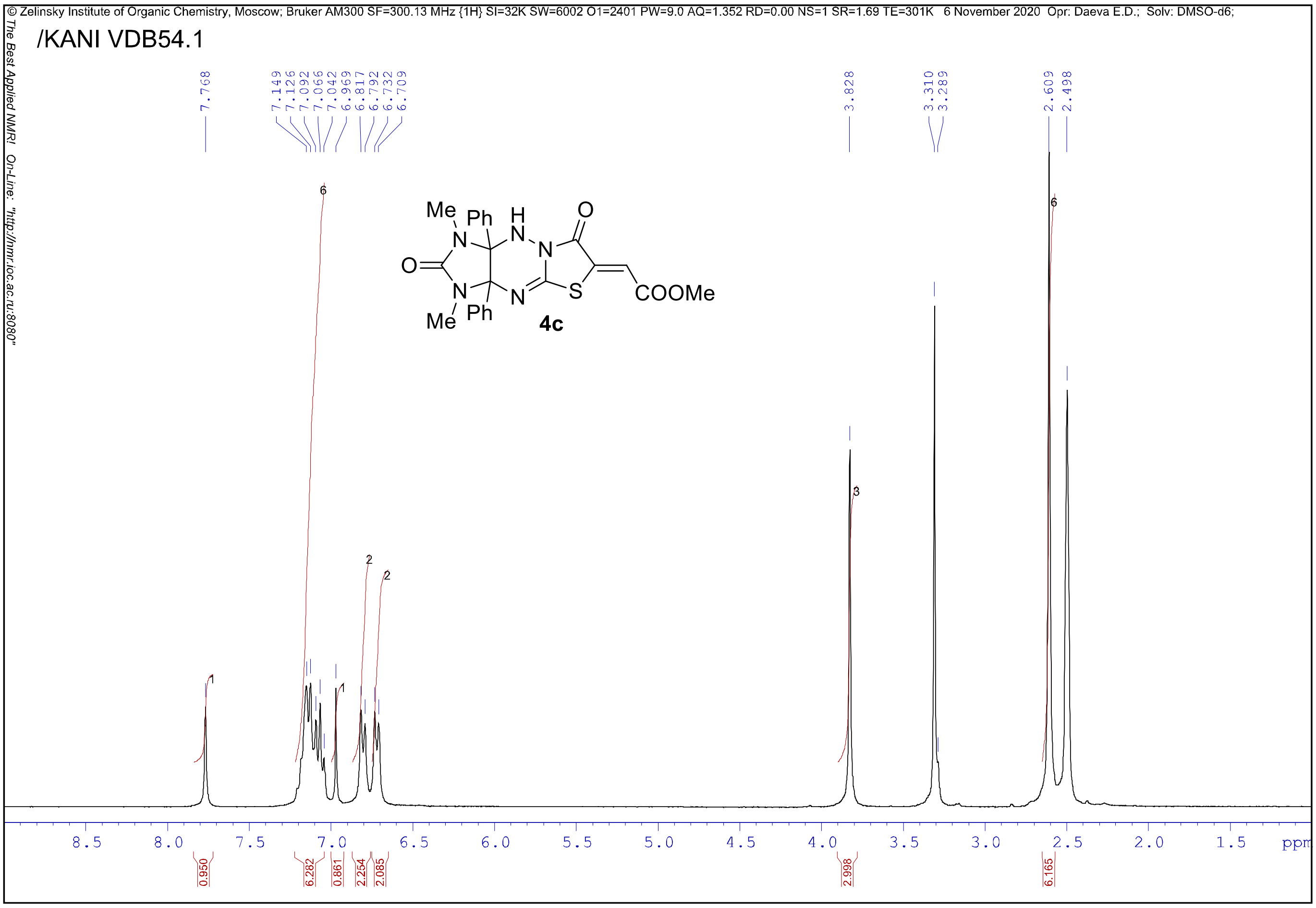

^{13}C NMR spectrum of **4c**

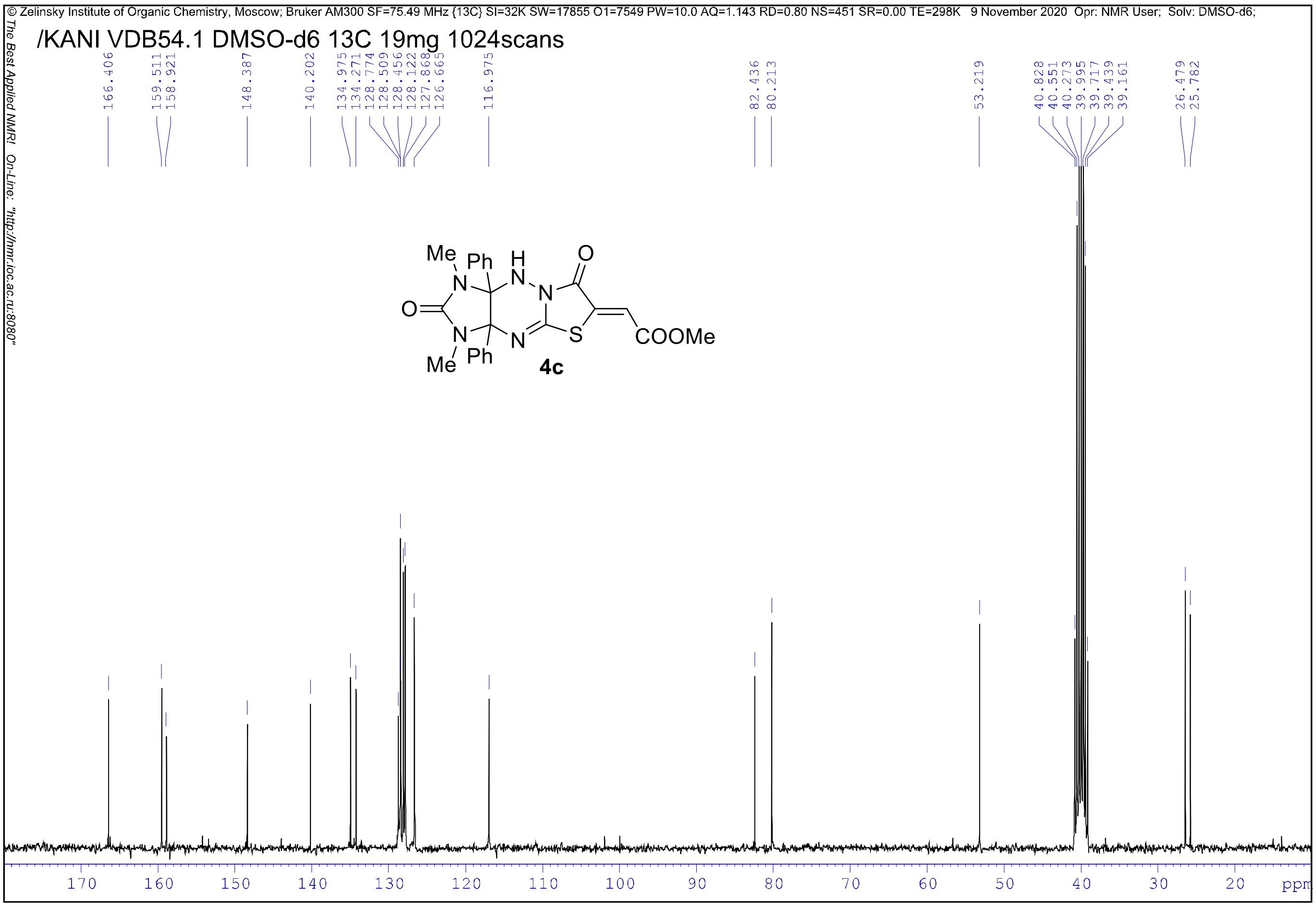

^{1}H NMR spectrum of **4d**

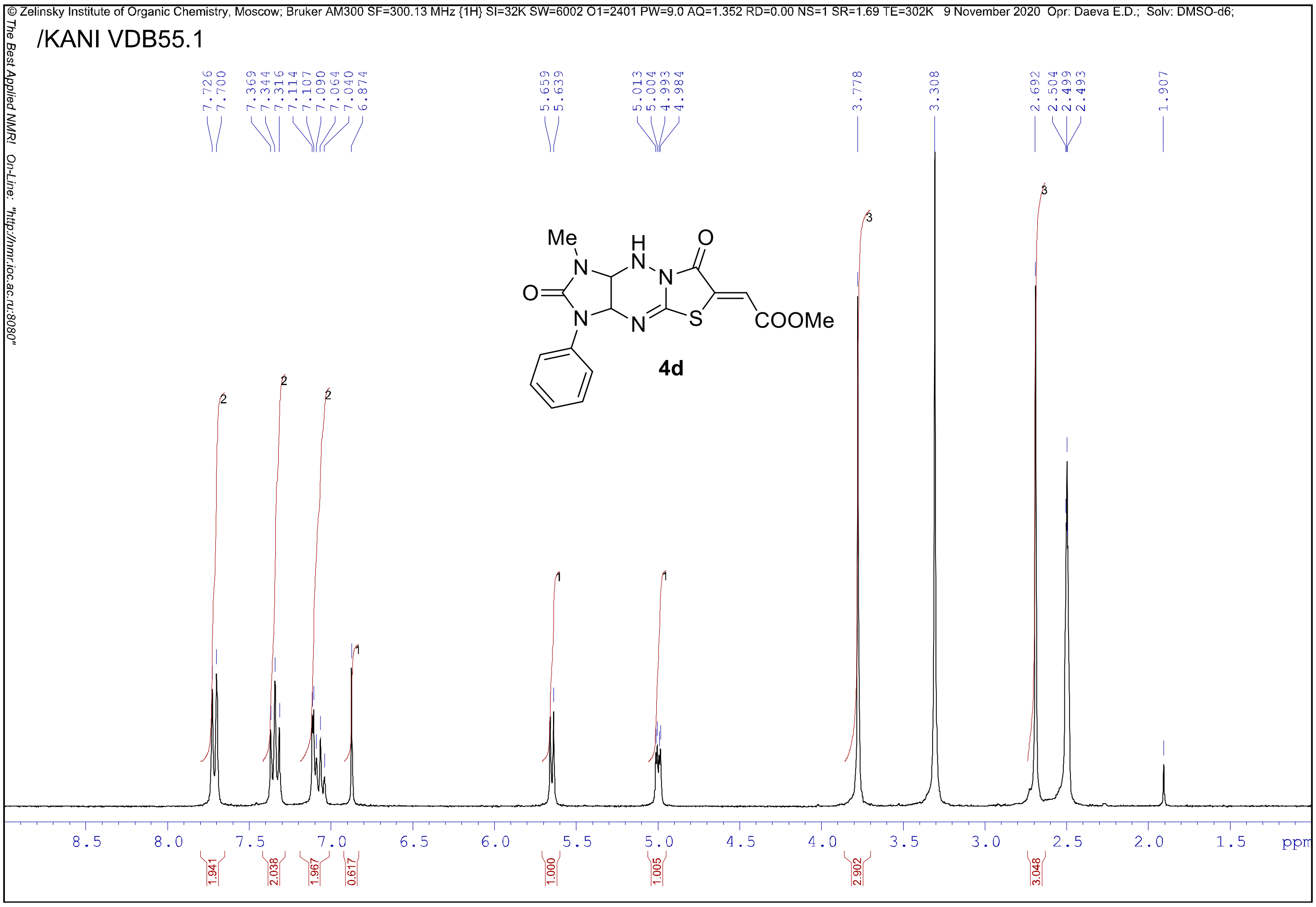

^{13}C NMR spectrum of **4d**

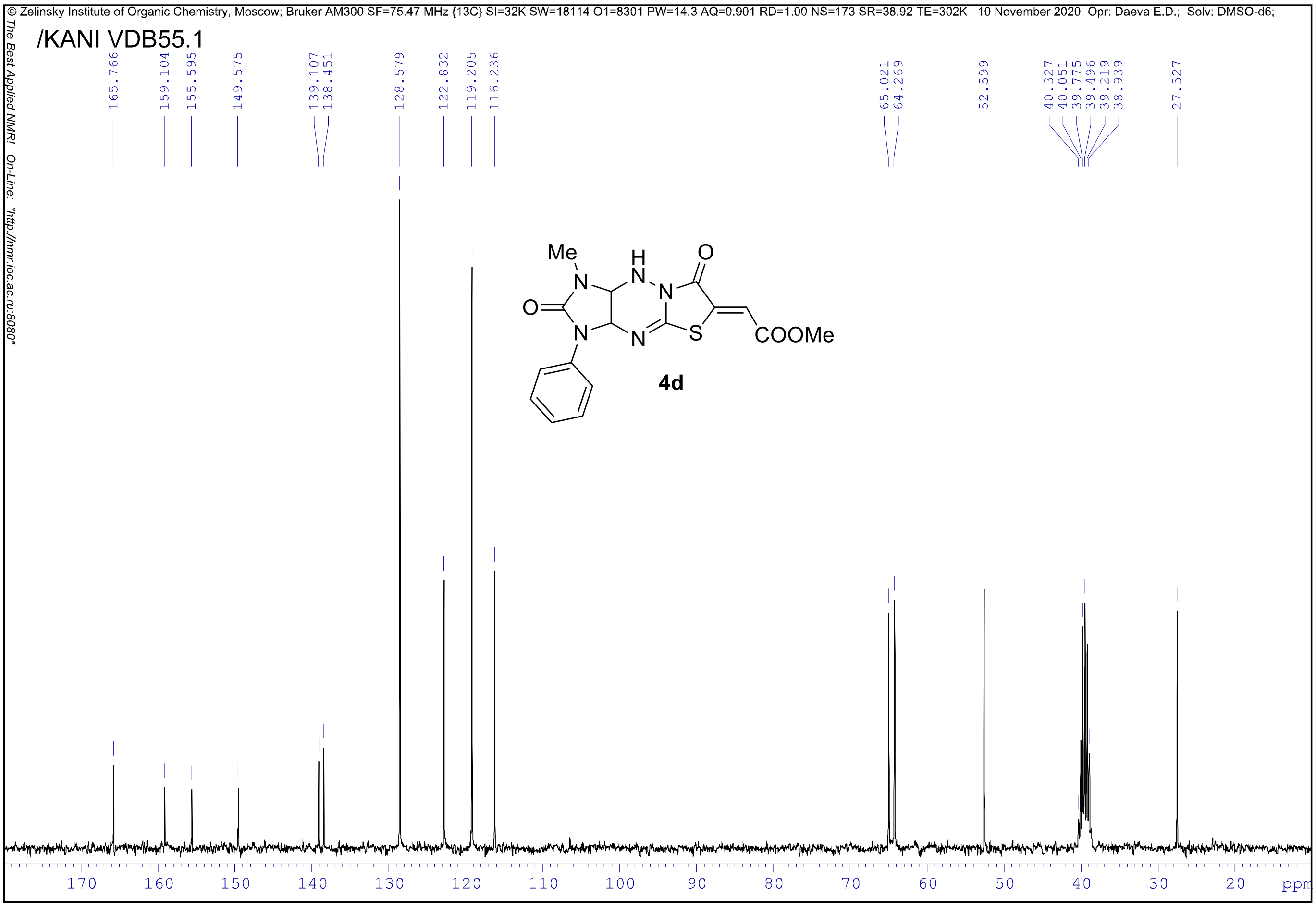

^{1}H NMR spectrum of **4e**

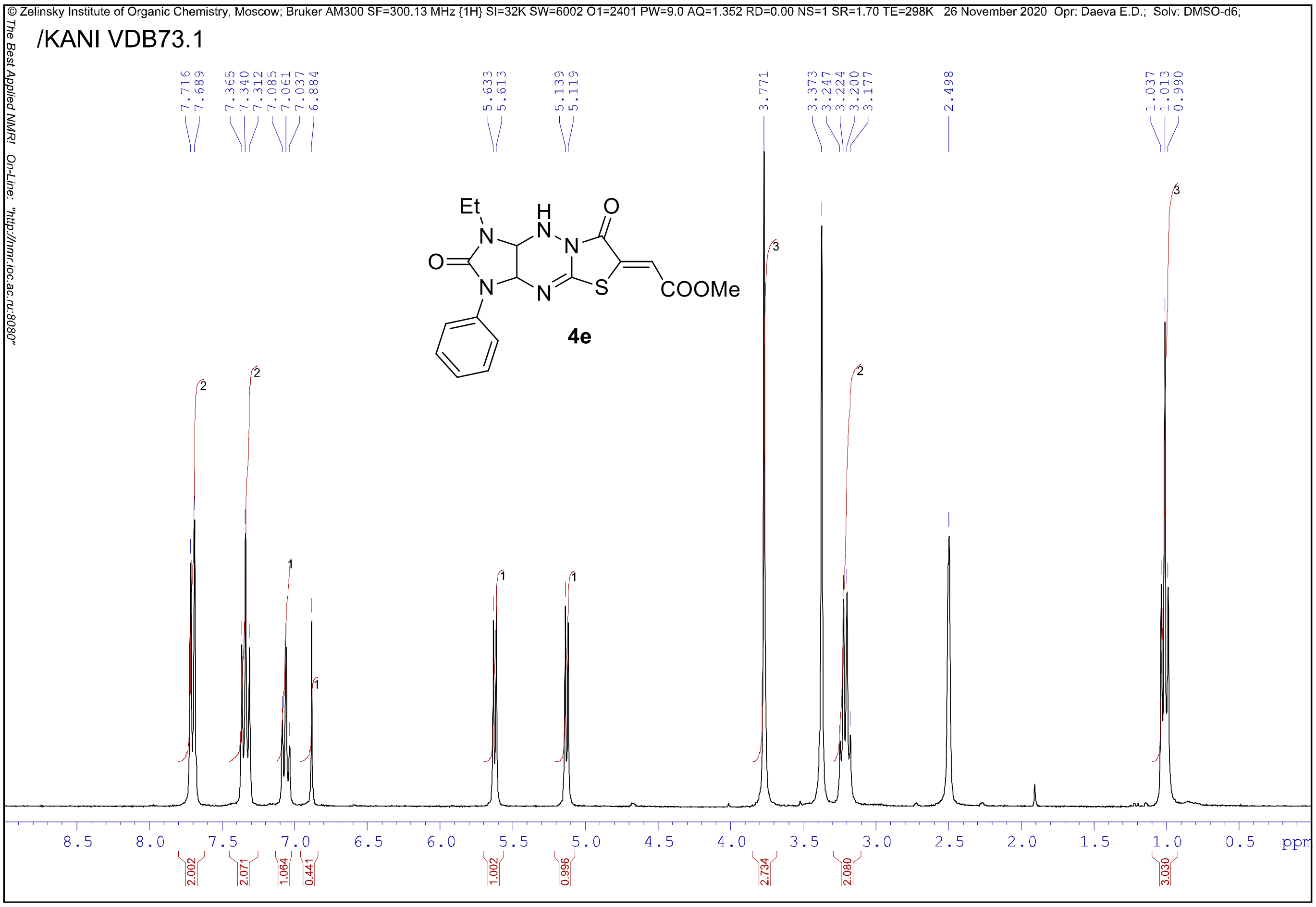

^{13}C NMR spectrum of **4e**

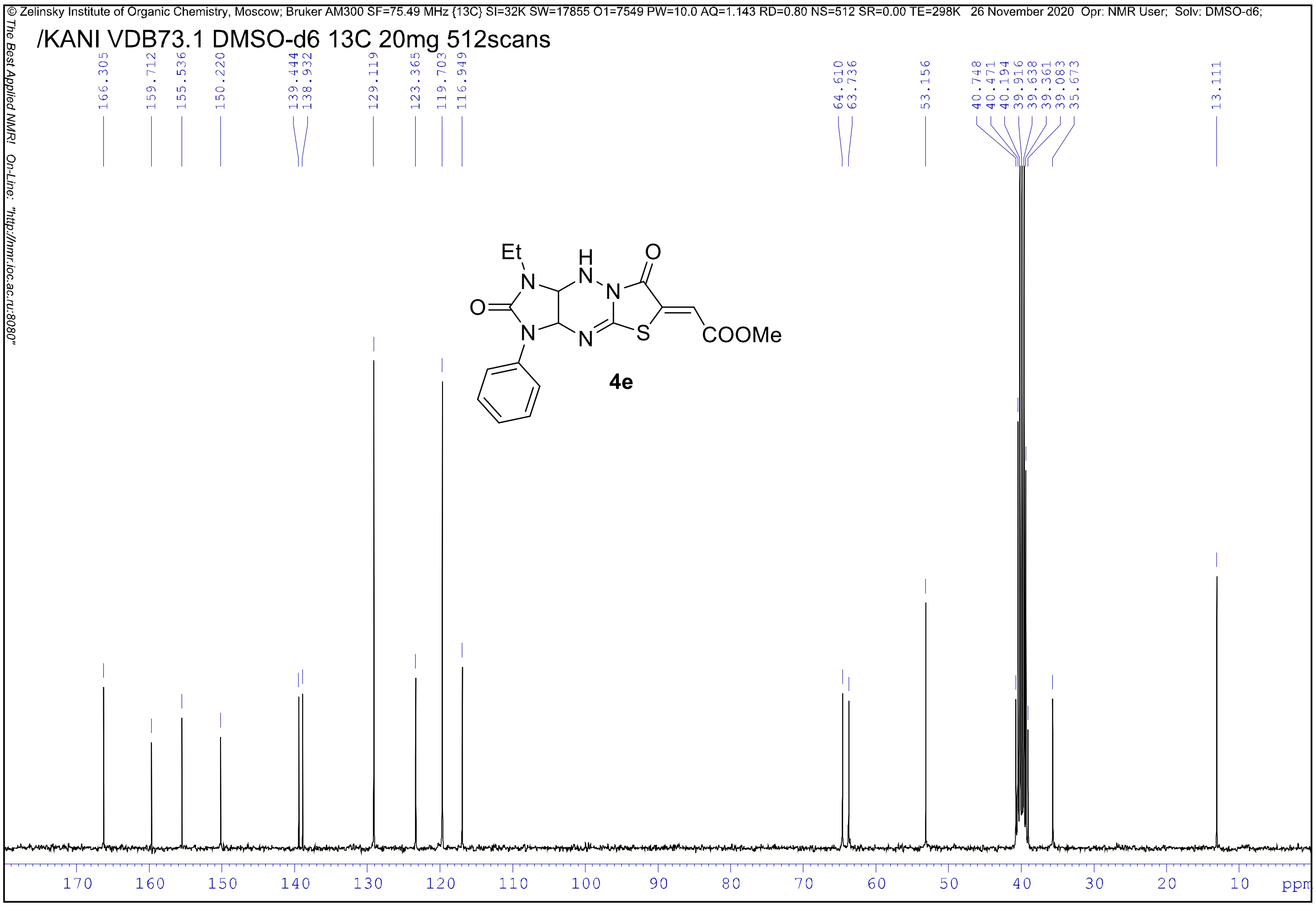

^{1}H NMR spectrum of **4f**

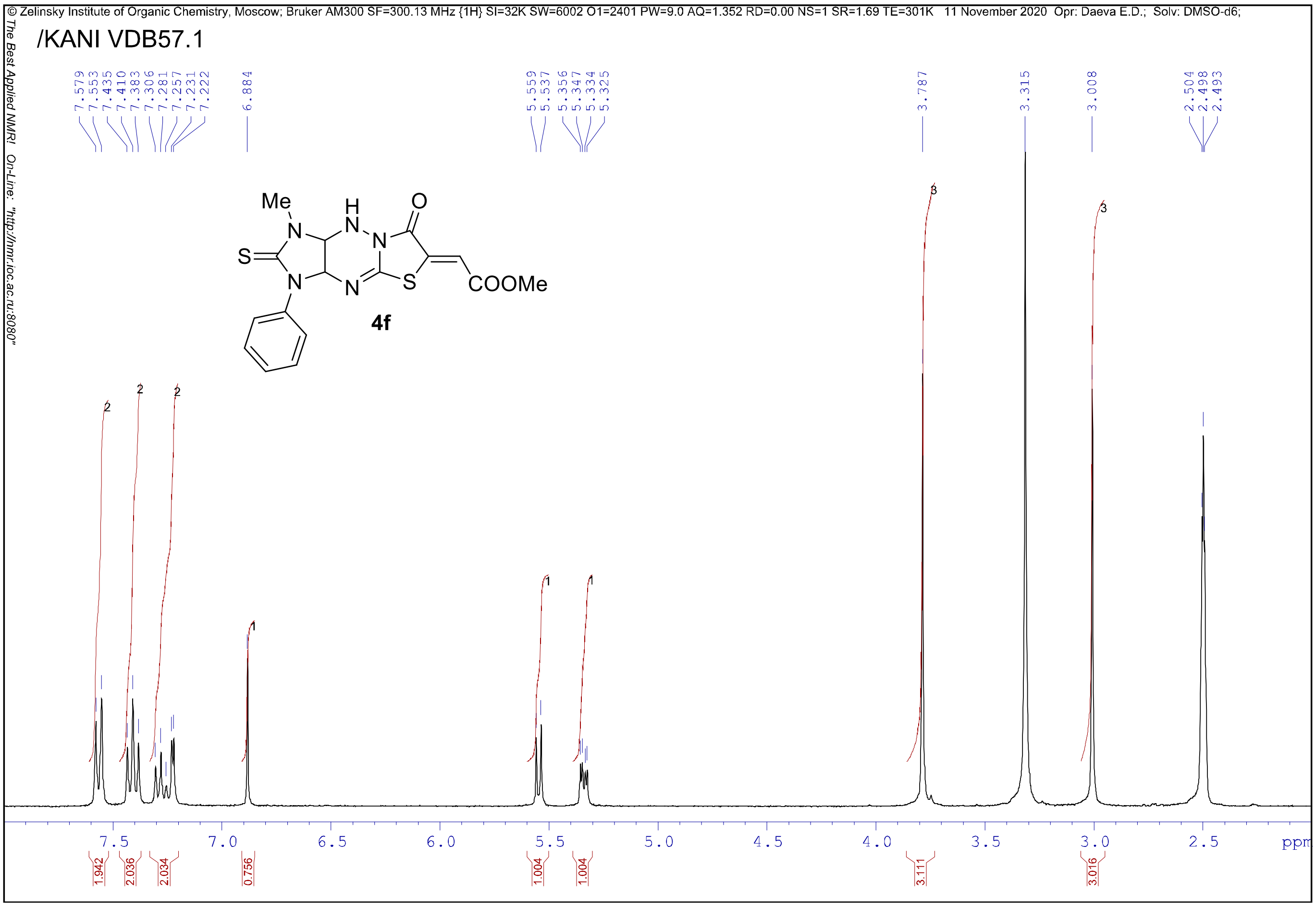

^{13}C NMR spectrum of **4f**

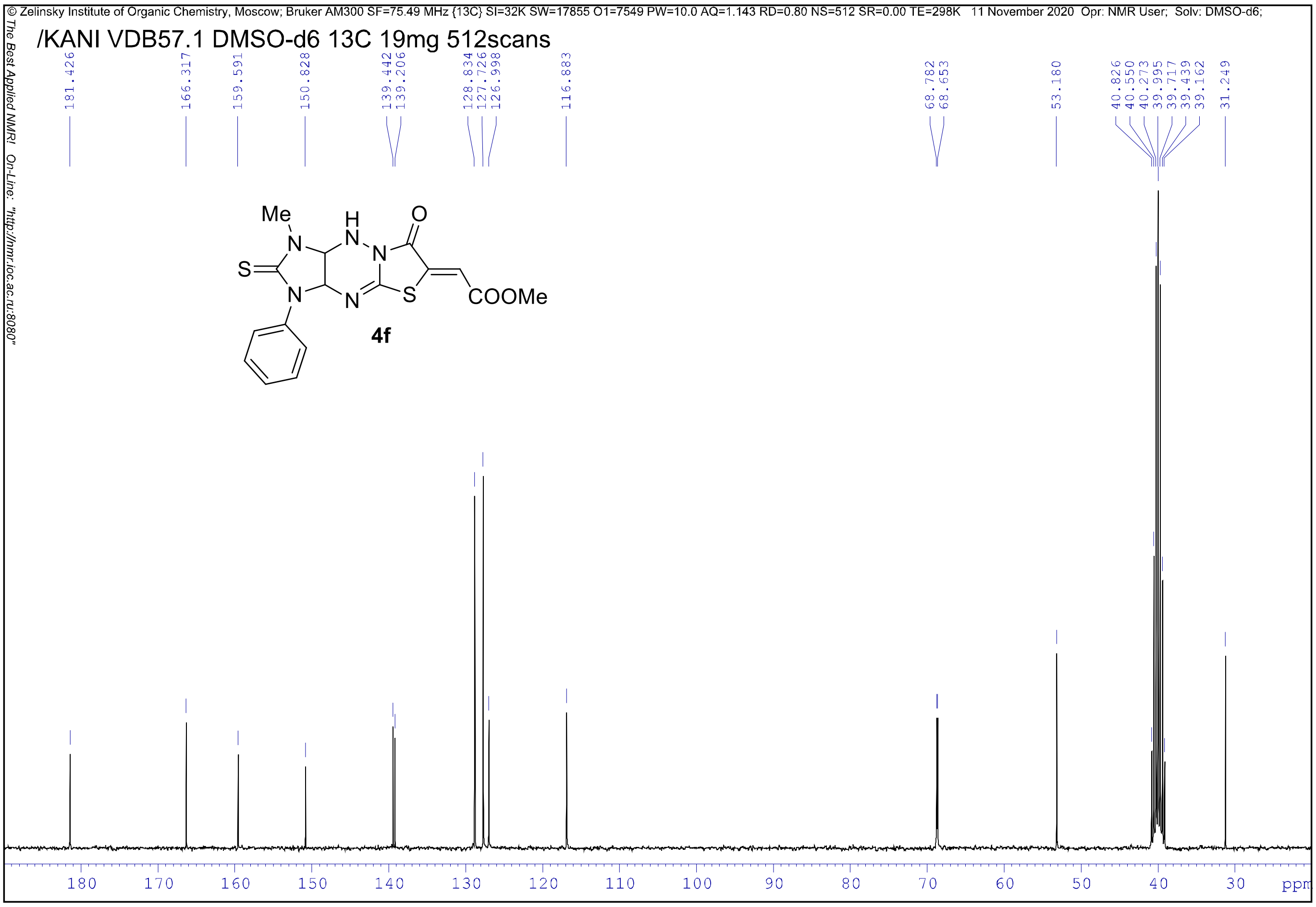

^{1}H NMR spectrum of **4g**

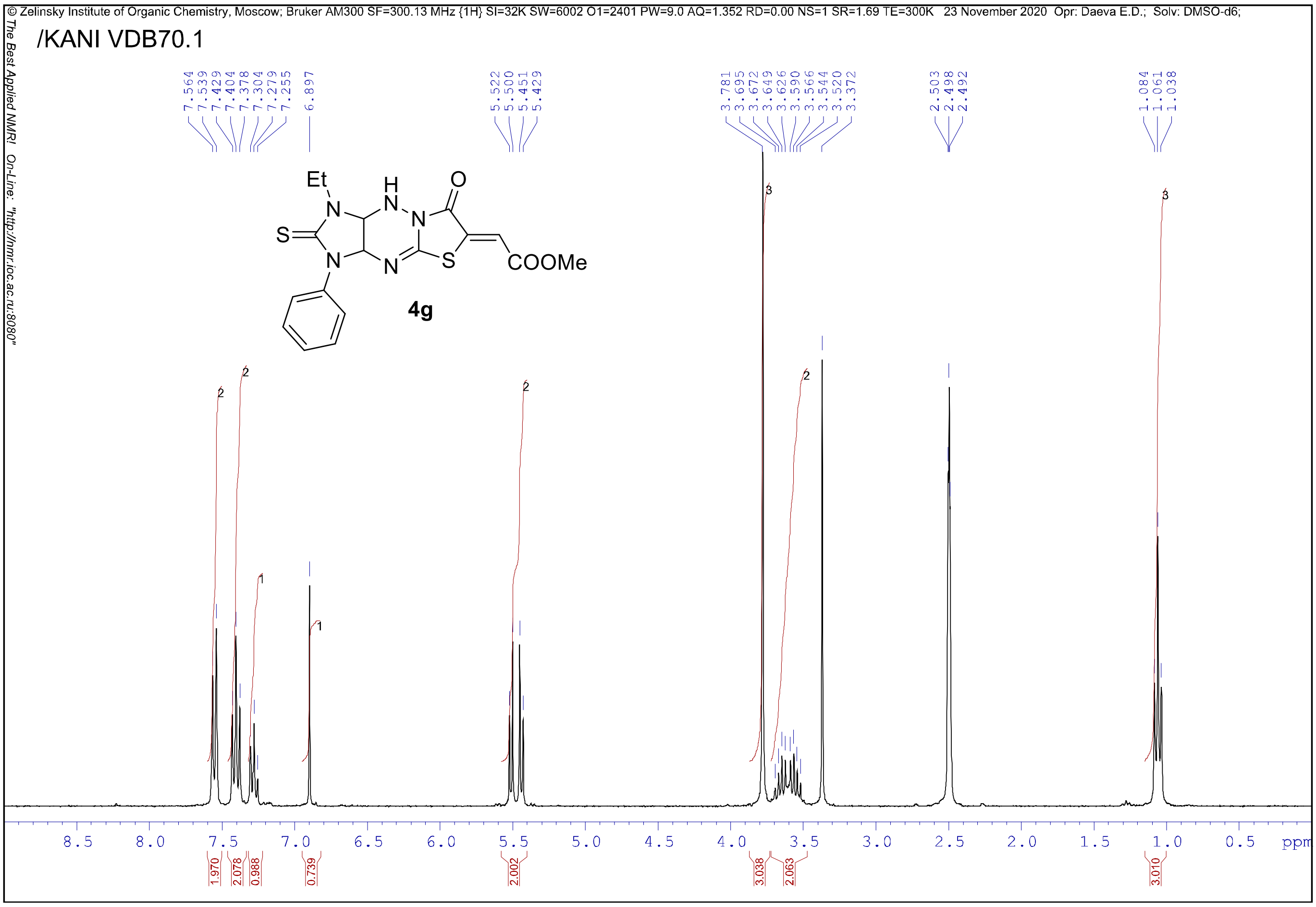

^{13}C NMR spectrum of **4g**

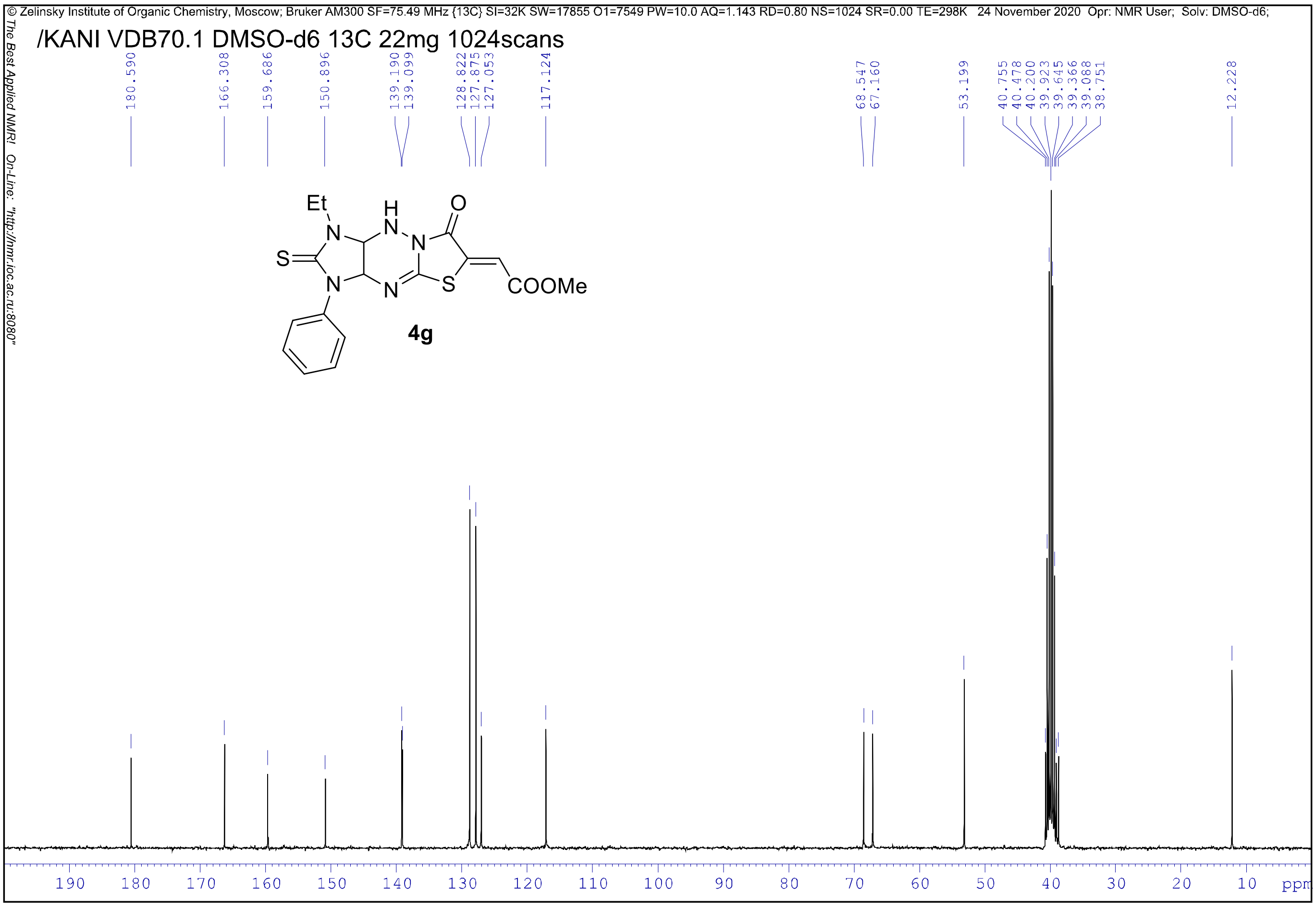

^{1}H NMR spectrum of **4h**

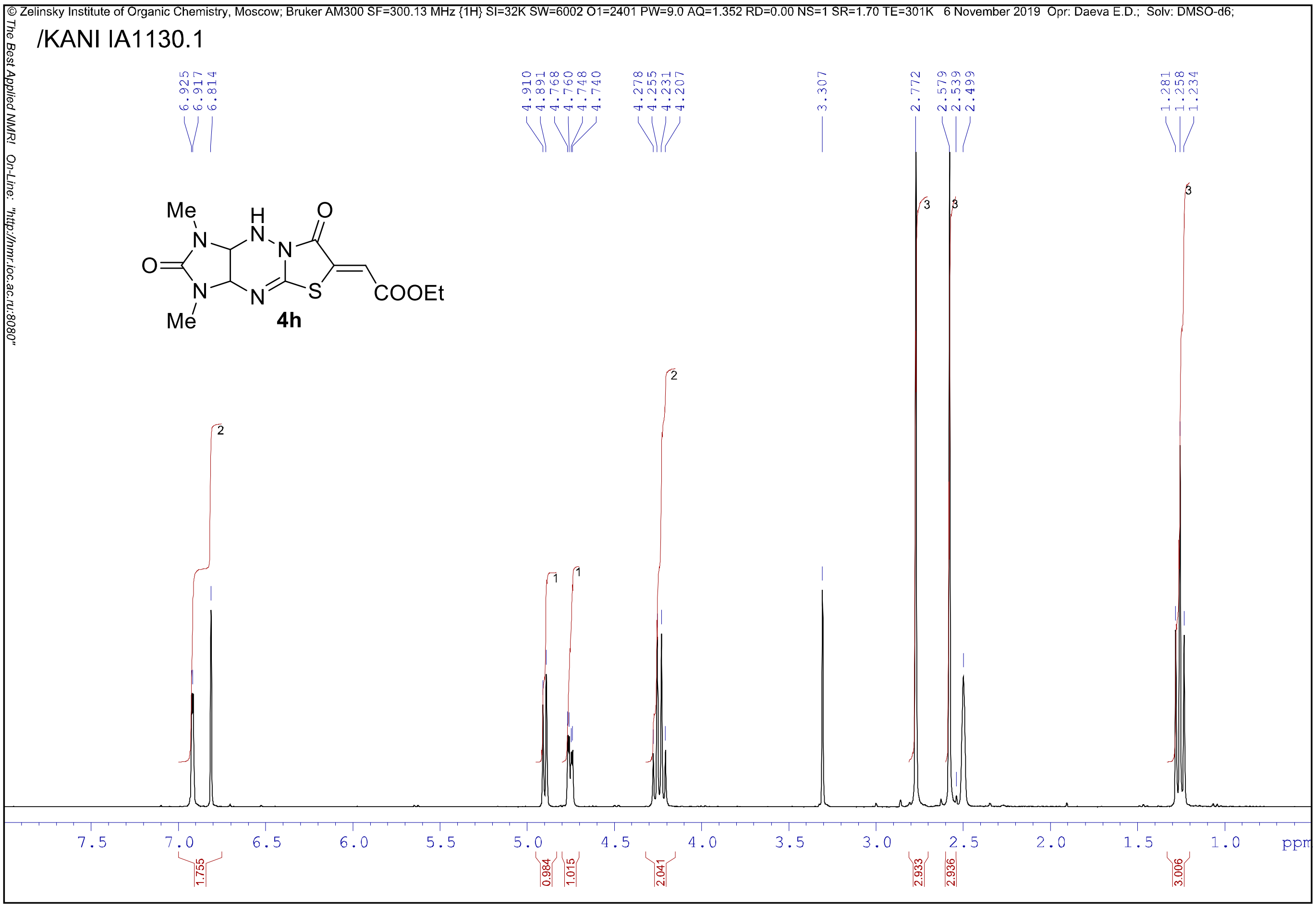

^{13}C NMR spectrum of **4h**

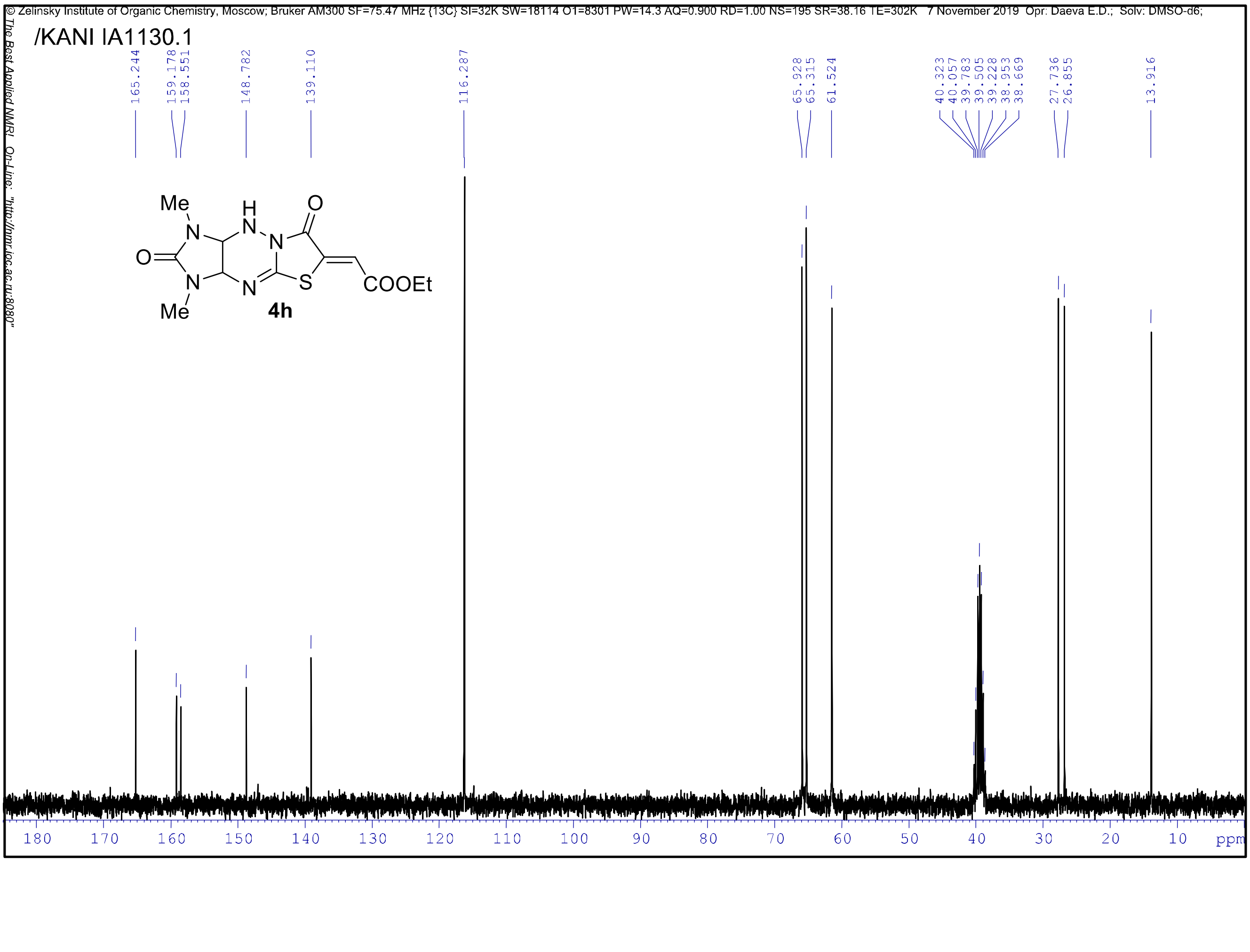

^{1}H NMR spectrum of **4i**

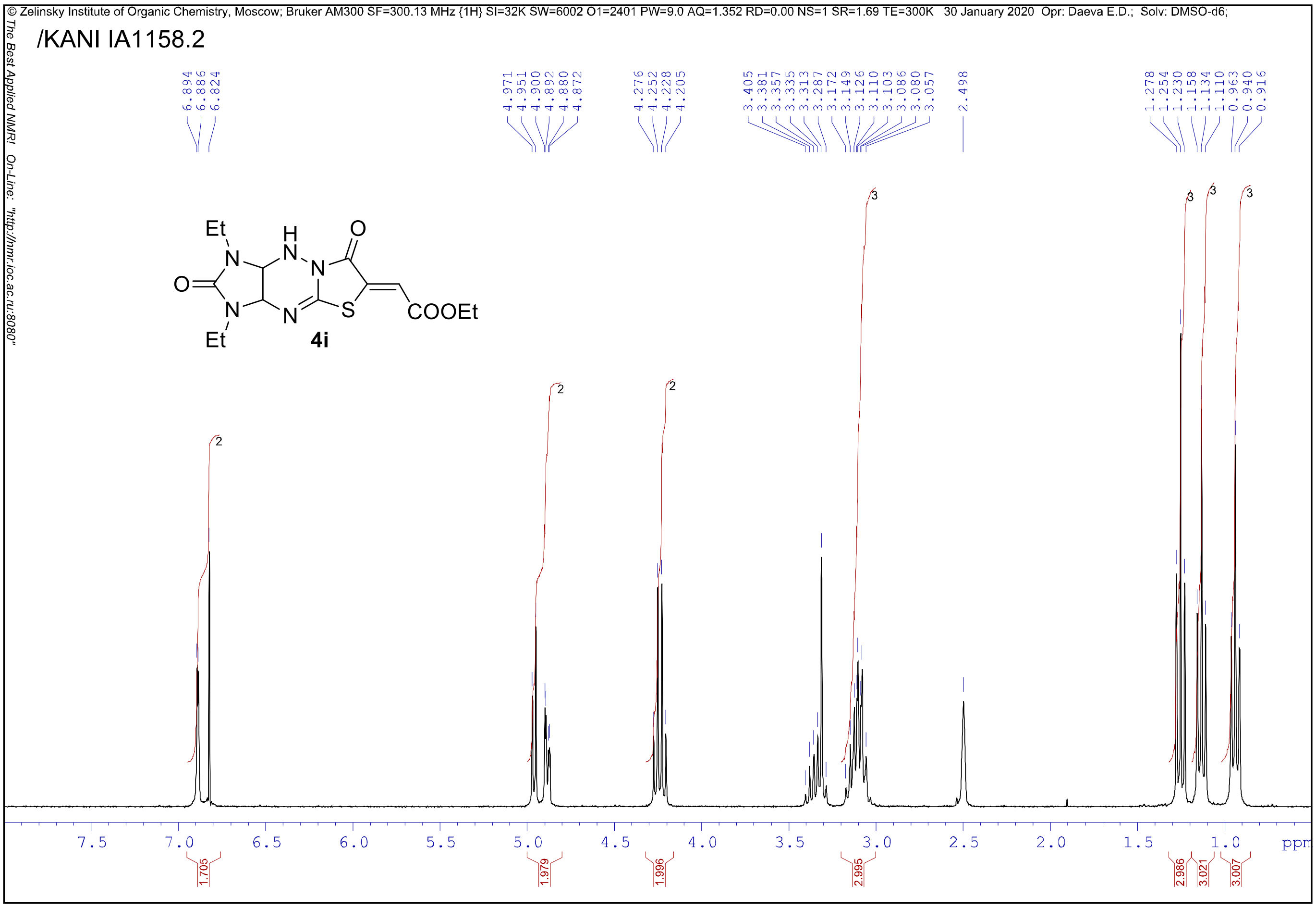

^{13}C NMR spectrum of **4i**

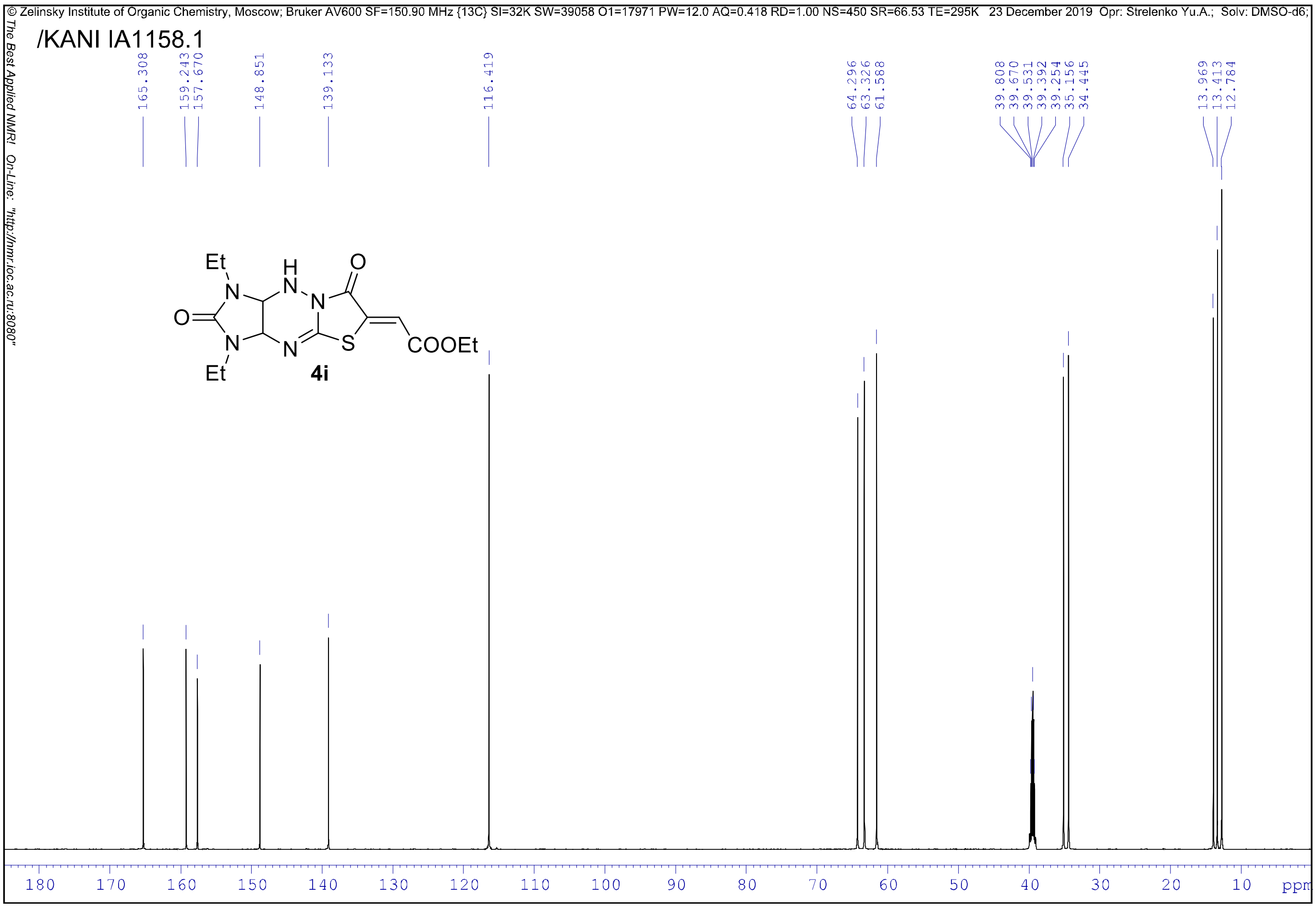

^{1}H NMR spectrum of **4j**

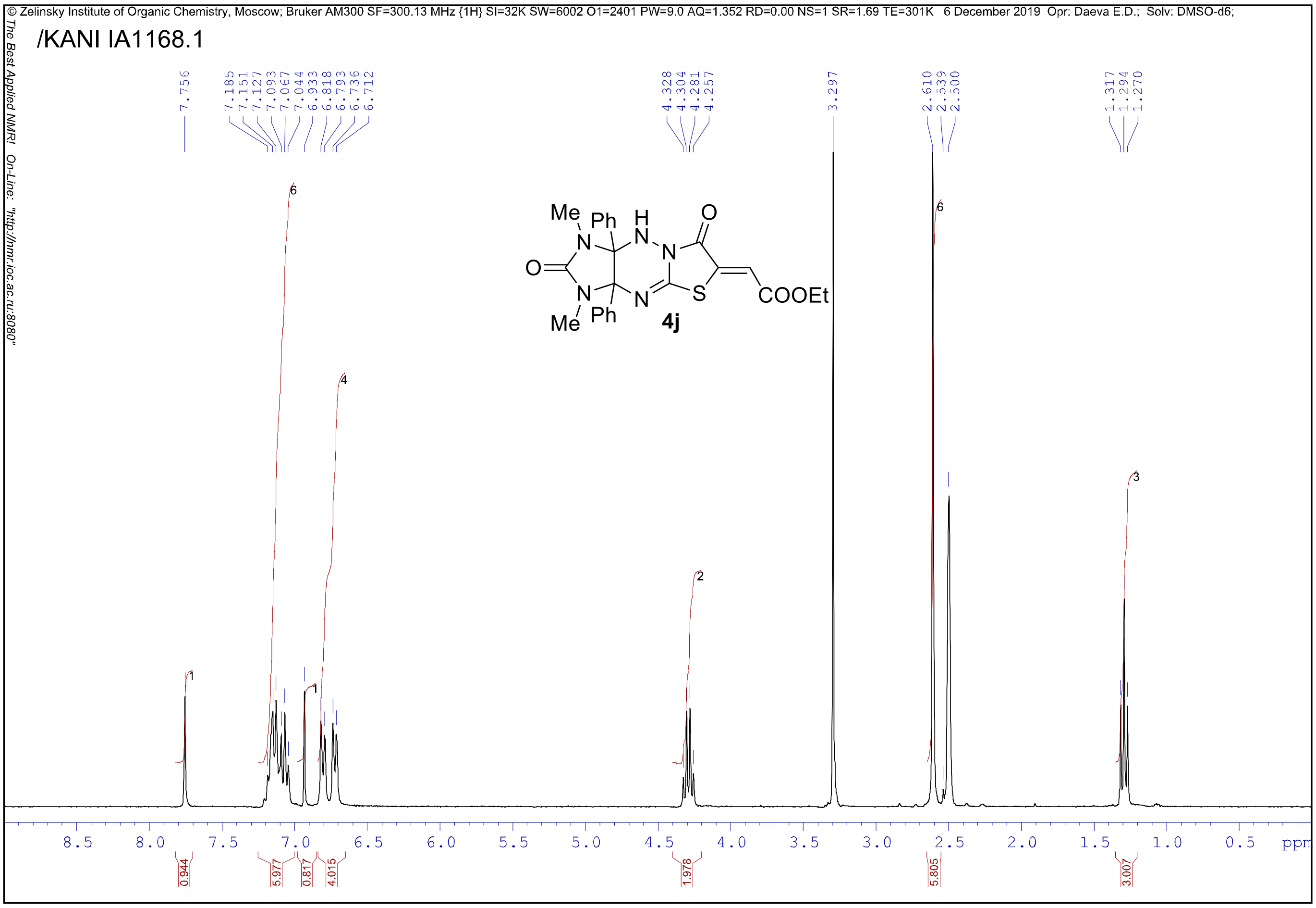

^{13}C NMR spectrum of **4j**

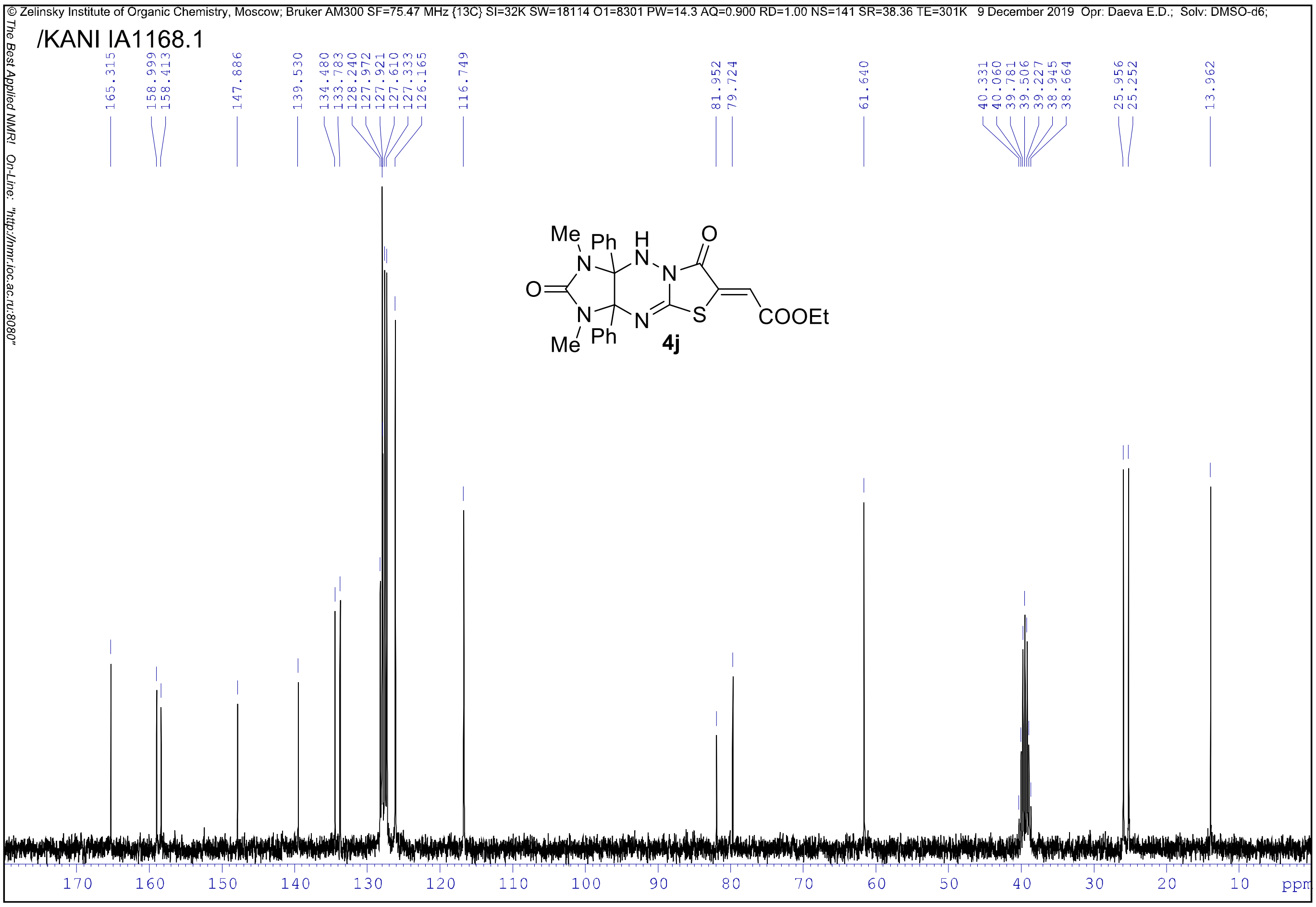

^{1}H NMR spectrum of **4k**

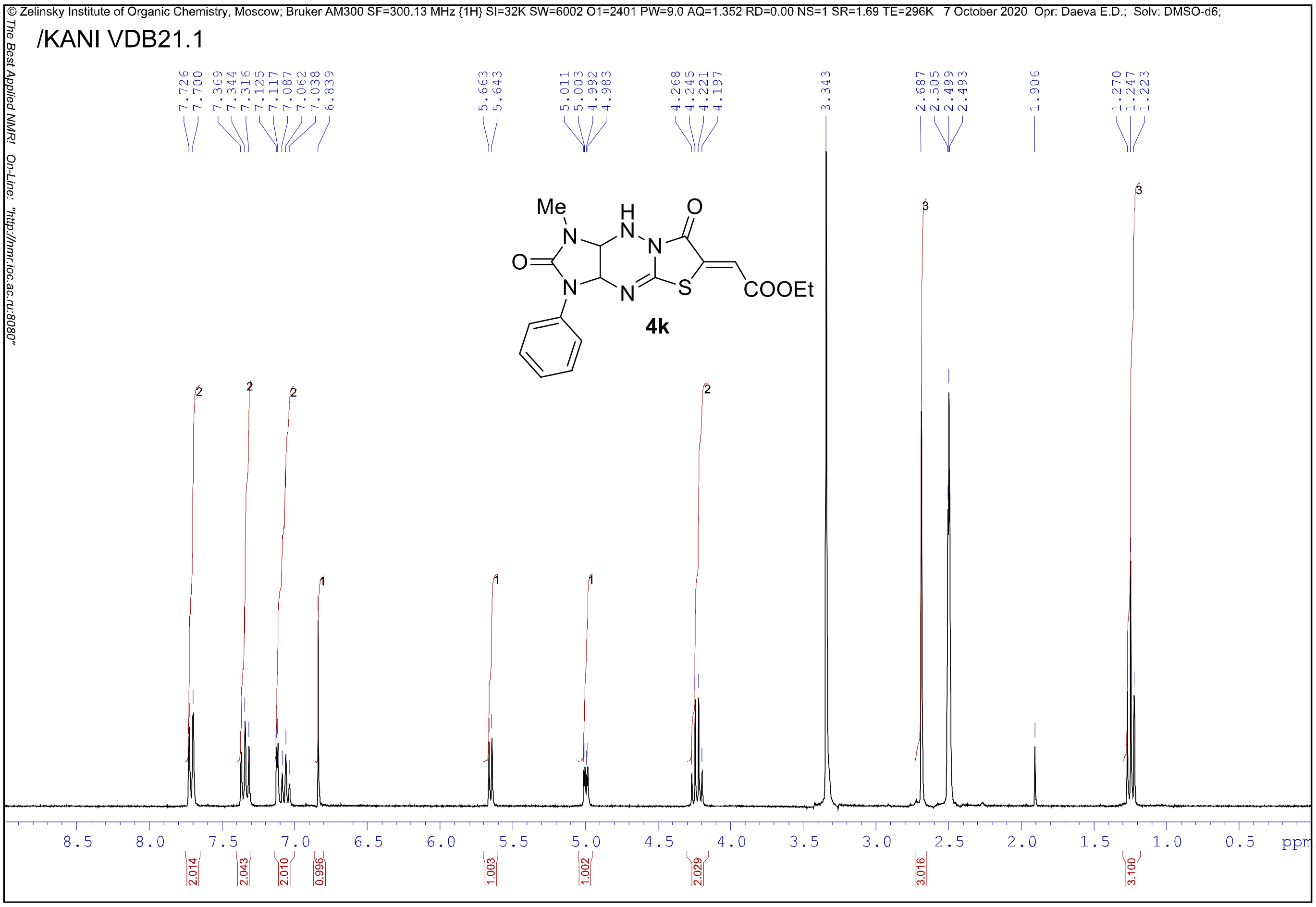

^{13}C NMR spectrum of **4k**

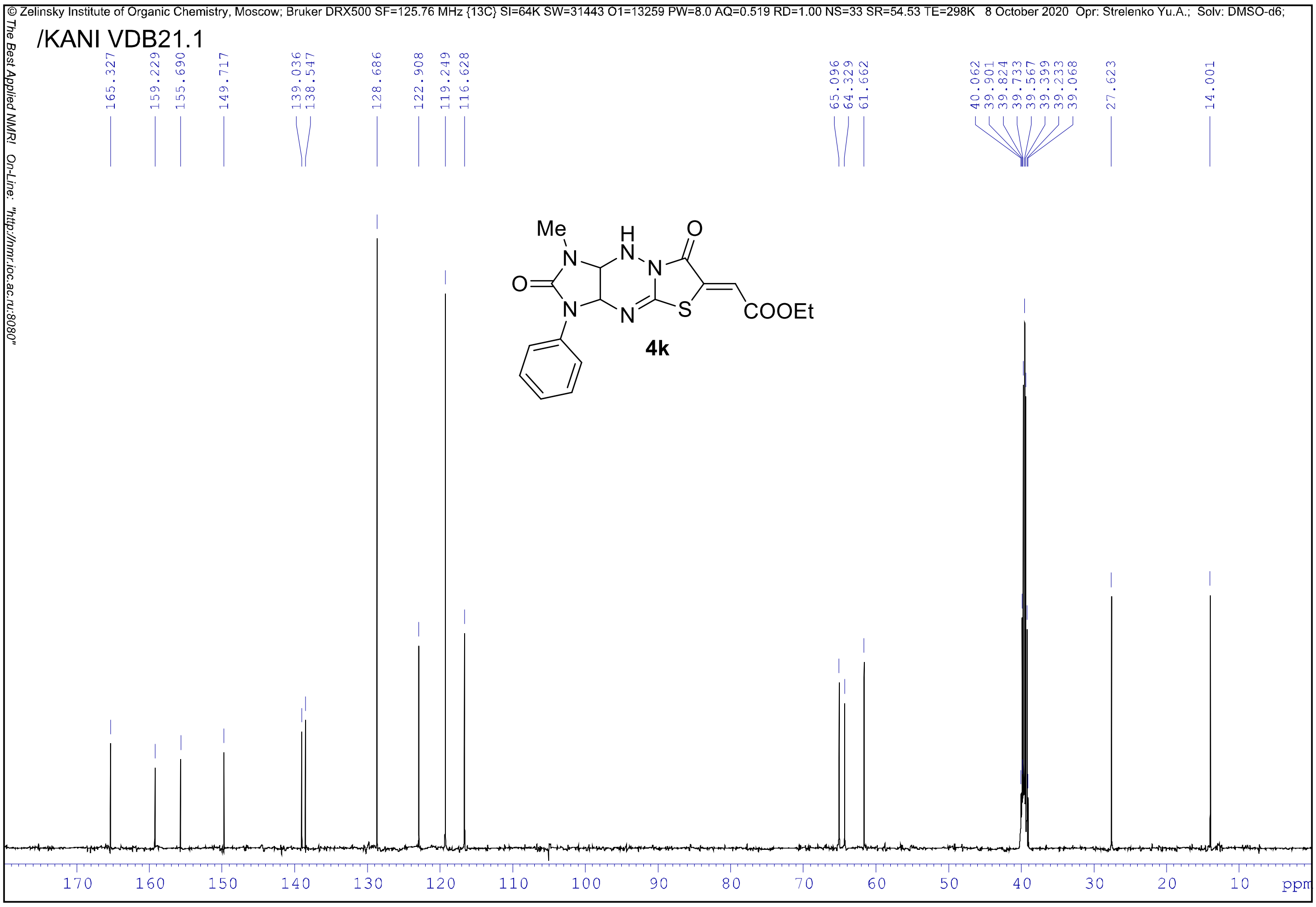

^{1}H NMR spectrum of **4l**

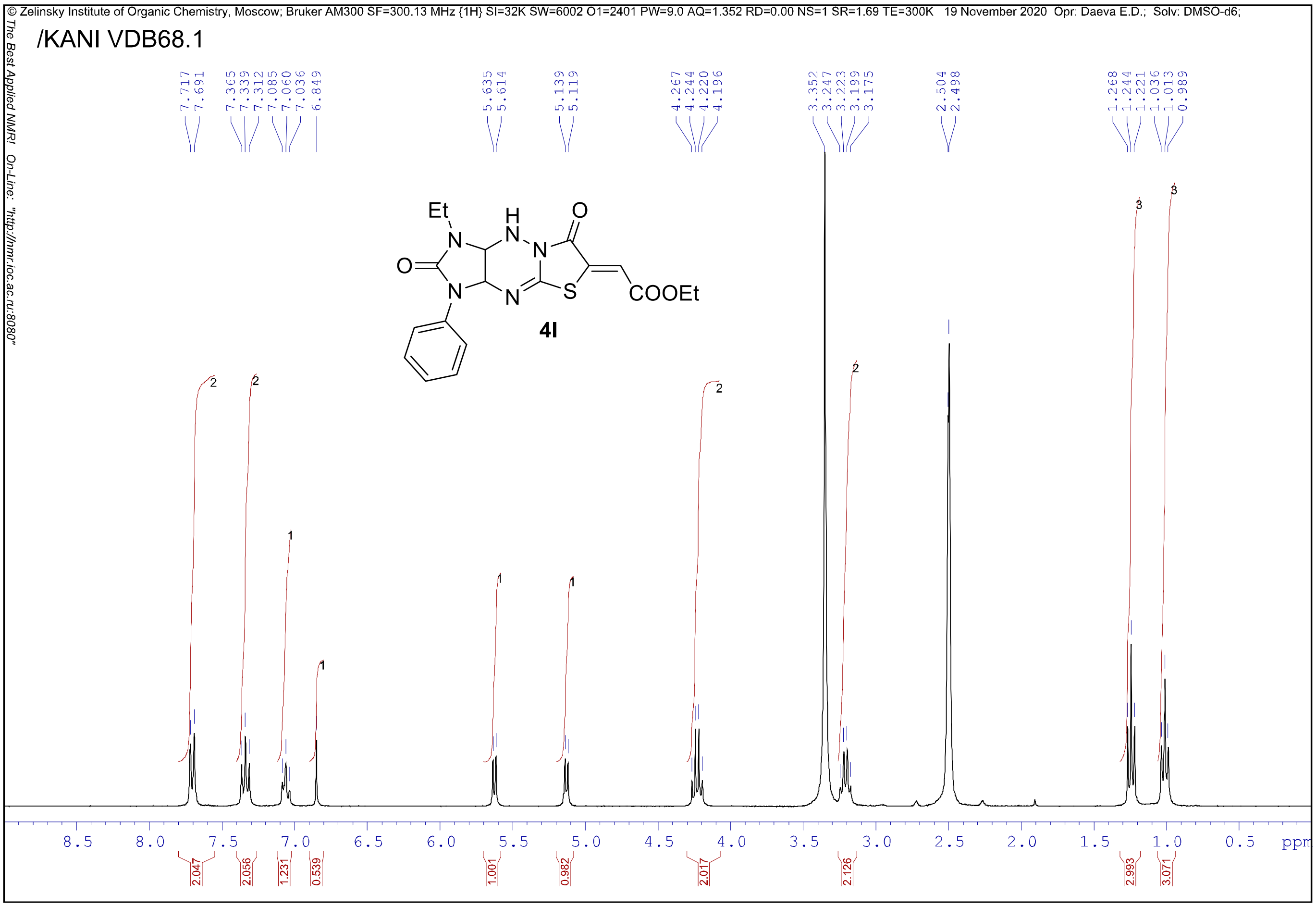

^{13}C NMR spectrum of **4l**

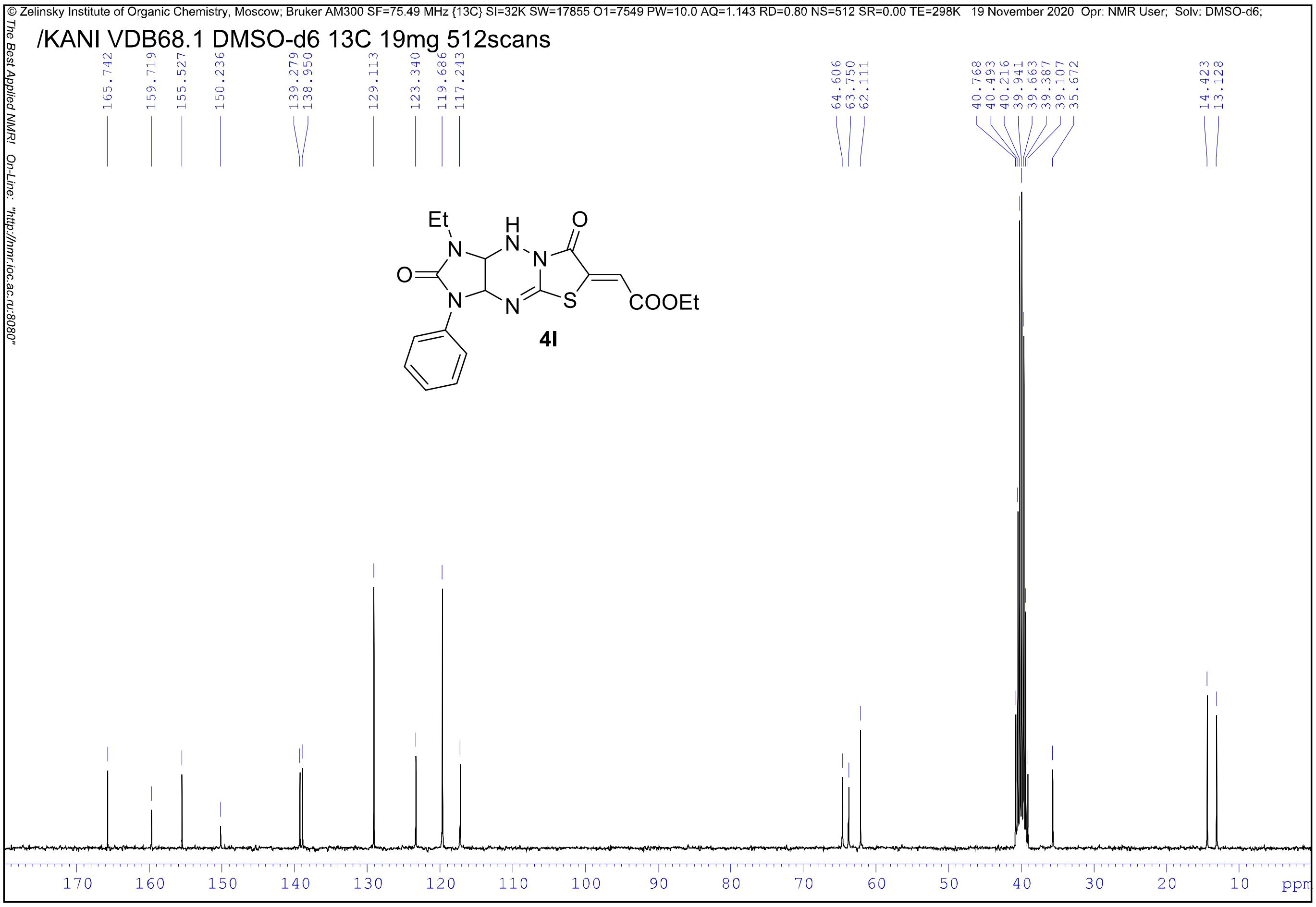

^{1}H NMR spectrum of **4m**

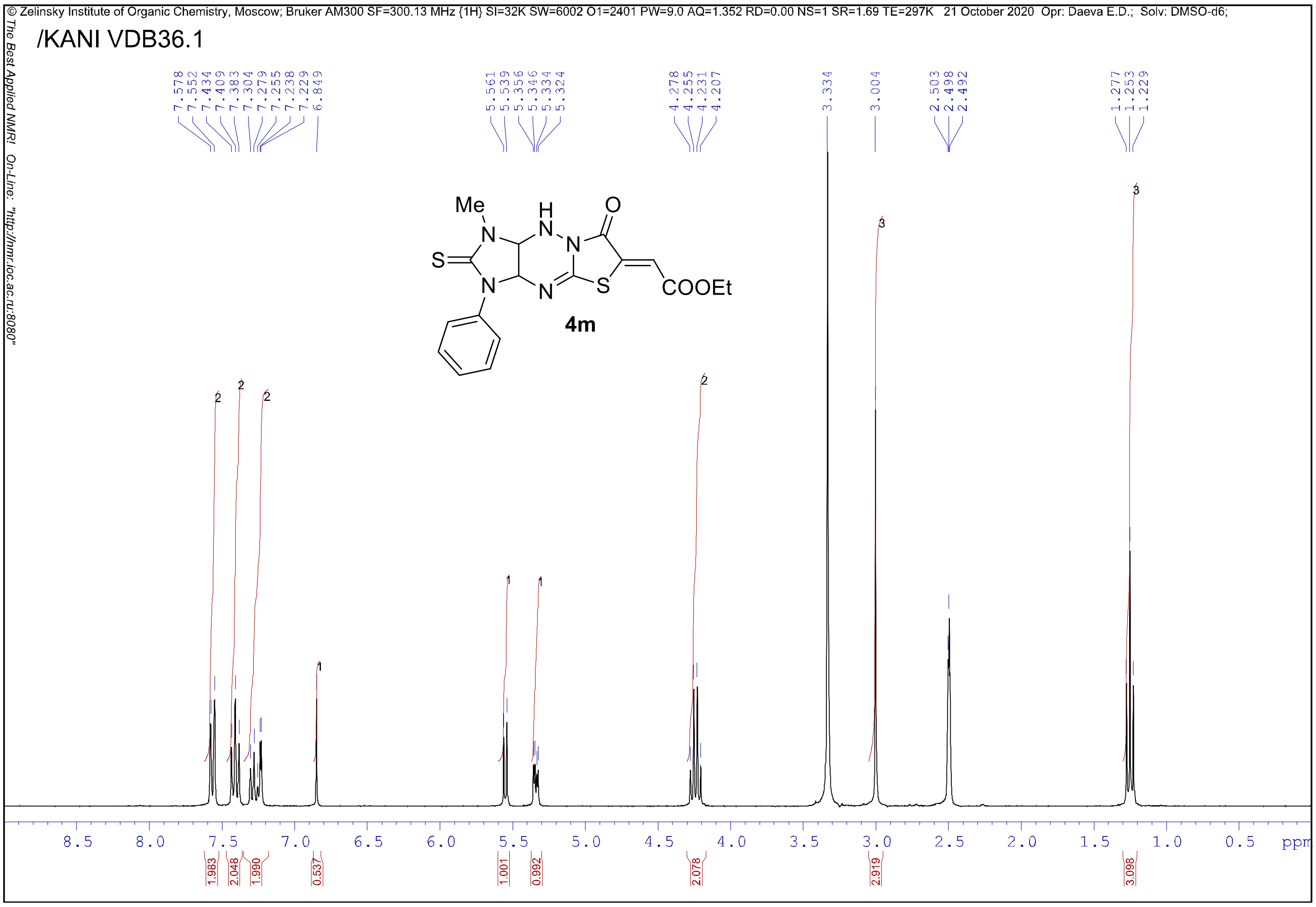

^{13}C NMR spectrum of **4m**

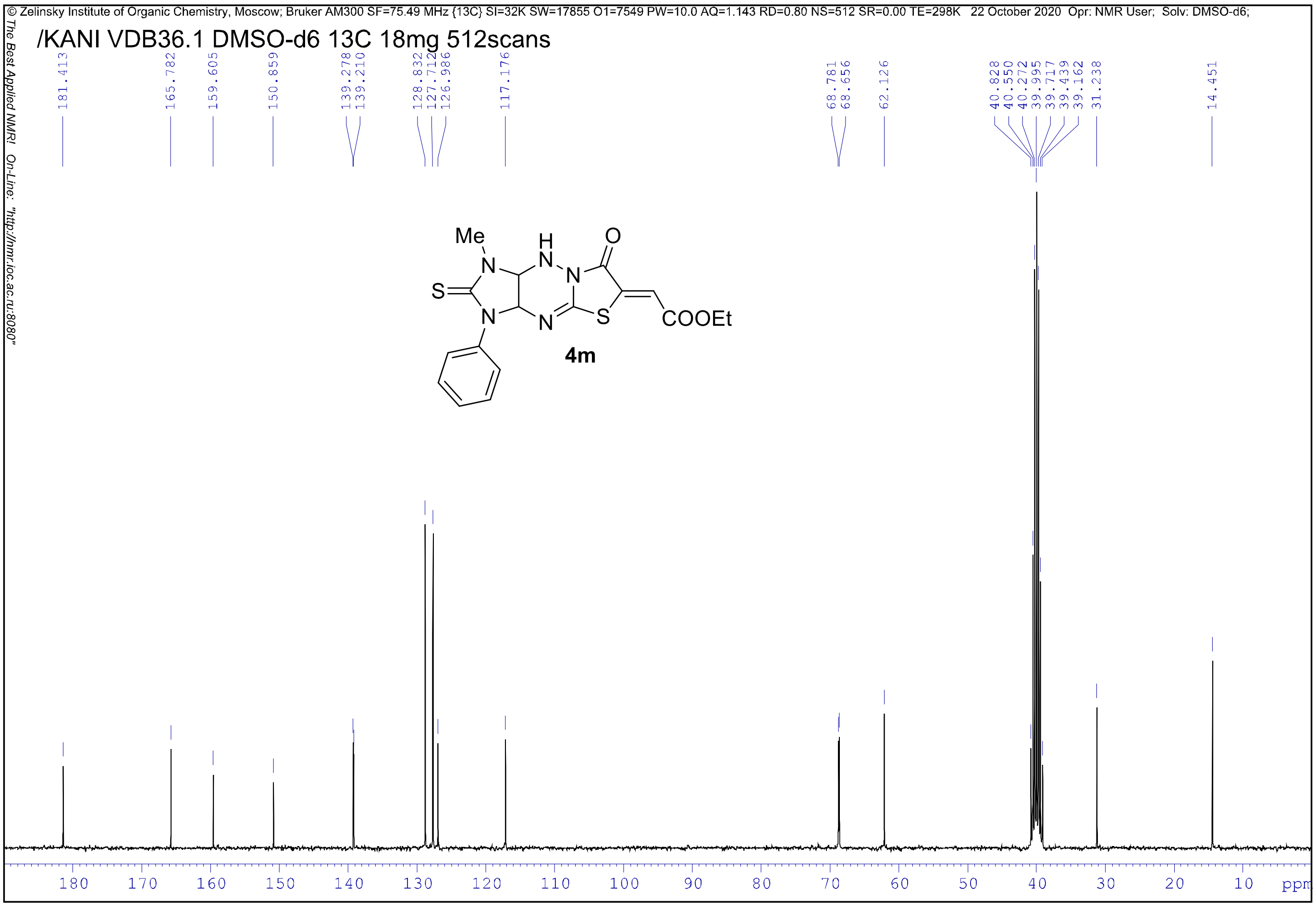

^{1}H NMR spectrum of **4n**

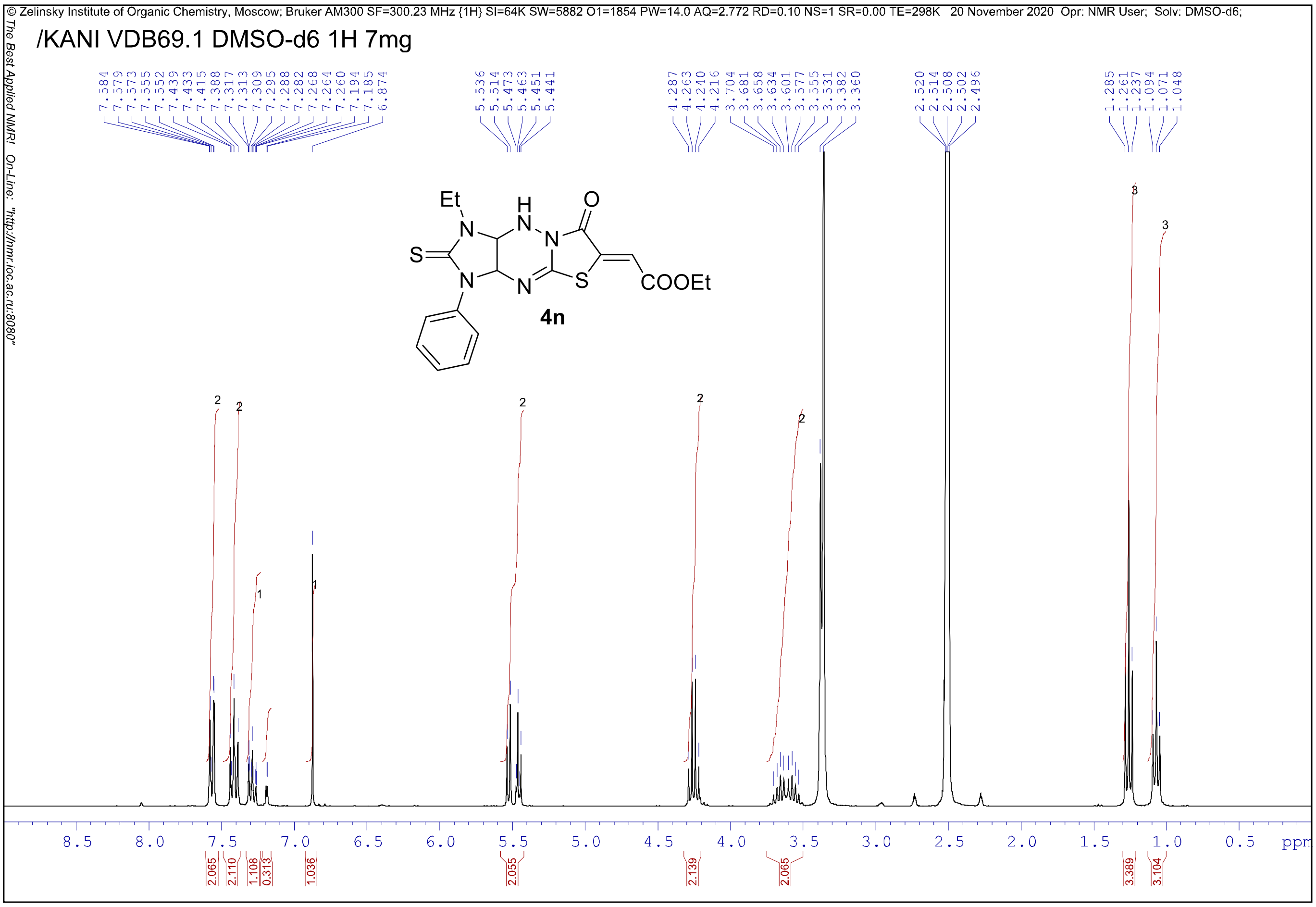

^{13}C NMR spectrum of **4n**

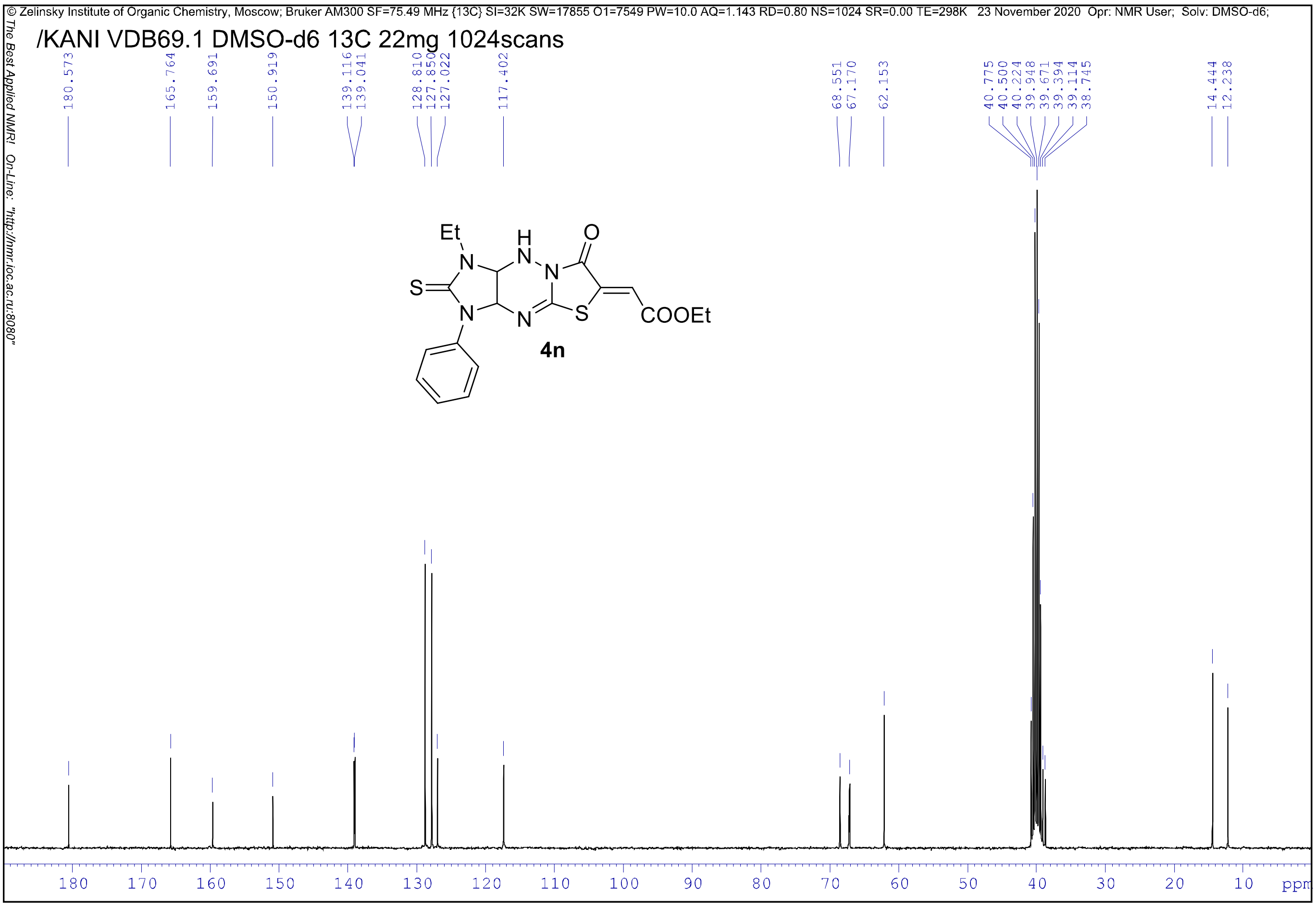

^{1}H NMR spectrum of **5a**

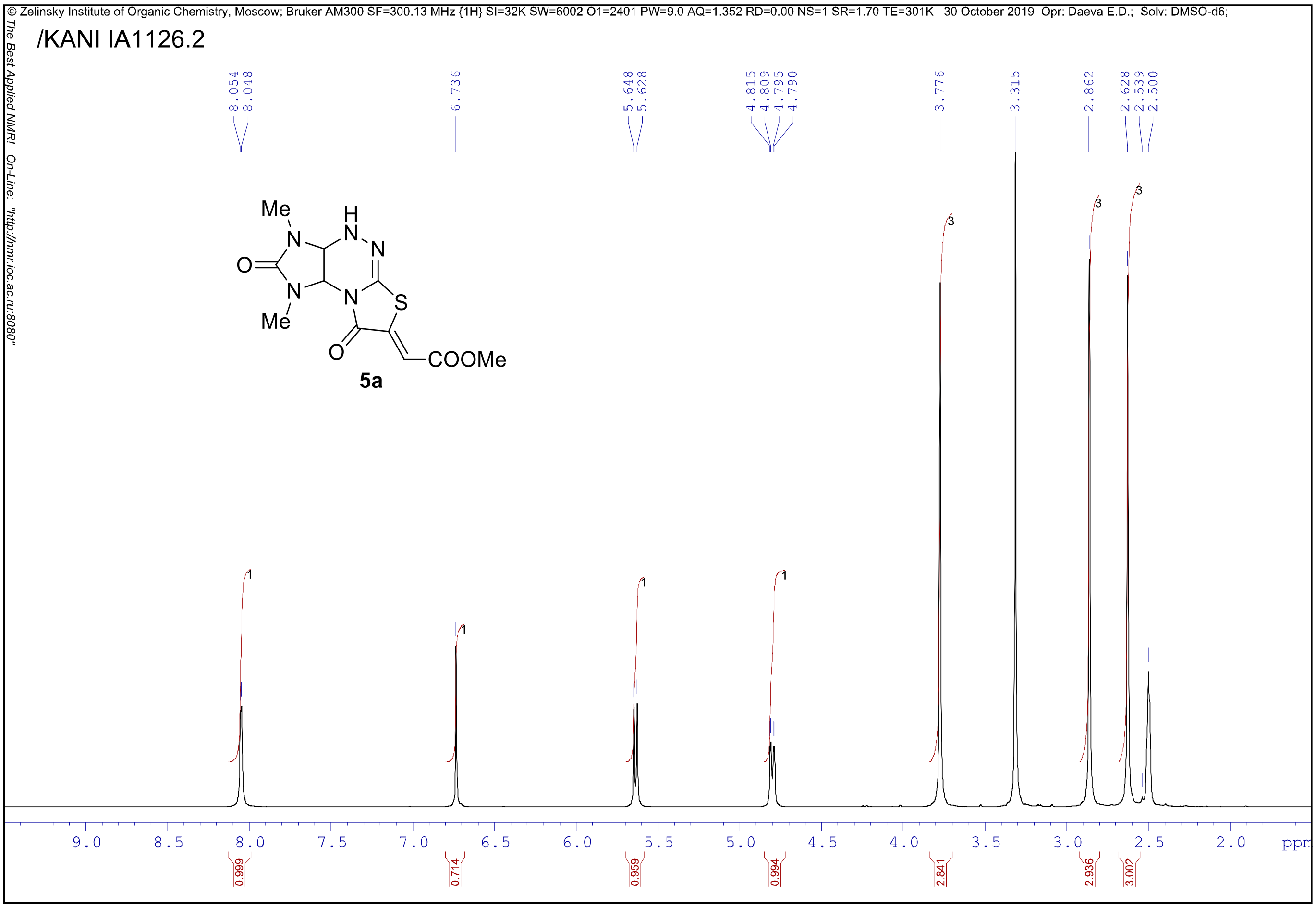

^{13}C NMR spectrum of **5a**

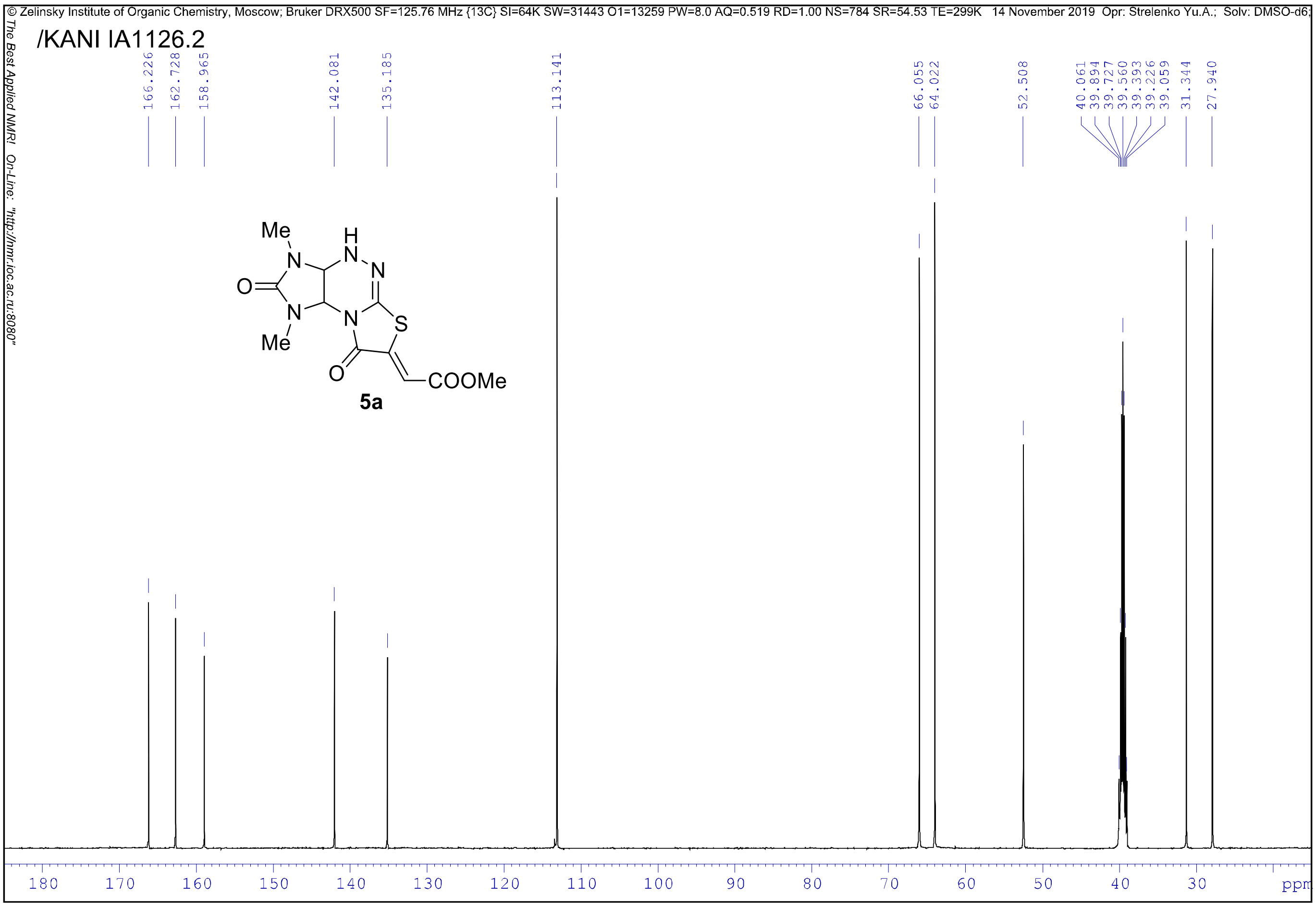

^{1}H NMR spectrum of **5b**

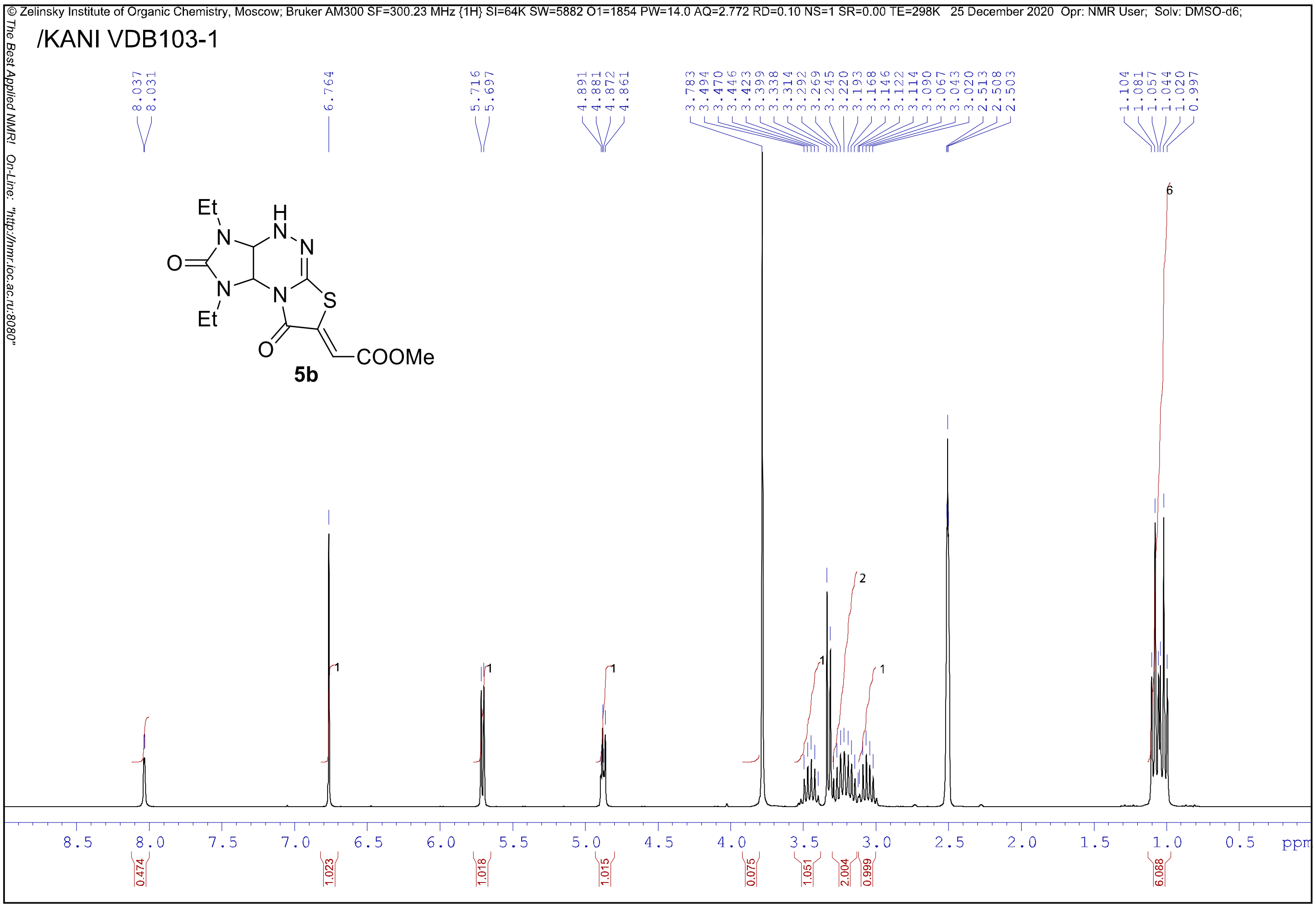

^{13}C NMR spectrum of **5b**

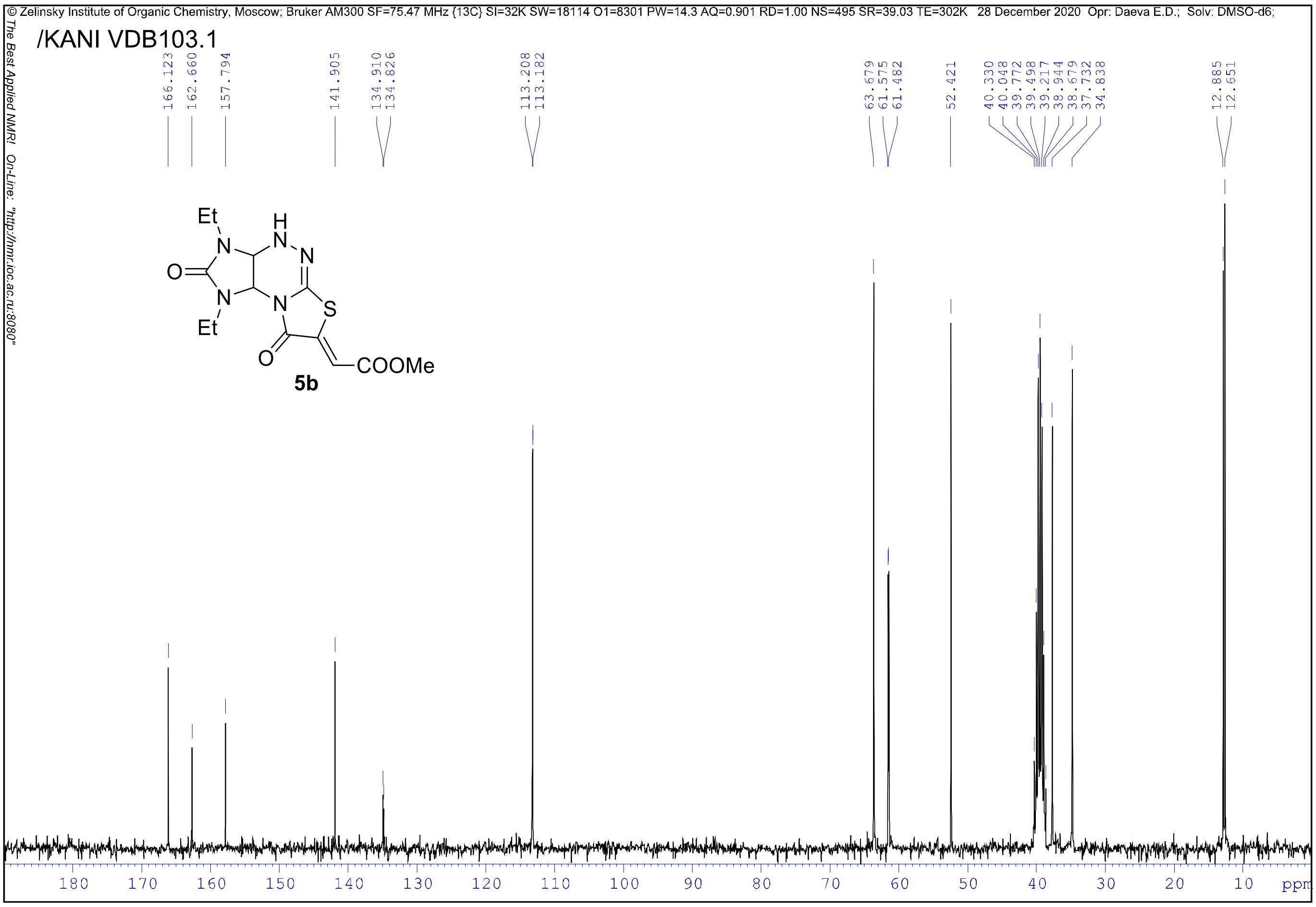

^{1}H NMR spectrum of **5c**

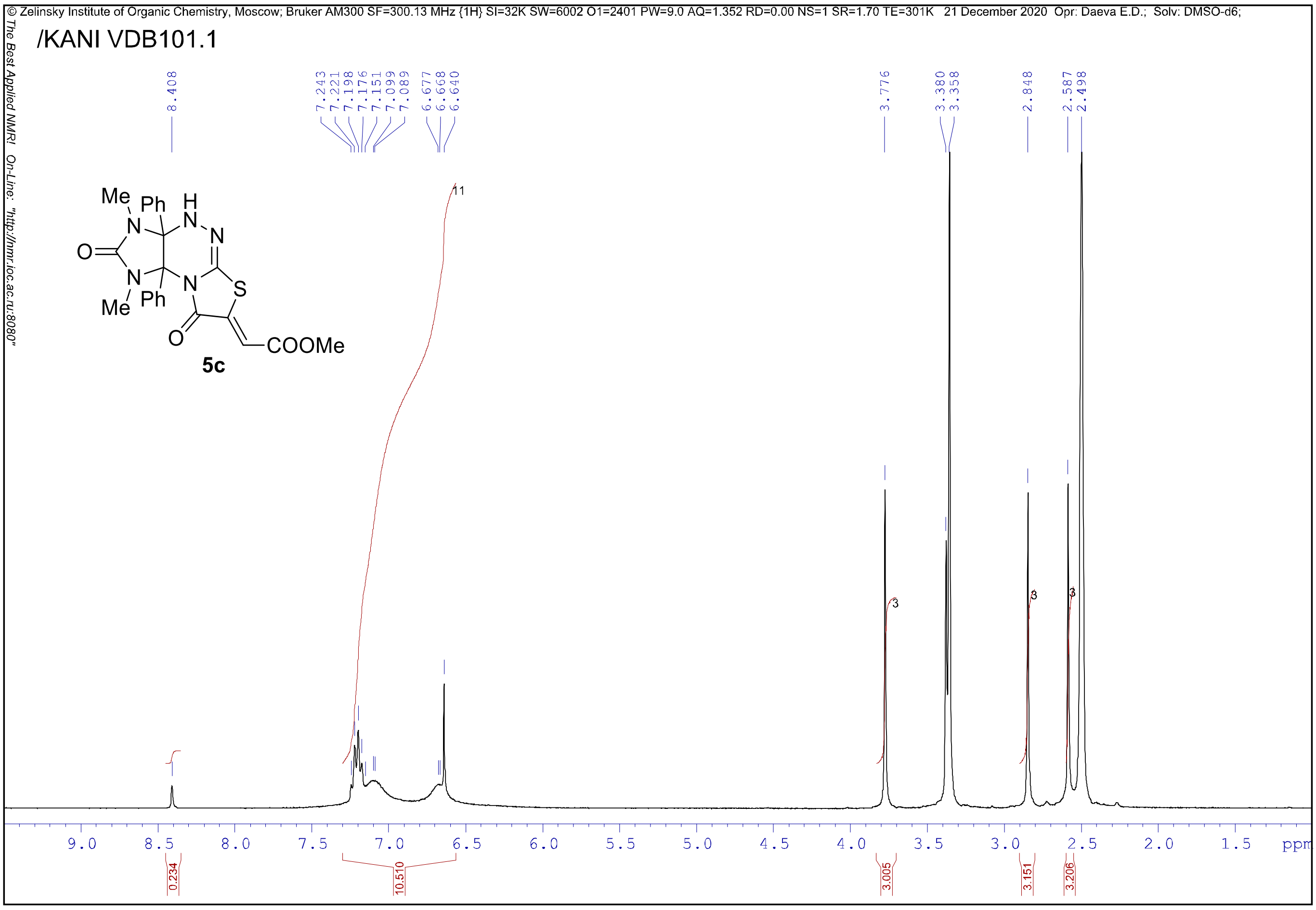

^{13}C NMR spectrum of **5c**

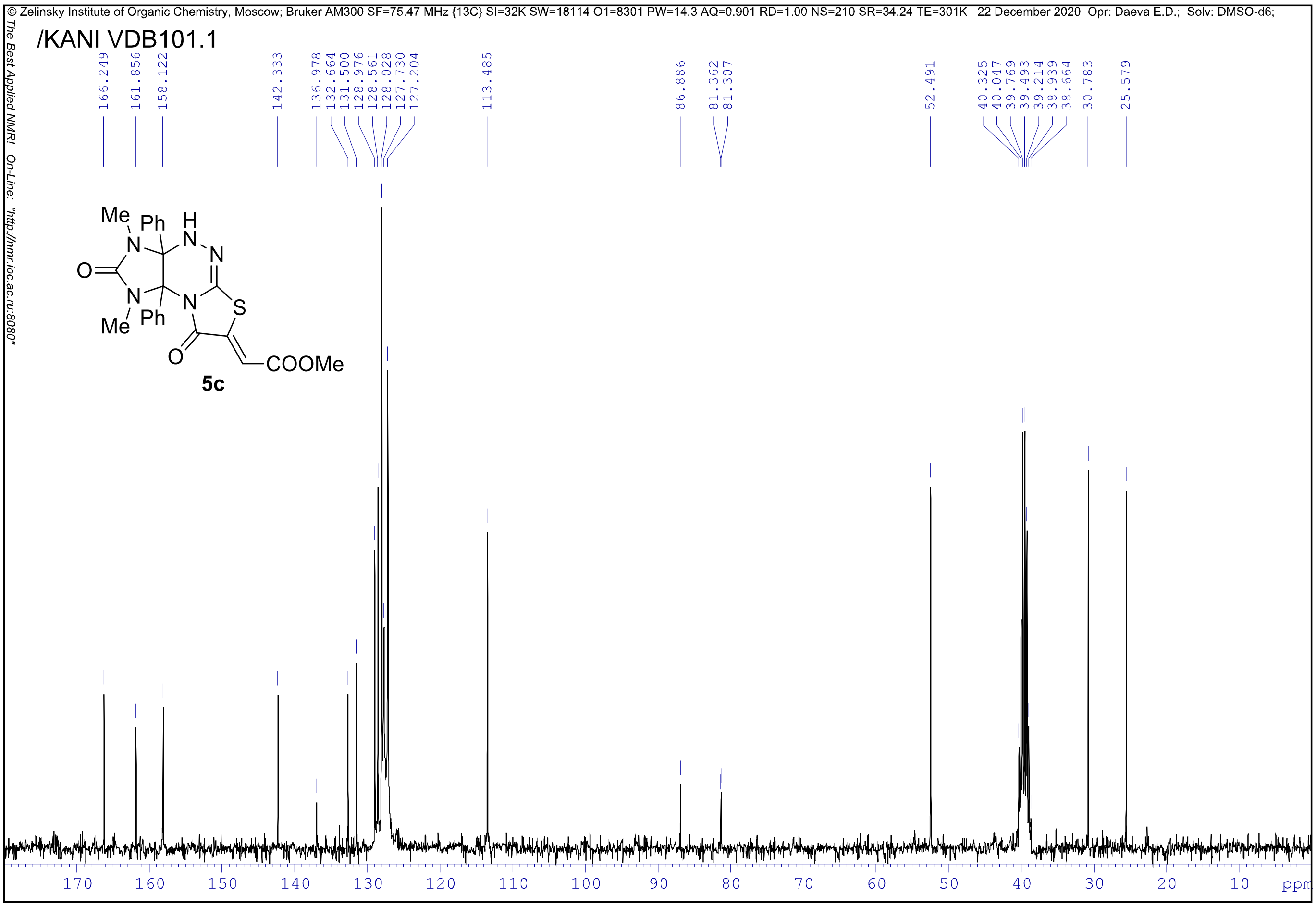

^{1}H NMR spectrum of **5d**

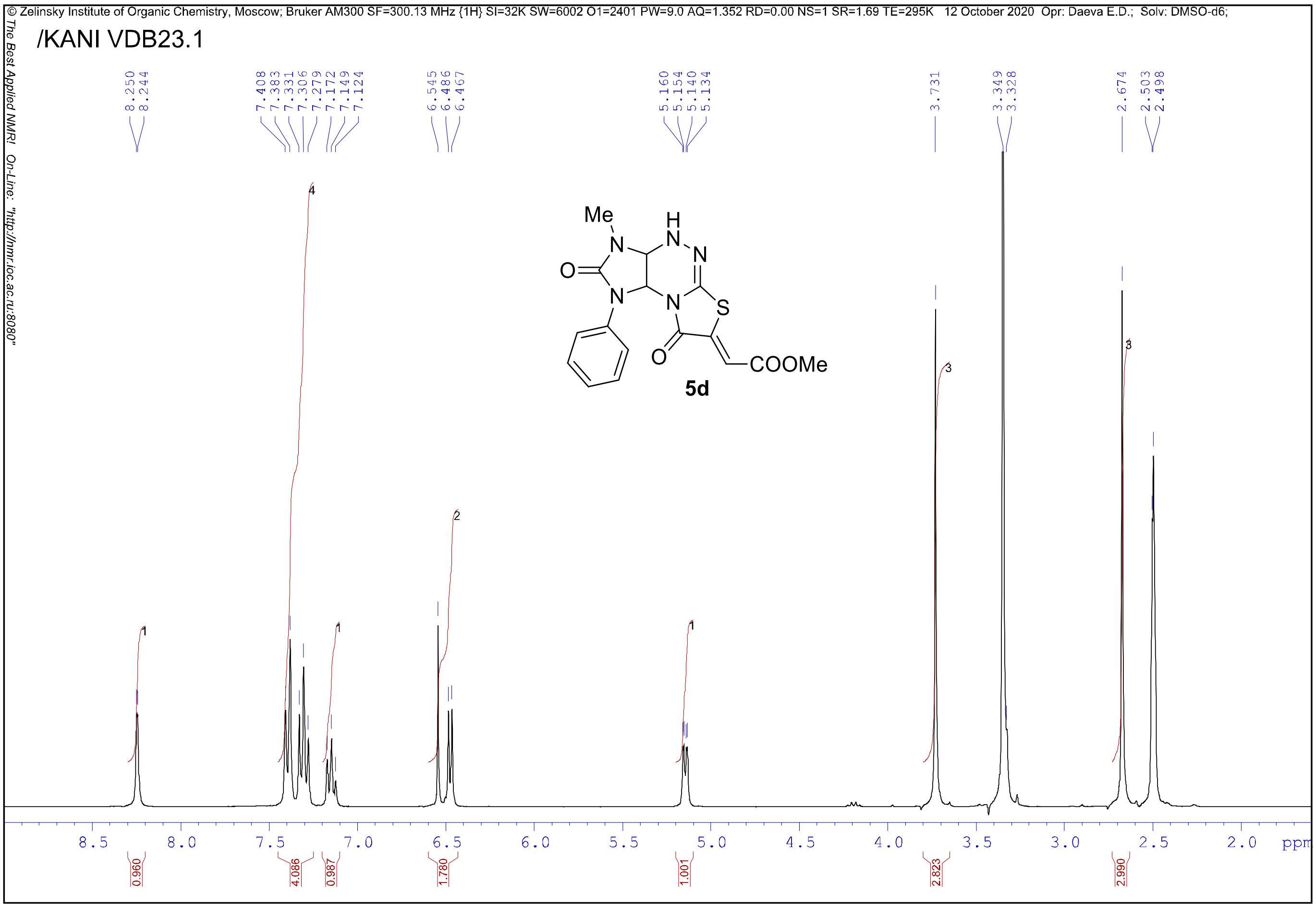

^{13}C NMR spectrum of **5d**

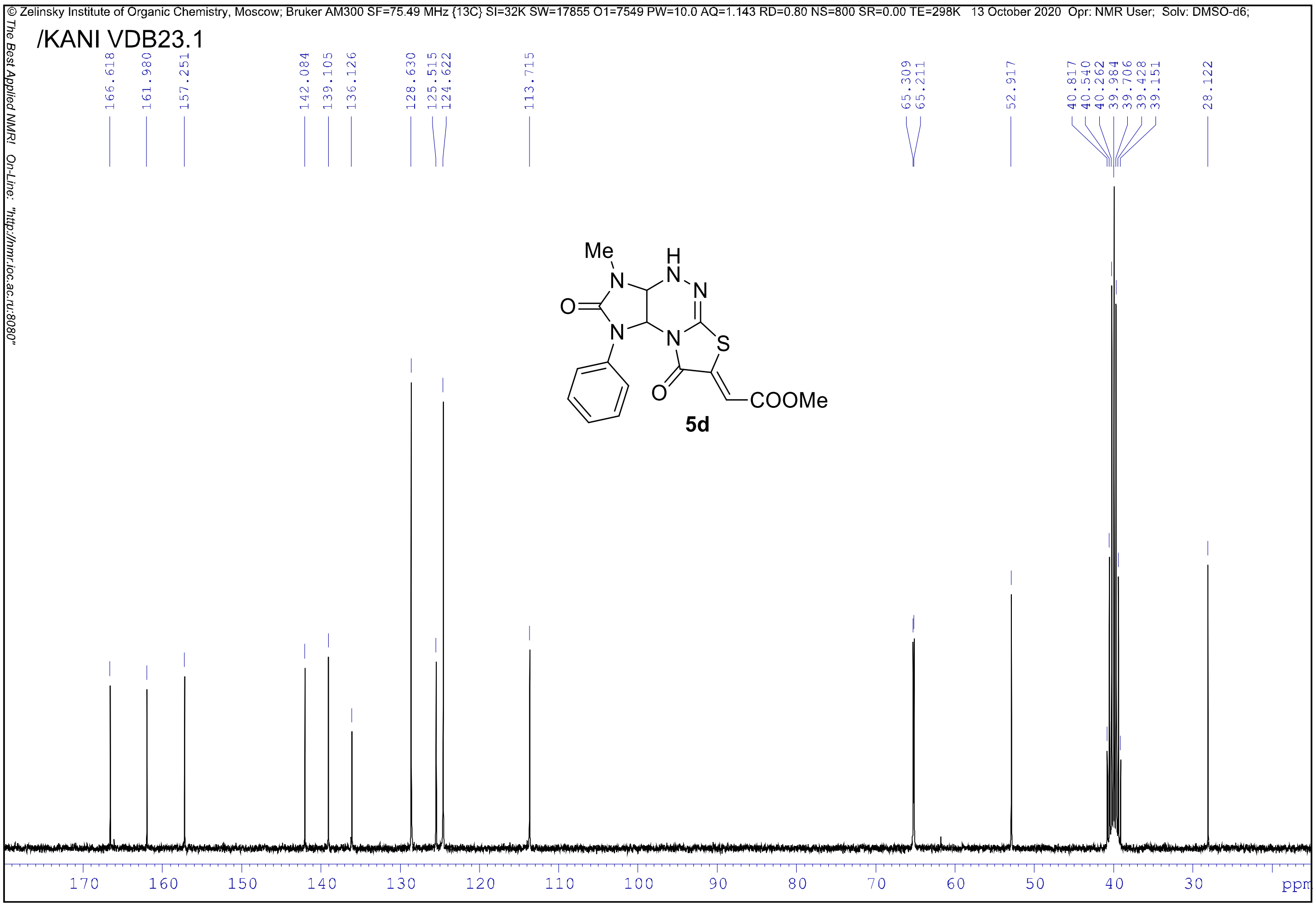

^{1}H NMR spectrum of **5e**

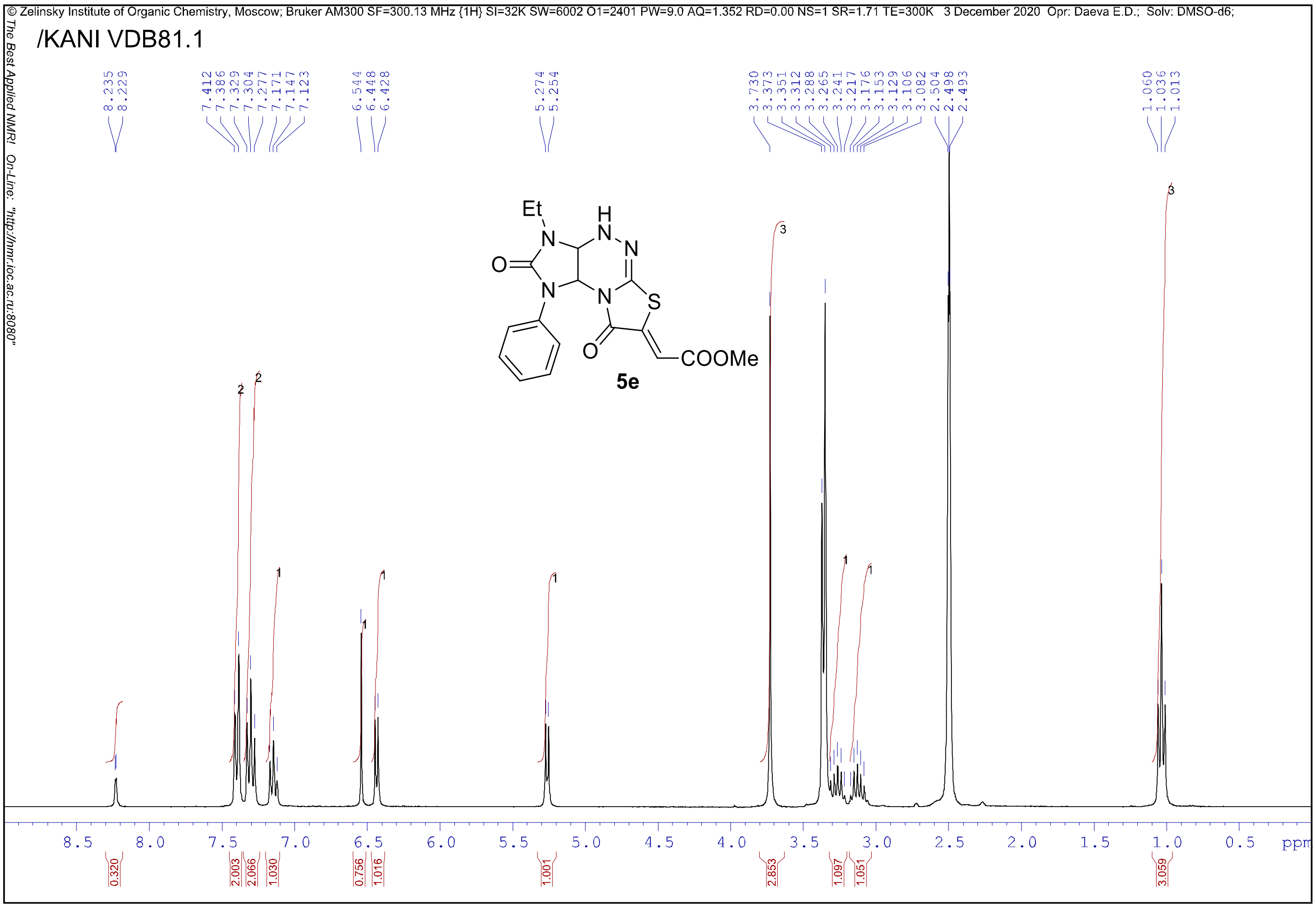

^{13}C NMR spectrum of **5e**

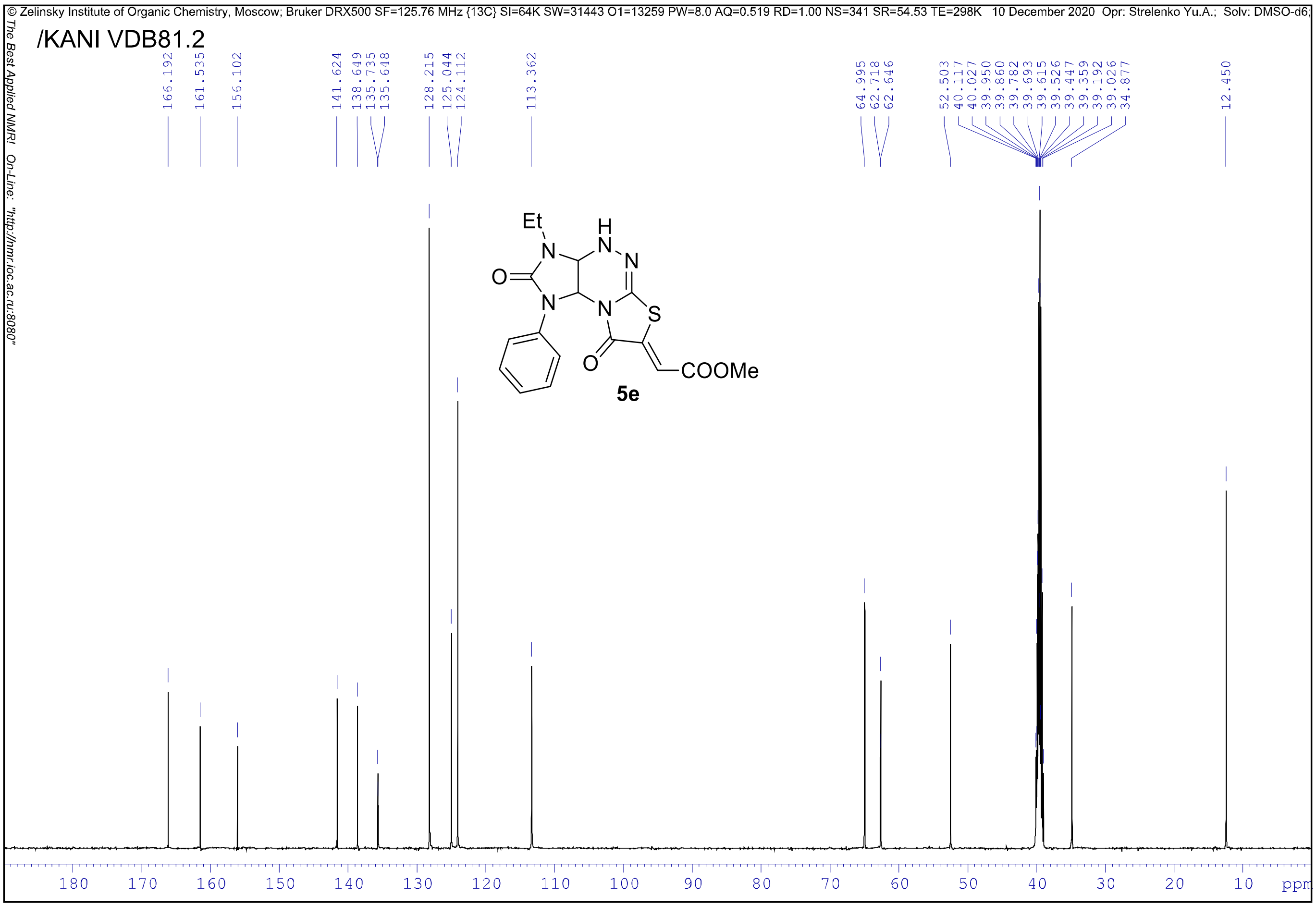

^{1}H NMR spectrum of **5f**

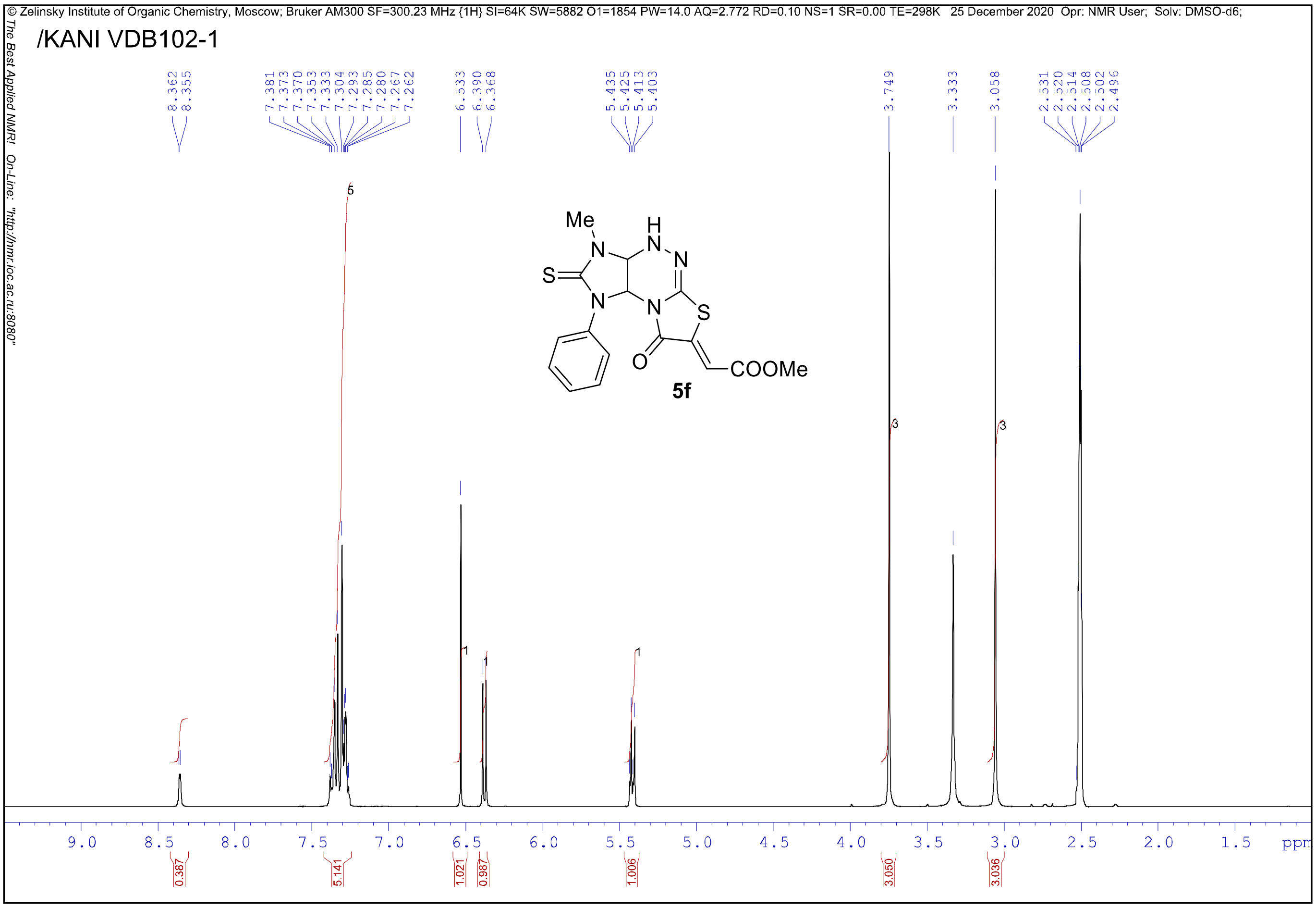

^{13}C NMR spectrum of **5f**

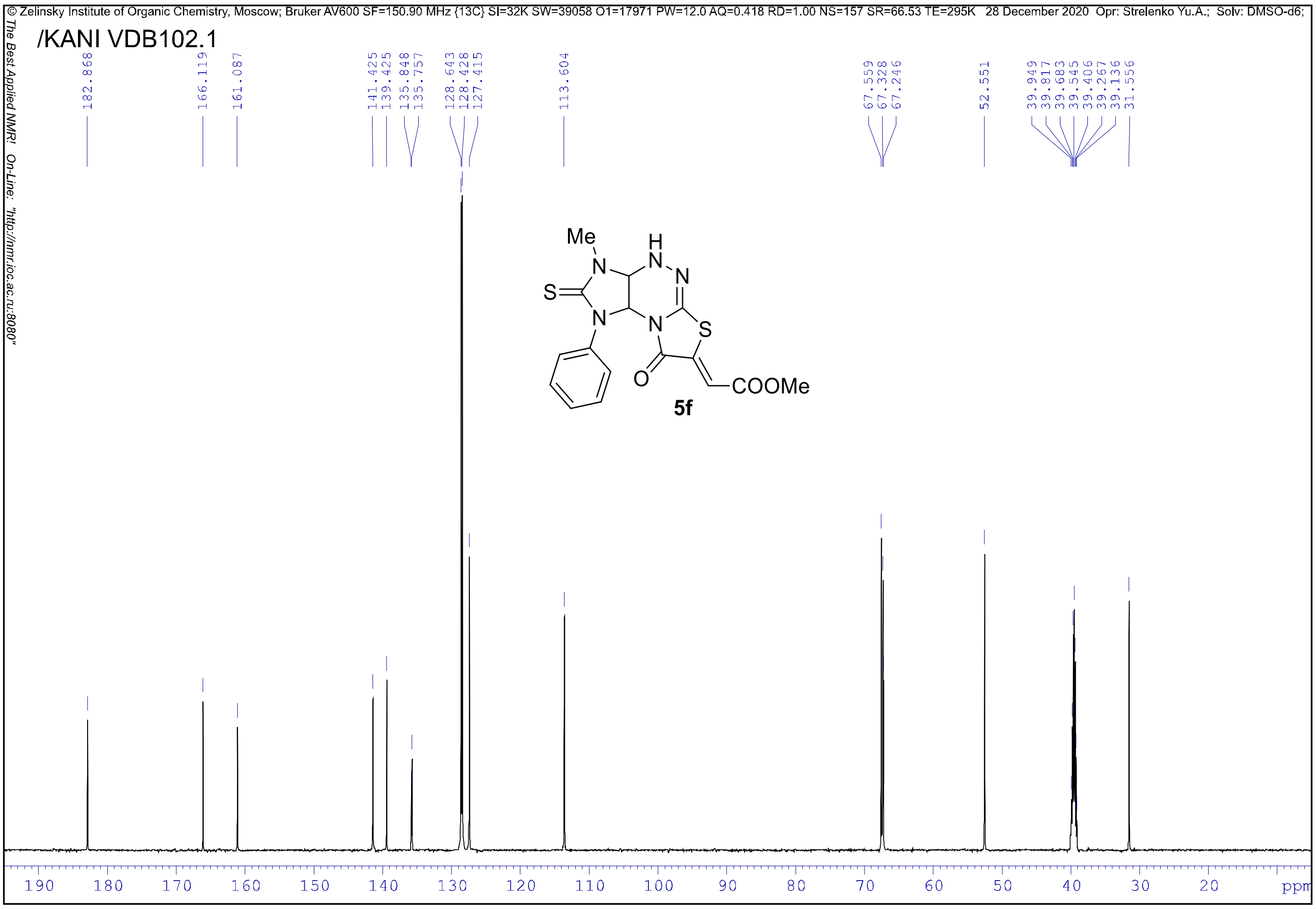

^{1}H NMR spectrum of **5g**

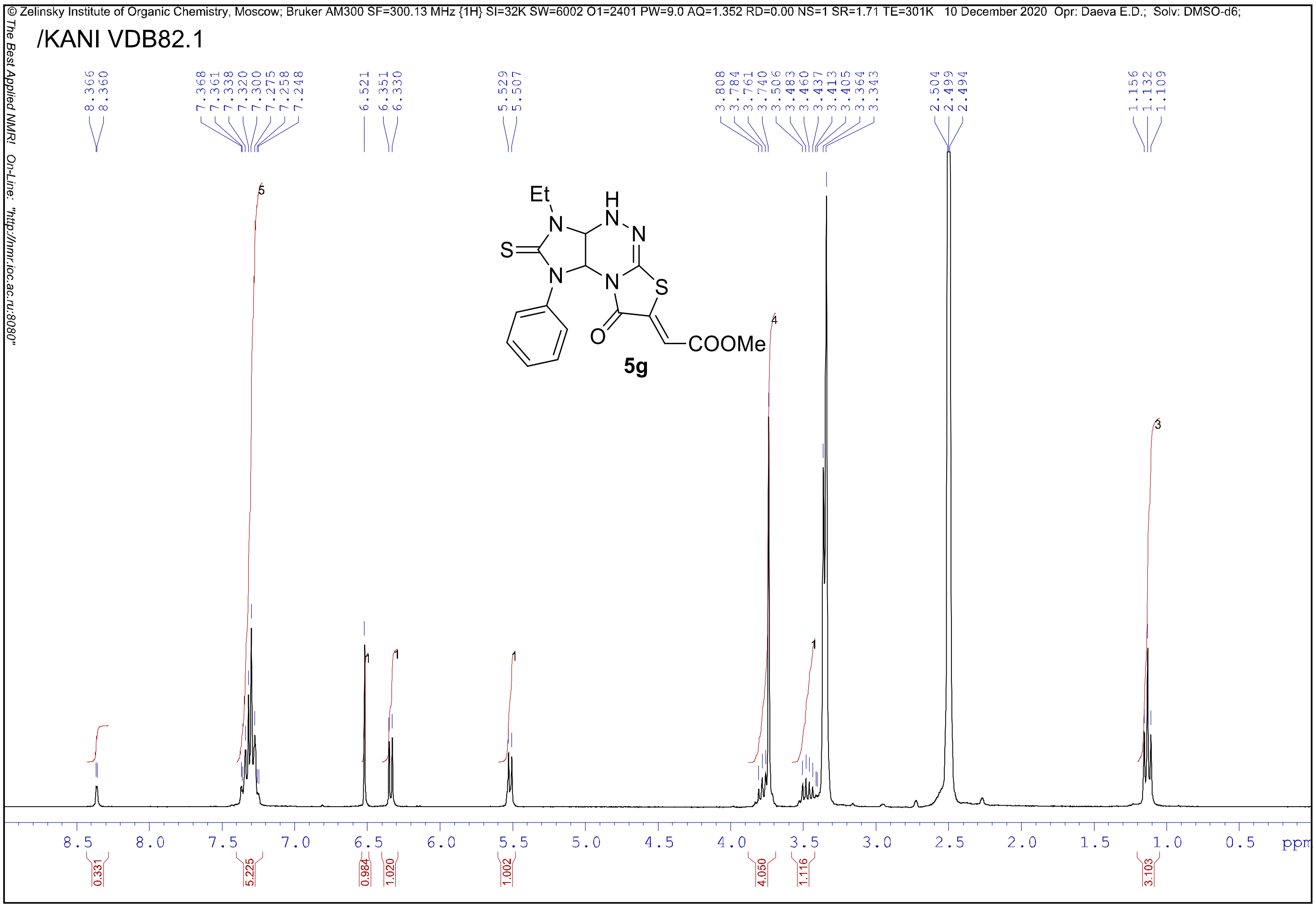

^{13}C NMR spectrum of **5g**

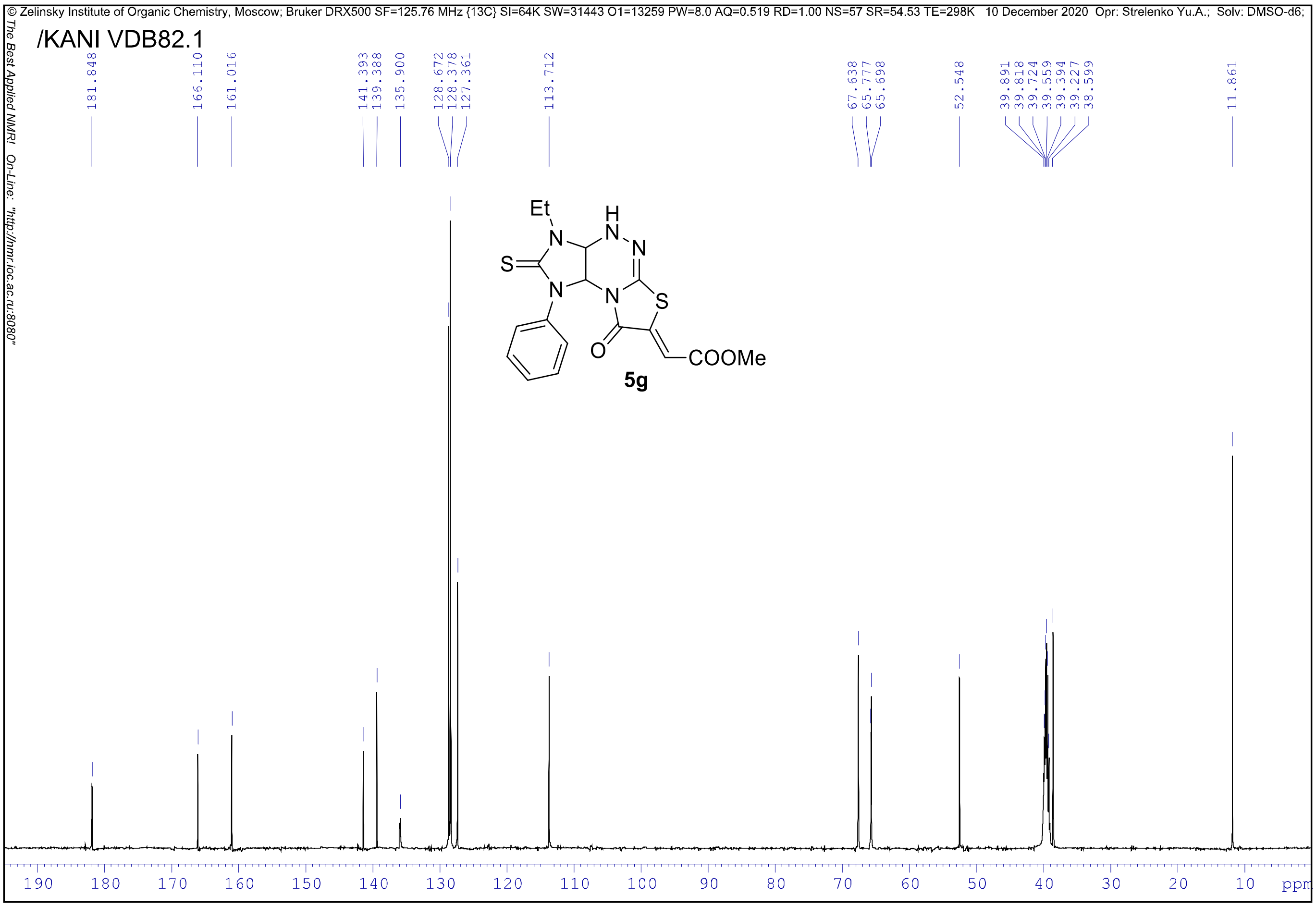

^{1}H NMR spectrum of **5h**

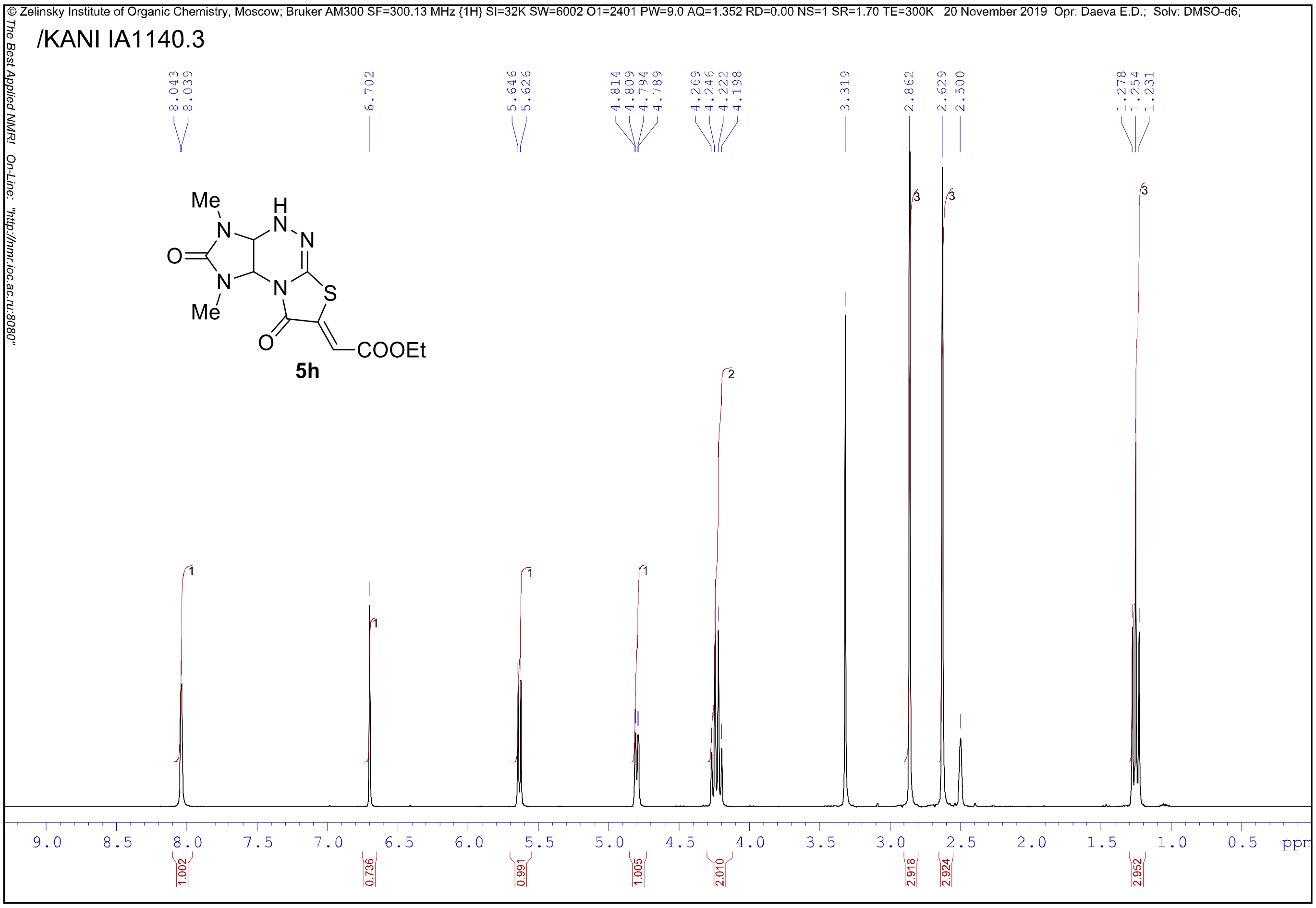

^{13}C NMR spectrum of **5h**

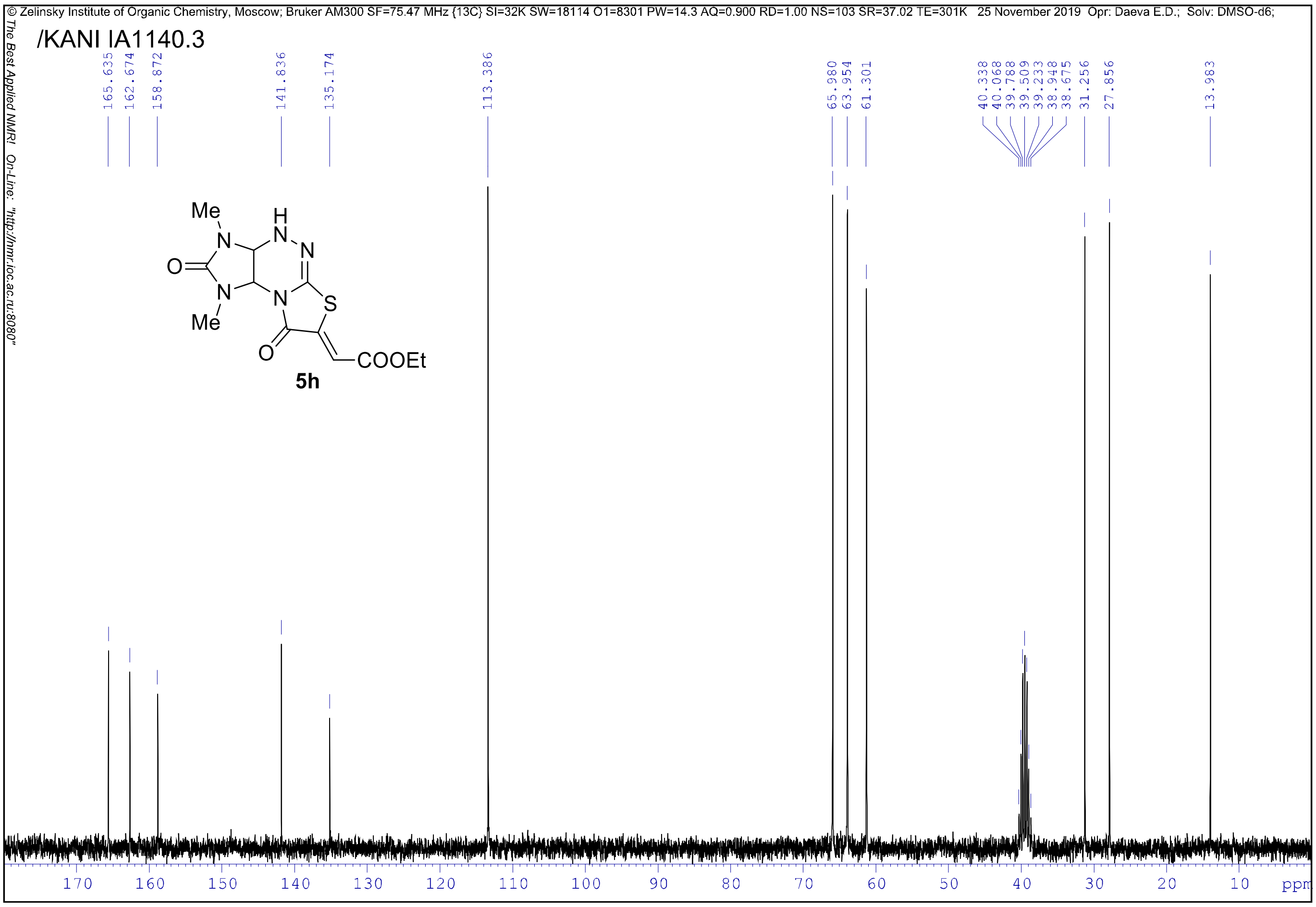

^{1}H NMR spectrum of **5i**

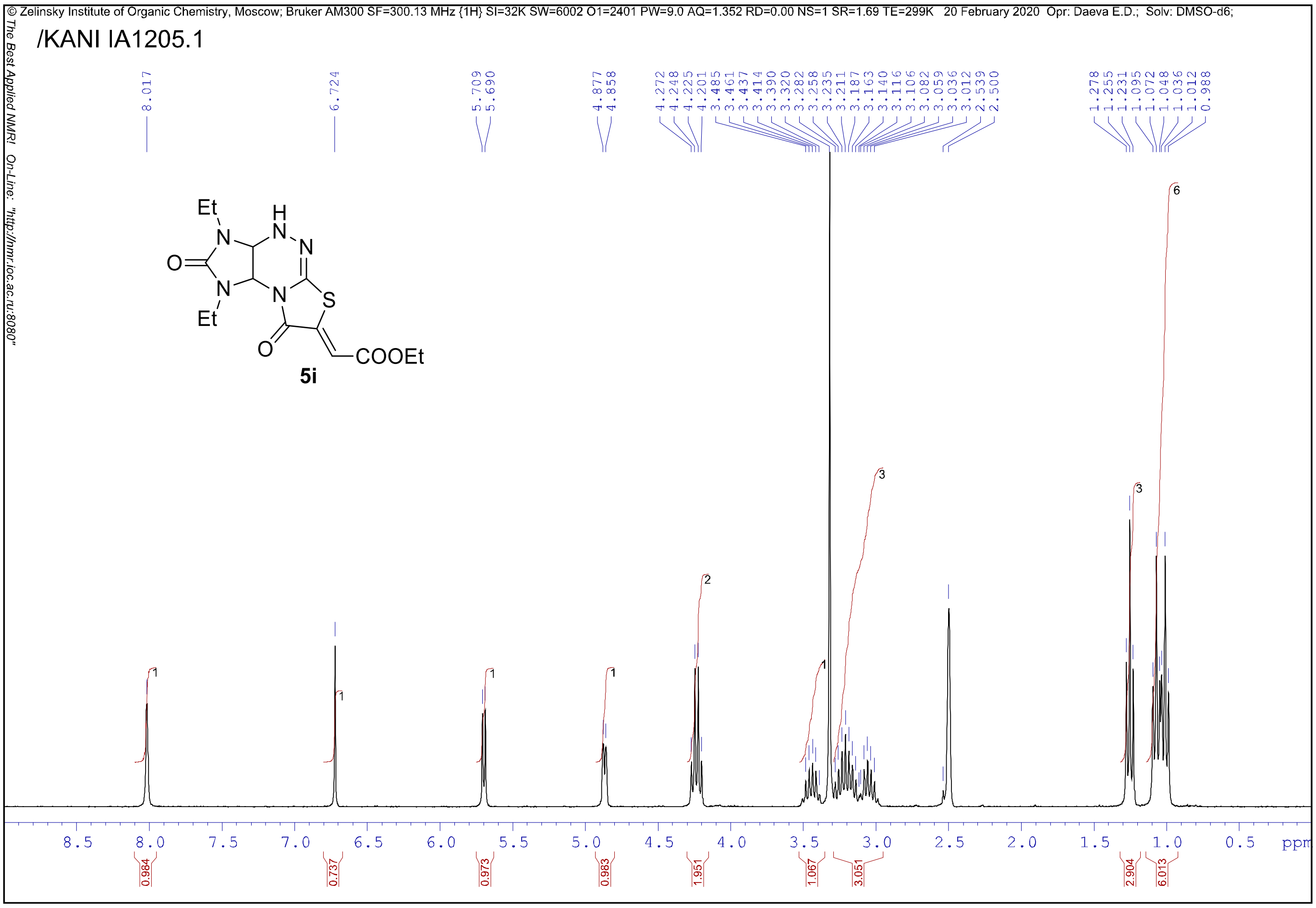

^{13}C NMR spectrum of **5i**

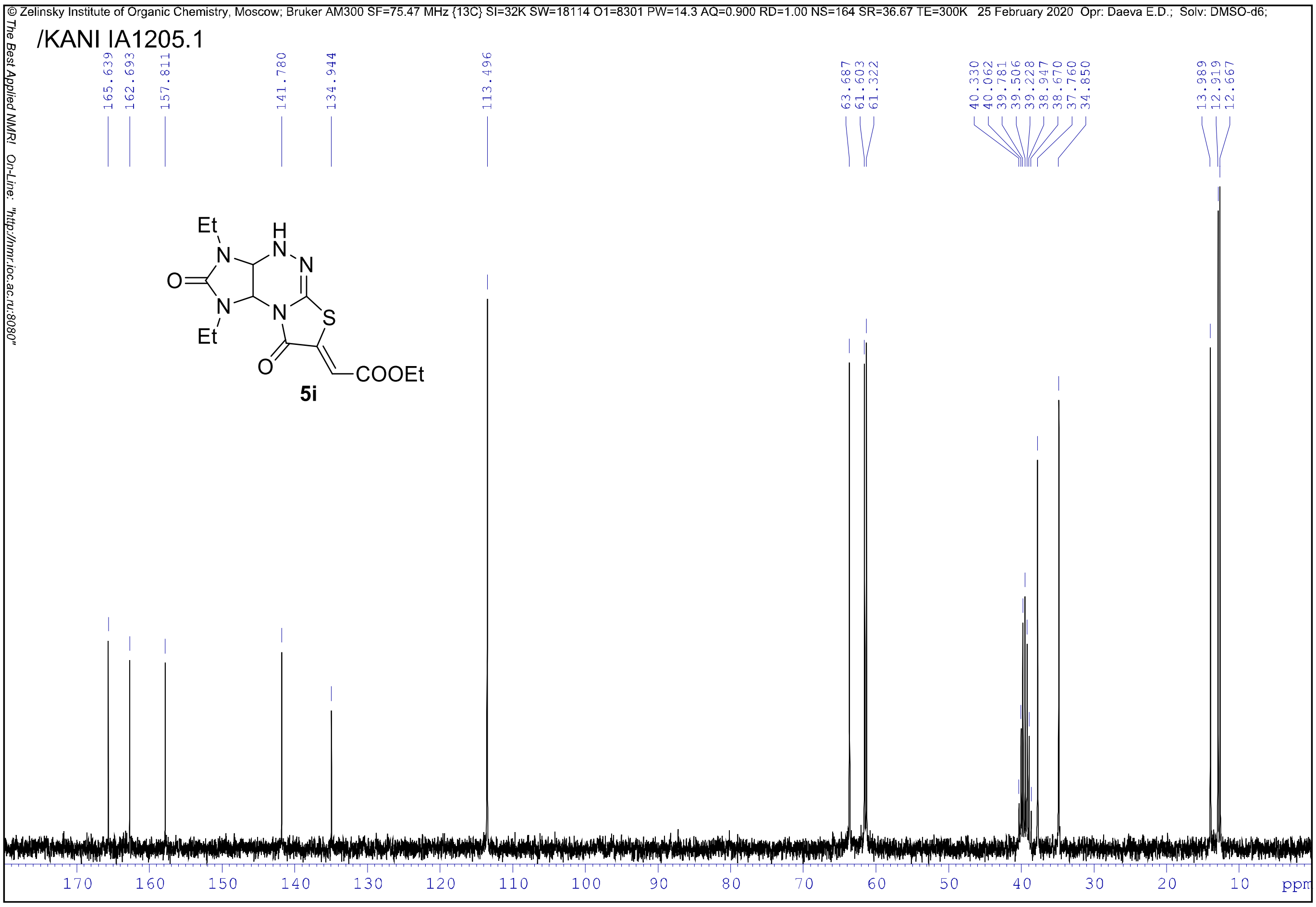

^{1}H NMR spectrum of **5j**

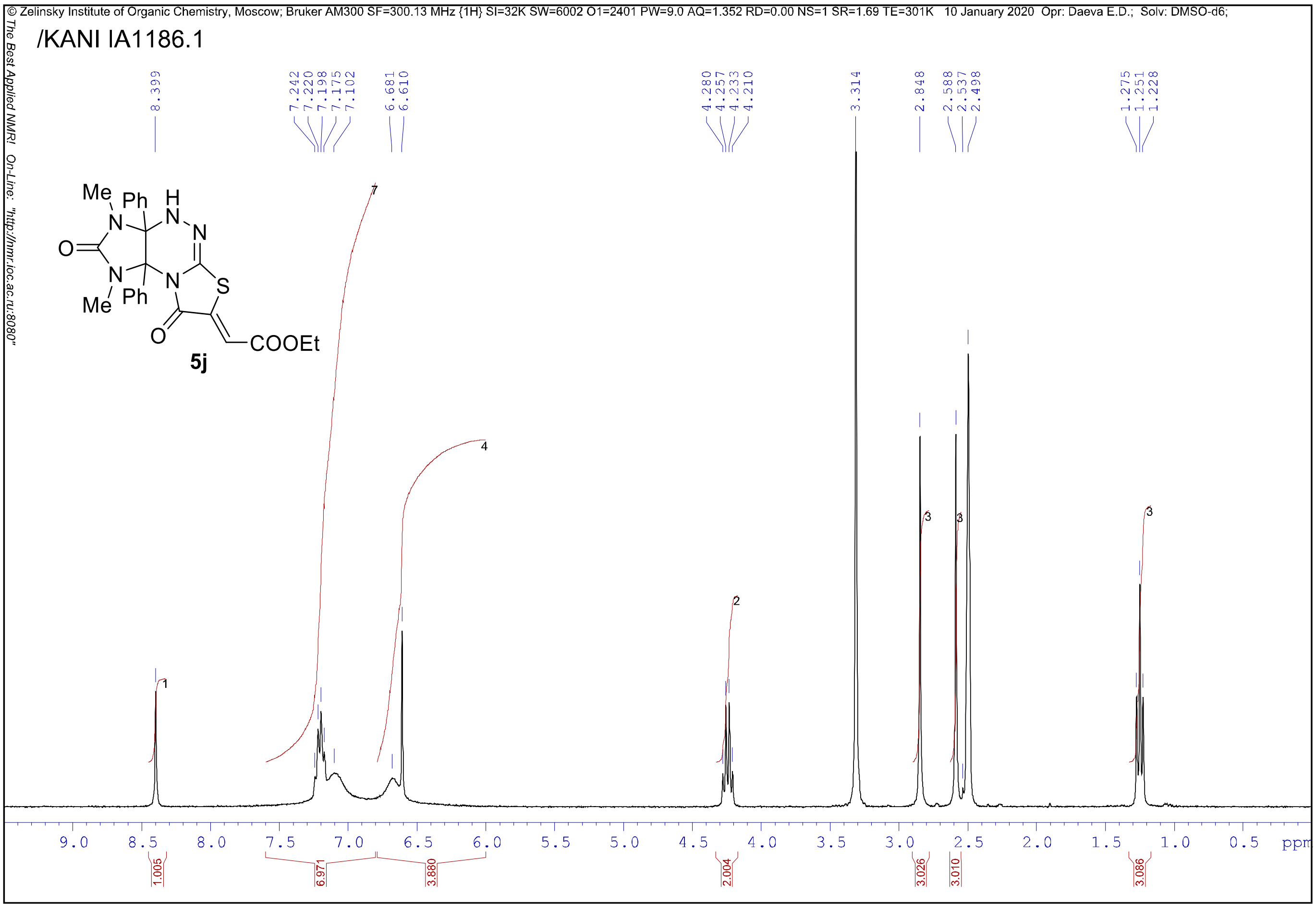

^{13}C NMR spectrum of **5j**

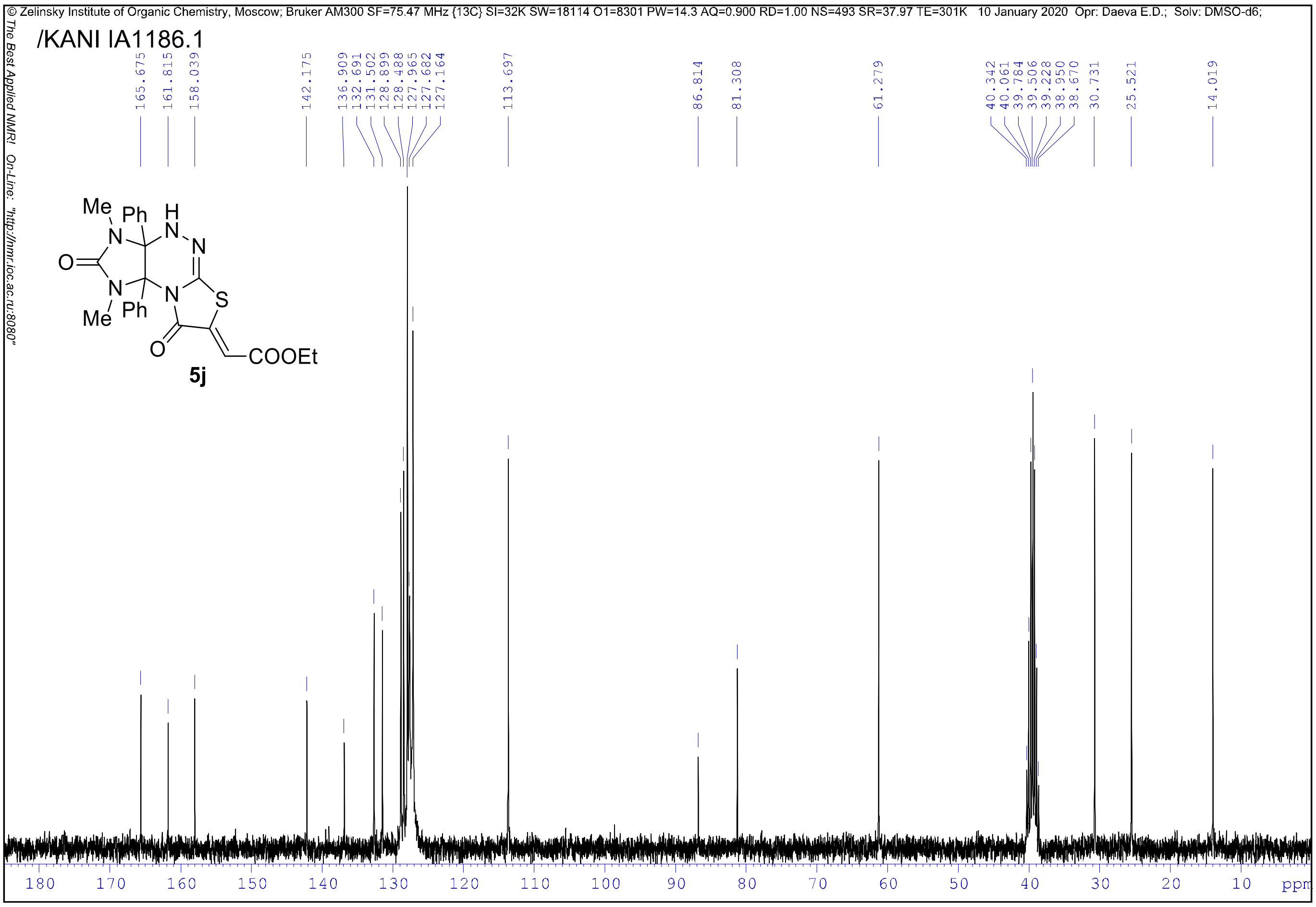

^{1}H NMR spectrum of **5k**

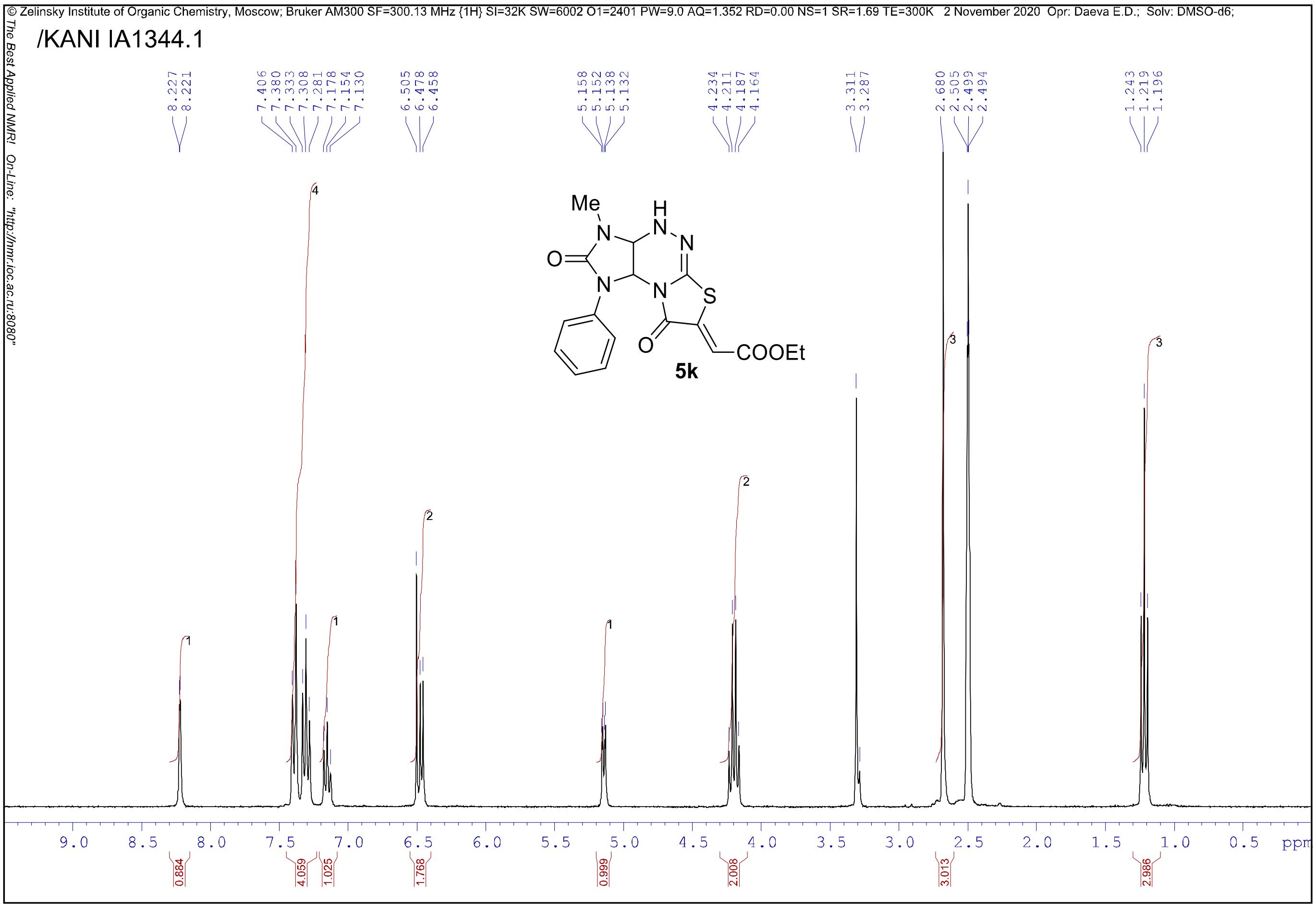

^{13}C NMR spectrum of **5k**

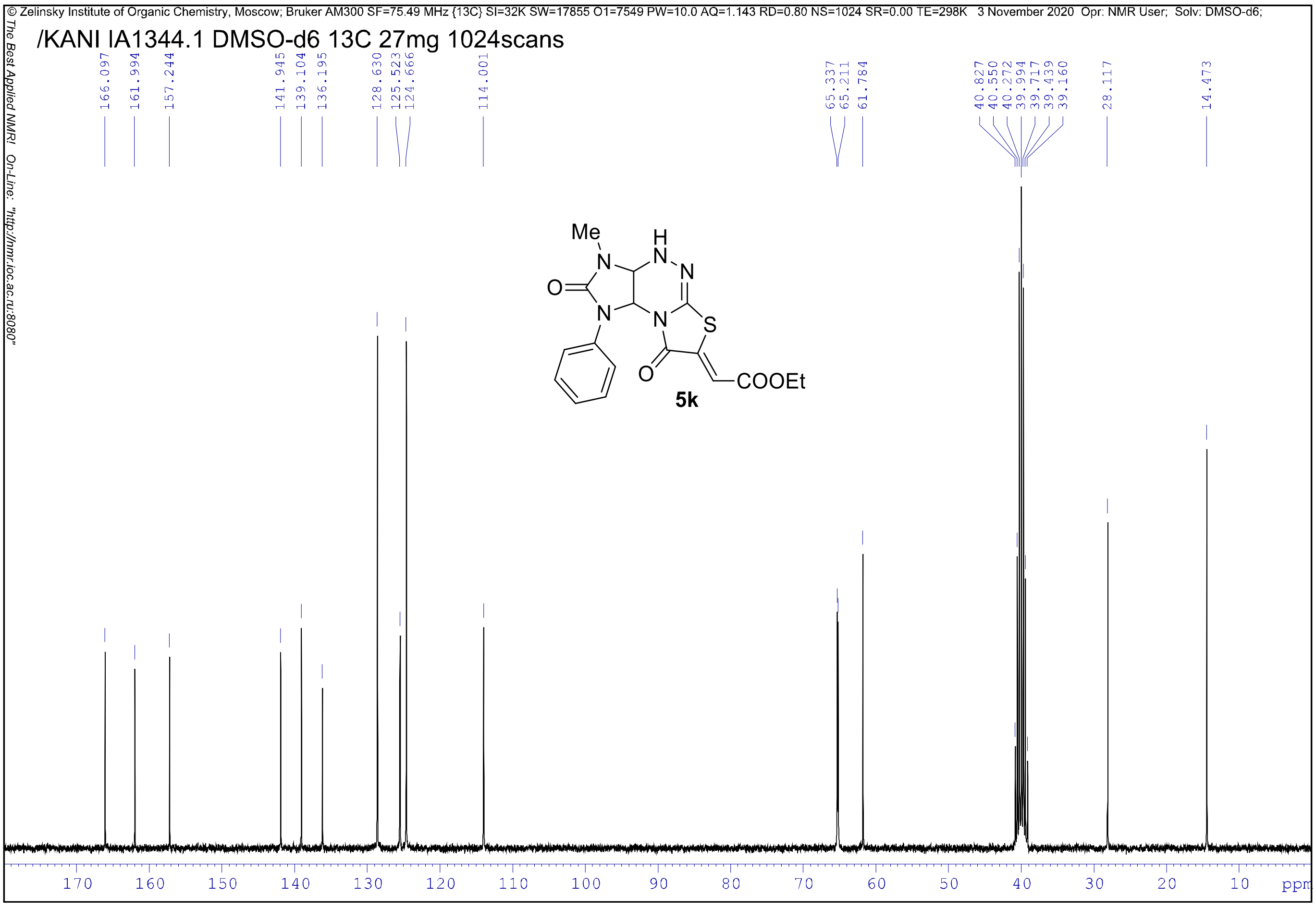

^{1}H NMR spectrum of **5l**

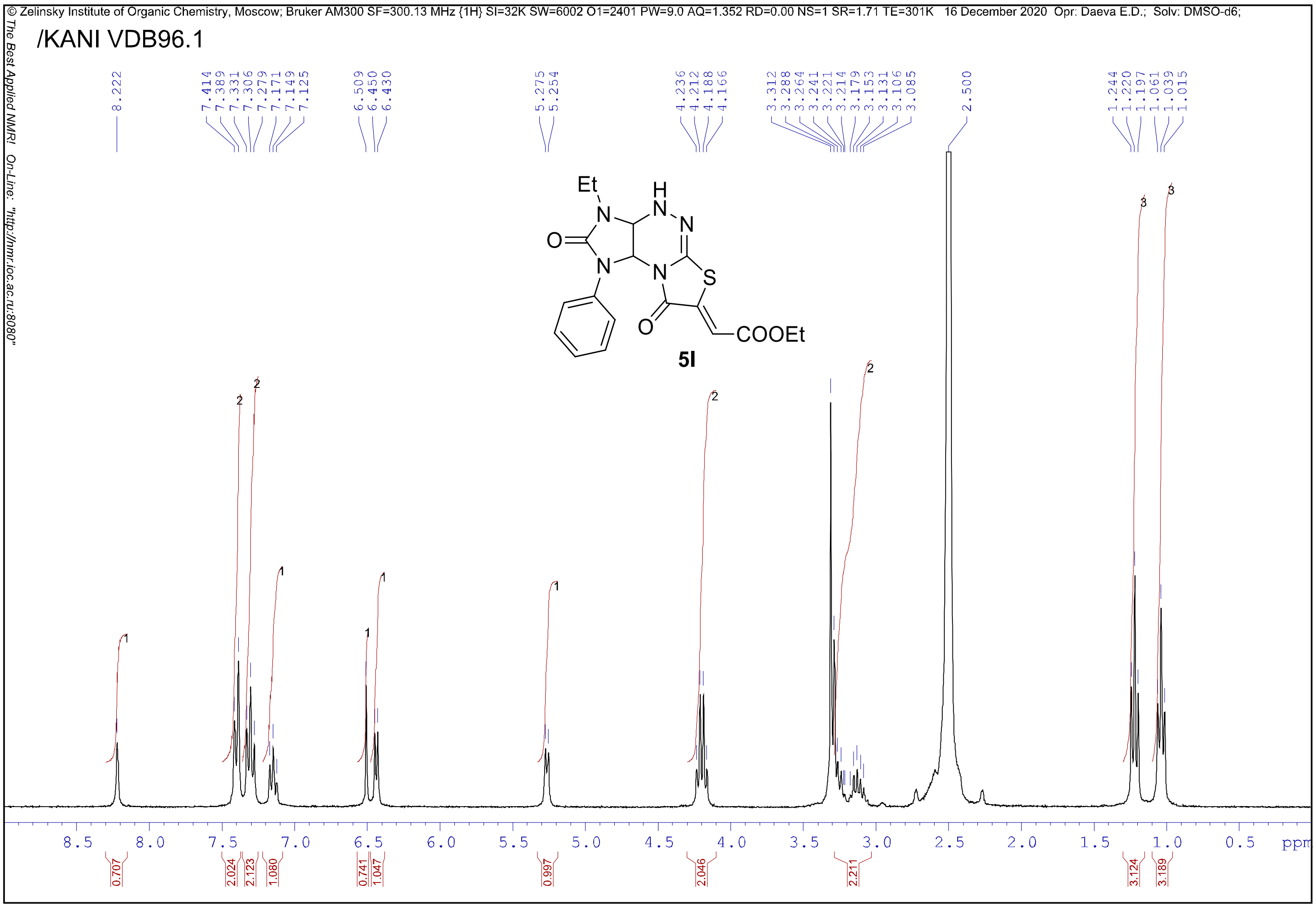

^{13}C NMR spectrum of **5l**

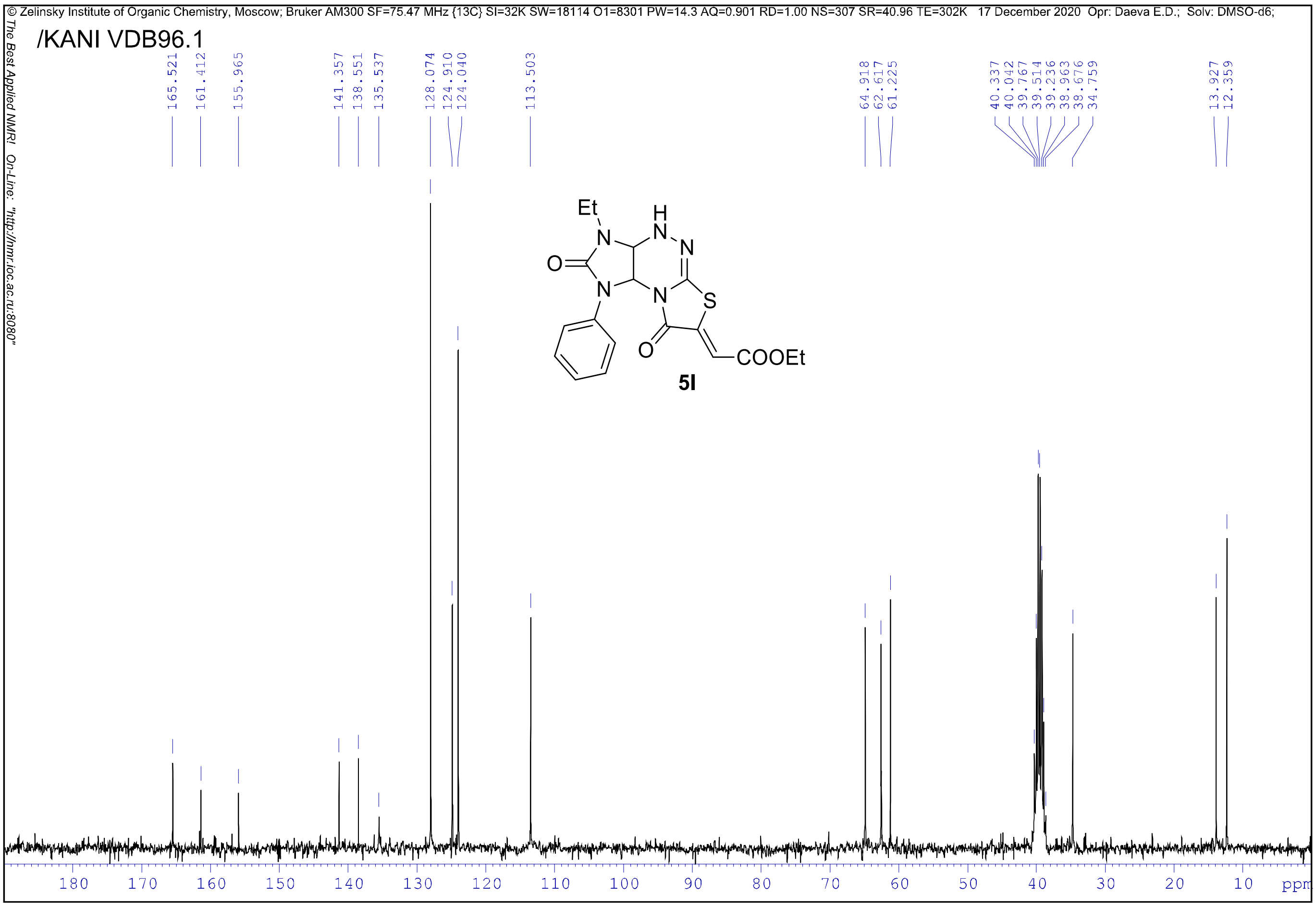

^{1}H NMR spectrum of **5m**

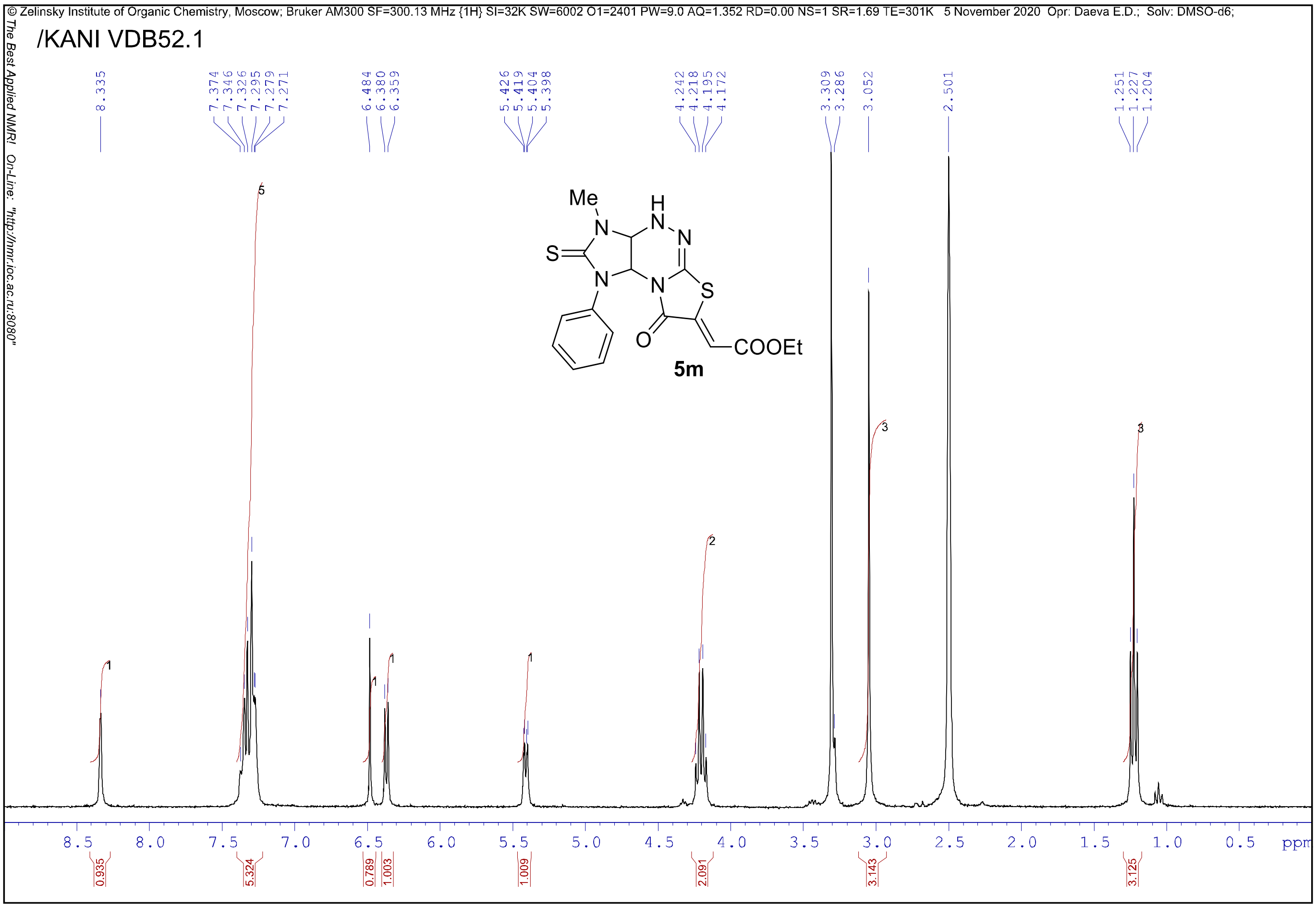

^{13}C NMR spectrum of **5m**

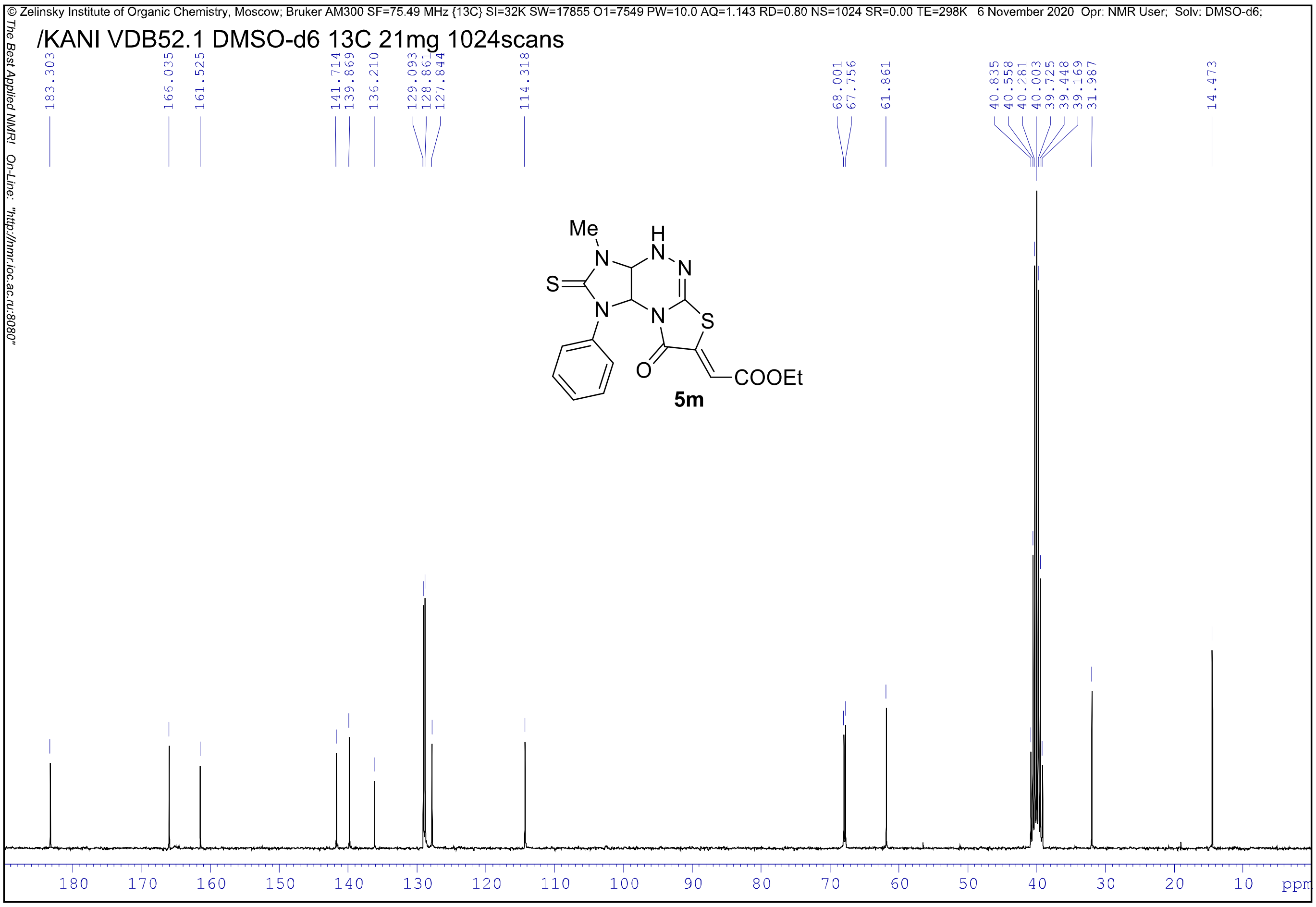

^{1}H NMR spectrum of **5n**

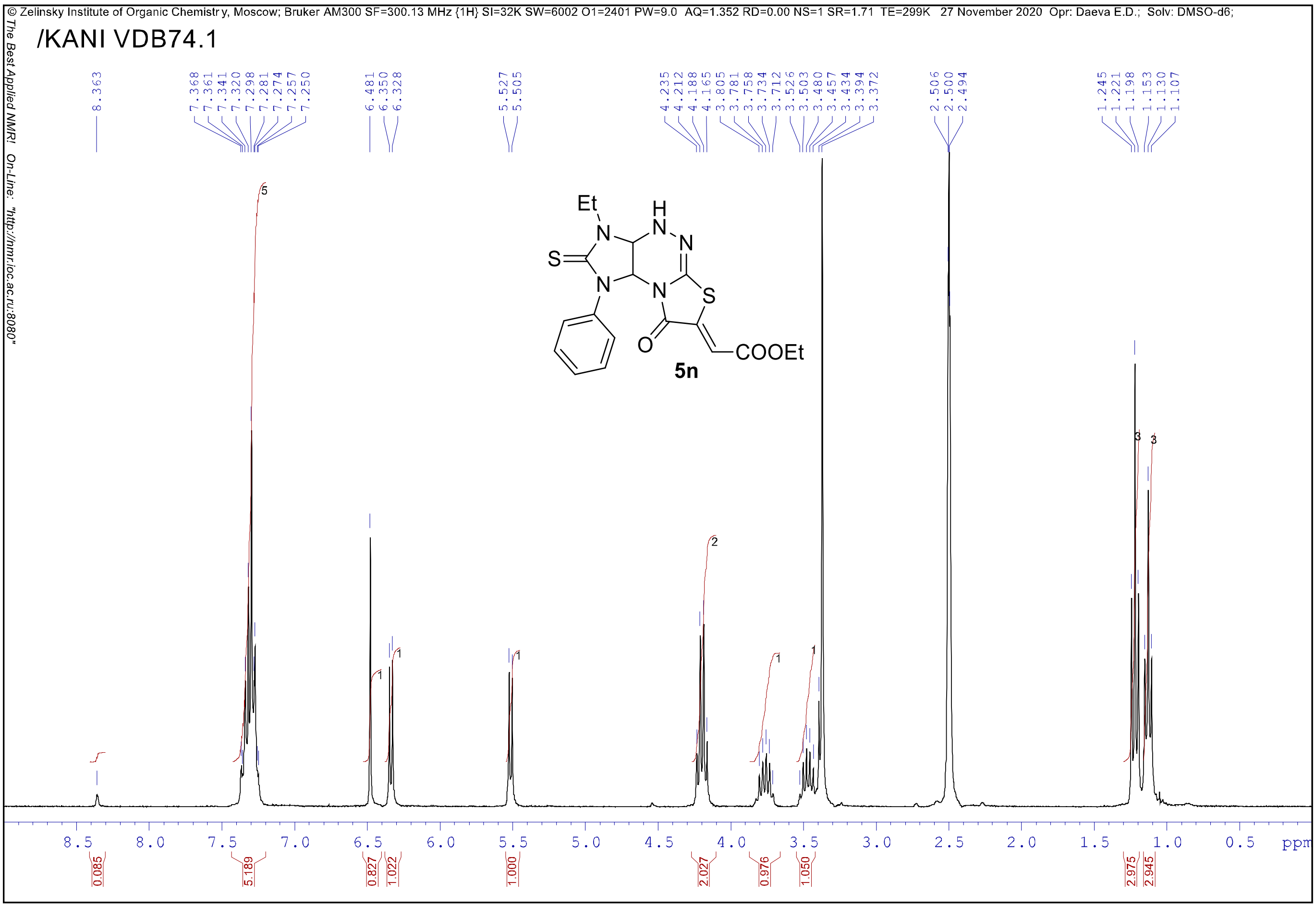

^{13}C NMR spectrum of **5n**

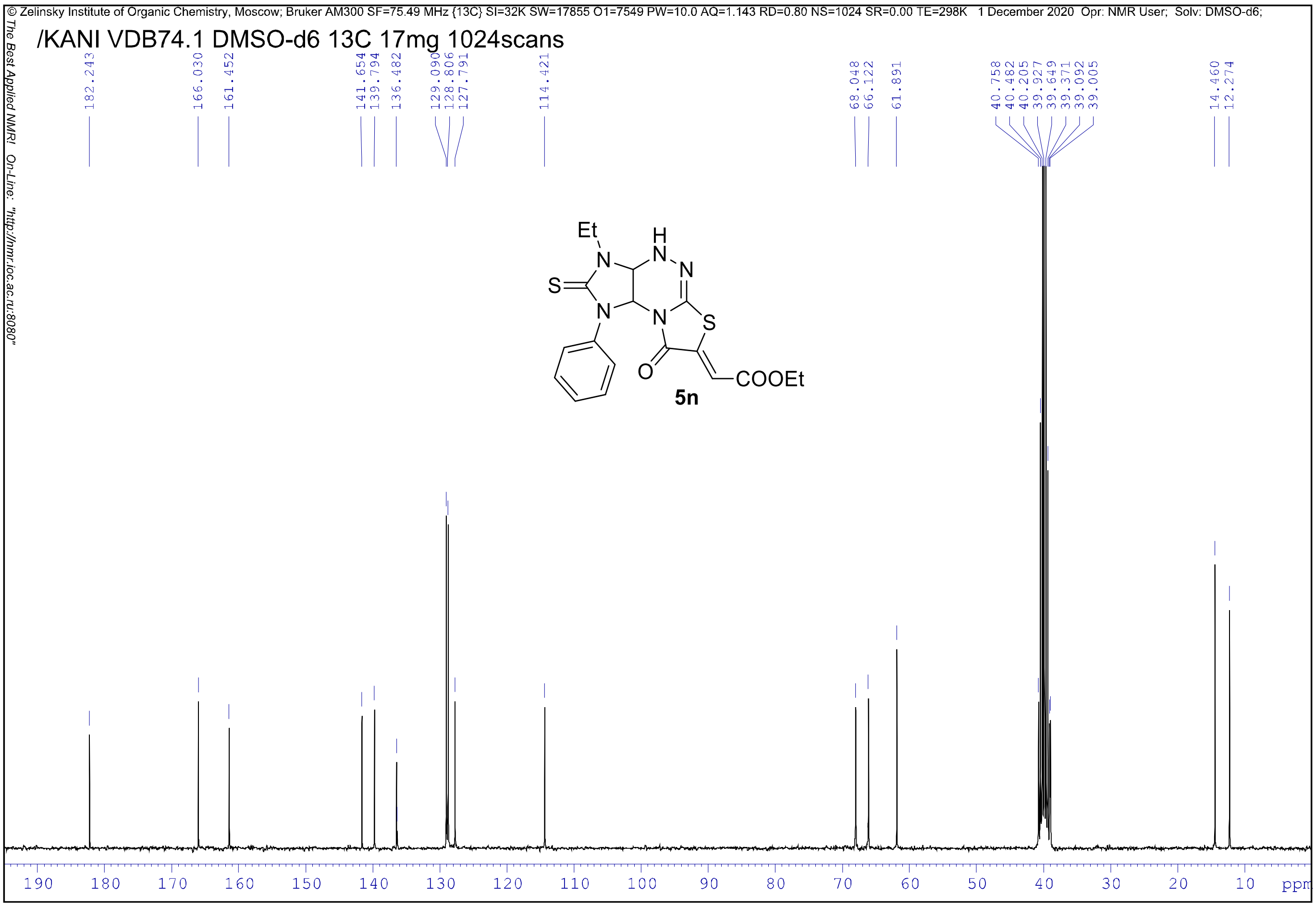

Supplement: File 1 — Experimental and analytical data. [file Beilstein_J_Org_Chem-17-1141-s001.pdf]
